# Programmed intermittent epidural bolus versus continuous epidural infusion combined with patient-controlled epidural analgesia for postoperative analgesia: a meta-analysis of randomized controlled trials

**DOI:** 10.1016/j.bjane.2026.844766

**Published:** 2026-05-23

**Authors:** Gabriel Lemos González, Vinícius Fernandes Sarmento, Bruno Francisco Minetto Wegner, Gustavo Roberto Minetto Wegner, Alesson Marinho Miranda, Iaci Luisa Lopes de Mattos, Tatiana Souza do Nascimento

**Affiliations:** a Universidade Federal do Estado do Rio de Janeiro (UNIRIO), Rio de Janeiro, RJ, Brazil; b Hospital do Servidor Público Estadual de São Paulo, São Paulo, SP, Brazil; c Universidade Federal do Rio Grande do Sul, Porto Alegre, RS, Brazil; d Universidade Federal da Fronteira Sul, Chapecó, SC, Brazil; e Universidade Federal de São Paulo, São Paulo, SP, Brazil; f University of Iowa, Iowa, USA

**Keywords:** Continuous epidural infusion, Epidural analgesia, Patient-controlled epidural analgesia, Postoperative pain, Programmed intermittent epidural bolus

## Abstract

**Background:**

Programmed Intermittent Epidural Bolus (PIEB) and Continuous Epidural Infusion (CEI) are widely used strategies for maintaining postoperative analgesia, typically combined with Patient-Controlled Epidural Analgesia (PCEA). Although PIEB is theorized to enhance epidural drug distribution and analgesic effectiveness through intermittent high-pressure boluses, evidence supporting its superiority over CEI in the postoperative setting remains inconsistent.

**Methods:**

We conducted a systematic review and meta-analysis of Randomized Controlled Trials (RCTs) comparing PIEB and CEI, both with PCEA, in adult postoperative patients (PROSPERO CRD420251046001). PubMed, Embase, and Cochrane were searched until May 1, 2025. Primary outcome: pain at 24 hours. Secondary outcomes: pain at other intervals, anesthetic consumption, and adverse events. Data were synthesized using a random-effects model; Risk of Bias (RoB-2) and small-study effects were assessed.

**Results:**

Ten RCTs comprising 692 patients were included. No significant difference was found in 24-hour pain scores at rest (MD = -0.45; 95% CI -0.99 to 0.10) or movement (MD = -0.88; 95% CI -2.07 to 0.32). PIEB showed mildly lower pain at other time points and reduced total epidural volume (MD = -7.31 mL; 95% CI -13.7 to -0.92 mL). However, PIEB was associated with higher hypotension risk (RR = 1.71; 95% CI 1.05 to 2.78). No differences were found in opioid consumption, PCEA demands, or other adverse events.

**Conclusion:**

PIEB offers no significant analgesic advantage and increases hypotension risk compared to CEI in postoperative settings, when both are combined with PCEA. These findings should be interpreted with caution due to the limited number of studies and substantial heterogeneity.

**PROSPERO registry:**

https://www.crd.york.ac.uk/PROSPERO/view/CRD420251046001.

## Introduction

The effective management of acute postoperative pain remains a major challenge in anesthesiology. Despite decades of pharmacologic and procedural advances, inadequate pain relief continues to hinder functional recovery, often prolonging hospitalization by limiting oral intake and early mobilization.^1^^,^^2^ Importantly, severe pain within the first 24 hours post-surgery is a known risk factor for the development of chronic pain and long-term impairment in quality of life.^2^, ^3^, ^4^

While various regional techniques have emerged, neuraxial analgesia delivered through an epidural catheter remains a widely implemented choice for major postoperative pain management.^5^^,^^6^ Its efficacy depends on multiple procedural factors, including the level of injection, the composition and concentration of the anesthetic solution, and the method of drug administration.^7^^,^^8^ Although single-bolus injections can be used, continuous infusions generally provide more stable and sustained analgesia for up to 72 hours after surgery compared with single-shot or systemic approaches.^9^

Modern infusion pumps enable two main modalities for maintaining baseline epidural analgesia: Continuous Epidural Infusion (CEI) and Programmed Intermittent Epidural Bolus (PIEB). CEI delivers a constant flow of medication, whereas PIEB administers pre-programmed boluses at fixed intervals. Both methods are frequently supplemented with Patient-Controlled Epidural Analgesia (PCEA) to manage breakthrough pain.^7^

In labor analgesia, combining PIEB or CEI with PCEA has been shown to enhance pain relief compared with traditional techniques, with PIEB demonstrating superior outcomes to CEI in several trials.^10^, ^11^, ^12^ However, postoperative and labor pain represent distinct clinical entities. Labor pain is a unique physiological process modulated by individual perceptions and expectations of childbirth, precluding direct extrapolation of labor findings to surgical populations.^13^ Moreover, although also occurring in the obstetric setting, cesarean delivery constitutes a surgical procedure characterized by tissue injury and postoperative somatic pain, and should be distinguished from the vaginal delivery assessed in labor analgesia studies.^14^ As such, in postoperative settings, available Randomized Controlled Trials (RCTs) comparing PIEB and CEI combined with PCEA remain limited and heterogeneous, and their results have not been systematically integrated.

Therefore, we conducted a systematic review and meta-analysis of RCTs to assess whether PIEB provides improved postoperative analgesia compared with CEI, both combined with PCEA, in adult patients. We evaluated pain at rest and during movement primarily at 24 hours as the primary outcome, and the secondary outcomes included other prespecified postoperative timepoints (12, 36, and 48 hours), anesthetic and opioid consumption, PCEA use, and adverse effects, including Postoperative Nausea and Vomiting (PONV), motor block, pruritus, hypotension, and patient satisfaction.

## Methods

This systematic review and meta-analysis adhered to the Cochrane Handbook and PRISMA guidelines (Supplemental Table S1).^15^^,^^16^ The study protocol was registered at the International Prospective Register of Systematic Reviews (PROSPERO), under the registration number CRD420251046001 on May 5, 2025.

### Eligibility criteria

Eligibility criteria were defined using the PICOS framework. We included studies enrolling adults (≥ 18 years) undergoing surgery (P) who received PIEB (I) or CEI (C), both combined with PCEA. Outcomes of interest (O) included postoperative pain at rest and during movement, anesthetic and opioid consumption, PCEA use, PONV, pruritus, motor block, hypotension, and patient satisfaction. Only peer-reviewed RCTs were eligible for inclusion (S).

We excluded studies with overlapping patient populations or with relevant differences in intraoperative and/or postoperative analgesic protocols beyond the studied interventions (e.g., use of different systemic analgesics between groups and studies presenting intervals with paused epidural infusion), as such differences could confound the comparative assessment of PIEB and CEI.

### Data source and search strategy

We systematically searched PubMed, Embase, and Cochrane Library databases until May 1, 2025. The complete search strategies (free-text and indexed terms) for each database are provided in Supplemental Table S2.

After removing duplicates, two authors (G.L.G. and V.F.S.) screened the titles and abstracts and independently assessed the full-text articles for inclusion based on the prespecified criteria. Discrepancies were resolved through a panel discussion with the senior author (T.S.N.). There was no backward citation tracking or request for data from authors. No automated tool was used, and all screening and selection processes were conducted manually.

### Outcomes of interest

Our prespecified primary endpoint was pain score measured at 24 hours post-surgery. Pain outcomes were divided into rest pain and movement-related pain (elicited by movement).

Secondary outcomes included: 1) Rest pain at 12, 36 and 48 hours; 2) Movement-related pain at 12, 36 and 48 hours; 3) Epidural anesthetic solution consumption; 4) Patient-Controlled Epidural Analgesia (PCEA) administration frequency; 5) Opioid consumption; 6) PONV; 7) Motor block occurrence (Bromage Scale ≥ 1); 8) Pruritus; 9) Hypotension; and 10) Patient satisfaction.

### Data extraction

Two authors (G.R.M.W. and A.M.M.) independently extracted data for each study using a standardized study form to determine: authors, study publication year, inclusion and exclusion criteria, sample size, follow-up period, baseline patient characteristics, type of surgery, intraoperative anesthesia, epidural analgesia characteristics, and adjuvant rescue analgesia techniques. Outcomes of interest were independently extracted by two authors (G.L.G. and B.F.M.W.). Any discordant assessments were adjudicated by the senior investigator (T.S.N.).

When data were reported as median with Interquartile Range (IQR) or median with range, we estimated the mean and Standard Deviation (SD) using the method developed by Luo, Shi, and Wan.^17^, ^18^, ^19^ When available, outcome data derived from intention-to-treat analyses were preferentially extracted. When trials reported outcomes using per-protocol or as-treated analyses, data were extracted as reported. No attempt was made to reconstruct intention-to-treat analyses or to reassign participants to randomized groups when such analyses were not reported.

All extracted numerical pain scores reported on 0–100 scales were converted to 0‒10, and patient satisfaction scores originally reported on 0–10 scales were converted to 0‒100 scales. Brief Pain Inventory reporting “pain in the last 24 hours” was considered equivalent to “pain at 24 hours”.

Data on the “total epidural volume” outcome were converted from milligrams to milliliters, if necessary, due to available solution concentration. Likewise, data related to PCEA administrations were converted from volume (mL) to the number of administrations when the bolus volume per administration was provided. Data on rescue opioid analgesia were converted to “Oral Morphine Milligrams Equivalent” (OMME).^20^

Whenever feasible, heterogeneous outcome definitions (pain, hypotension, and PONV) were standardized to ensure consistency across studies. Detailed descriptions of outcome harmonization and study-specific adjustments are provided in Supplemental Table S3. Except for pain scores, all outcomes were analyzed as cumulative measures from the start of the postoperative period, ensuring no double-counting across time intervals. The follow-up duration applied to each outcome is detailed in Supplemental Table S4.

### Subgroup and sensitivity analysis

Subgroup analyses were conducted to explore potential sources of heterogeneity and to assess the influence of clinical and methodological factors on pain outcomes and adverse events. Analyses were stratified by obstetric versus non-obstetric setting to account for physiological influences of labor. Additional subgroup analyses evaluated the impact of higher flow rates (defined as ≥ 5 mL.h^−1^) and study Risk of Bias (RoB), comparing trials with increased risk to those at low risk according to RoB-2 assessment.

For the primary outcome, further subgroup analyses were performed according to surgical site, epidural solution, and PIEB interval. Secondary outcomes with shorter follow-up durations were stratified according to studies reporting outcomes at ≥ 24 vs. < 24h.

Sensitivity analyses were performed for rest pain at 24 hours to assess the robustness of the findings. These included leave-one-out analyses and subgroup analyses separating studies with skewed data from those assuming normal distributions.

### Quality assessment and risk of bias

**Figure 1:**
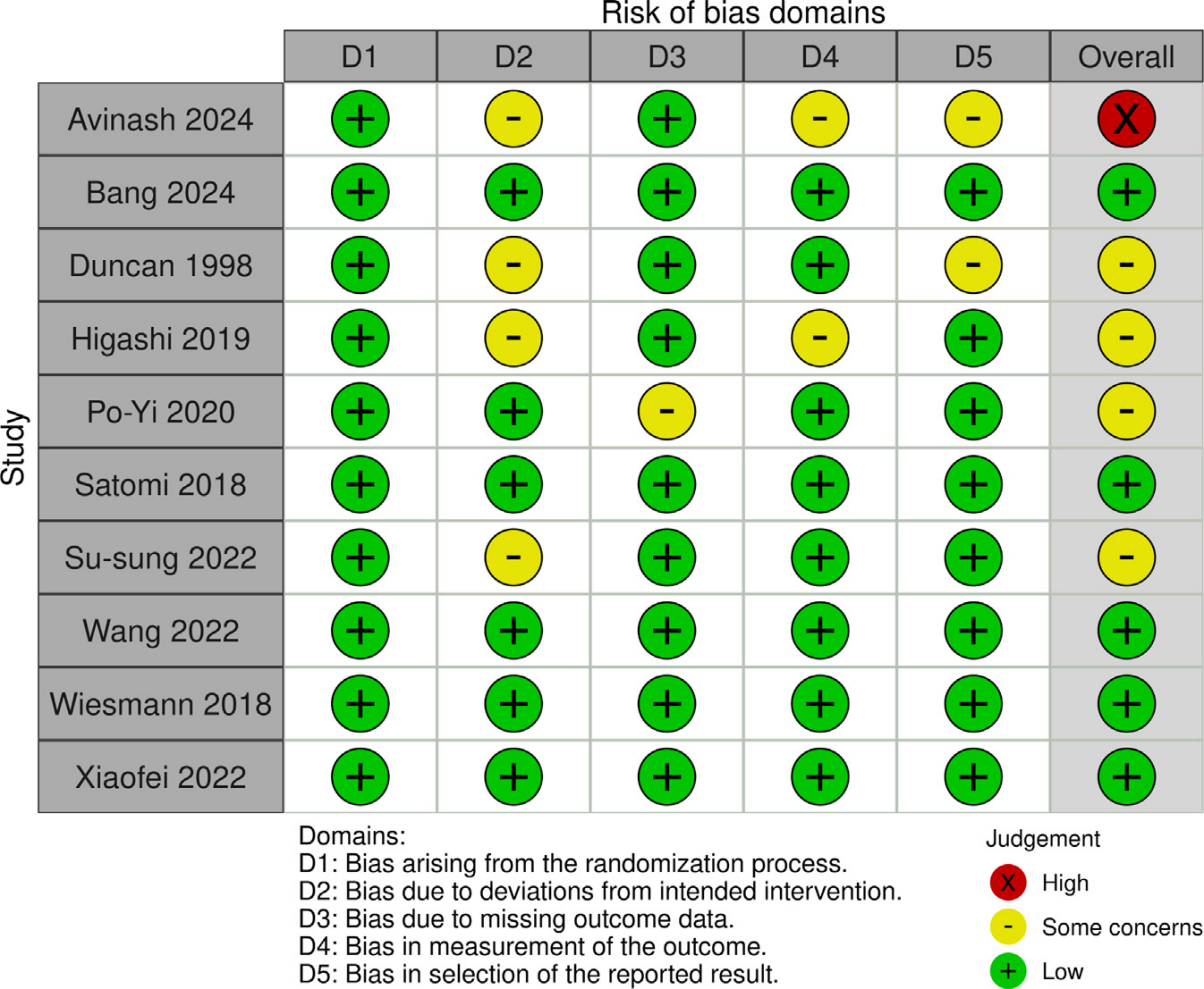
**Risk of Bias.** Graphic representation of the RoB-2 risk of bias assessment informing the risk of every study’s individual domain.

Risk of bias and quality assessment of individual studies were analyzed using the Cochrane Collaboration’s tool RoB-2 for assessing the risk of bias in randomized studies and displayed using Robvis.^21^^,^^22^ Each trial was rated as “low risk,” “some concerns”, or “high risk” across five domains: randomization process, deviations from intended interventions, missing outcome data, measurement of the outcome, and selection of the reported result. Assessments were conducted with respect to the effect of assignment to the intervention, consistent with an intention-to-treat framework. This assessment was performed independently by two authors (G.L.G. and G.F.M.W.), with disagreements resolved by consensus discussion involving the senior author (T.S.N.) (Fig. 1).

Potential reporting bias was assessed by visual inspection of contour-enhanced funnel plots for all outcomes. Effect estimates were plotted against their standard errors, with contours representing conventional significance thresholds and dashed pseudo-confidence limits centered on the pooled random-effects estimate. Egger’s regression was planned for outcomes including at least 10 RCTs but was not performed because no outcome met this threshold.

Grading of Recommendations Assessment, Development, and Evaluation (GRADE).^23^ was also performed by two authors (G.L.G. and I.L.L.M.) on the most relevant clinical outcomes: rest pain at 24 hours; movement-related pain at 24 hours; opioid rescue analgesia consumption; total epidural volume; occurrence of hypotension; PONV; and patient satisfaction.

### Statistical analysis

Binary outcomes were summarized as Risk Ratios (RRs), while continuous outcomes were analyzed using Mean Differences (MDs). Numerical rating scales, including the Visual Analog Scale (VAS) and the Numerical Rating Scale (NRS), were pooled using MDs due to their conceptual similarity and comparable clinical interpretability. For studies reporting medians without corresponding measures of dispersion, standard deviations were not imputed to minimize the risk of introducing bias.

Effect estimates are reported with 95% Confidence Intervals (95% CIs) and 95% Prediction Intervals (95% PIs). For continuous outcomes, MDs were calculated as PIEB minus CEI. For outcomes where lower values reflect benefit (e.g., pain scores and anesthetic consumption), negative MDs favor PIEB. For binary outcomes, RRs greater than 1 indicate a higher risk with PIEB, whereas values less than 1 favor PIEB.

Statistical heterogeneity was evaluated using Cochran’s *Q*-test and the *I*^2^ statistic. A p-value below 0.10 for the *Q*-test was considered indicative of statistically significant heterogeneity. *I*^2^ values of < 25%, 25–50%, and > 50% were interpreted as low, moderate, and high heterogeneity, respectively. Owing to anticipated clinical variability across studies, a random-effects model was applied to all outcomes, with between-study variance estimated using the Restricted Maximum Likelihood (REML) method. No additional small-sample correction was applied, and statistical inference relied on conventional Wald-type confidence intervals. Statistical significance was defined as p < 0.05. All analyses were performed using R software (version 4.5.0; R Foundation for Statistical Computing, Vienna, Austria).

## Results

### Study selection and characteristics

**Figure 2:**
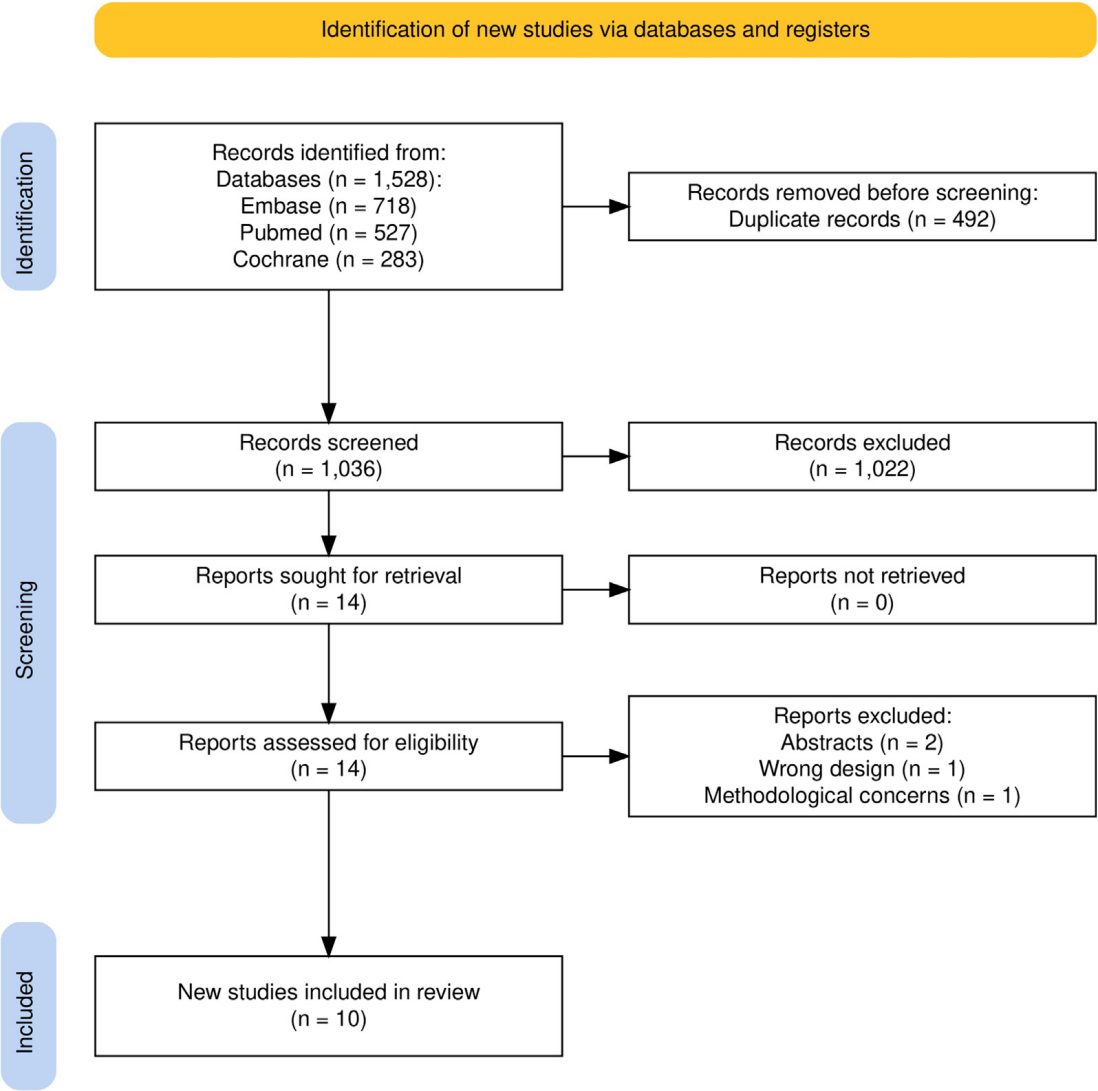
**Prisma Flow Diagram.** Diagram depicting information pertaining to different steps of the systematic review search process. PRISMA, Preferred Reporting Items for Systematic Reviews and Meta-Analysis.

Our initial search yielded 1528 potential articles. After removing duplicates and screening by title and abstract, 14 articles were retrieved and thoroughly reviewed for eligibility. Finally, 10 RCTs.^24^, ^25^, ^26^, ^27^, ^28^, ^29^, ^30^, ^31^, ^32^, ^33^ met the inclusion criteria and were included in the analysis. A total of 692 patients were included, with a mean age ranging from 31.6 to 65 years, and 17.7% of participants were male. A comprehensive description of included studies is provided in Table 1, as well as a description of excluded studies in Supplemental Table S5. The complete study selection process is detailed in the flow diagram presented in Figure 2.

**Table 1:** Comprehensive description of included studies.

Author	n	Male (%)	Age (years)	ASA	Surgery	Type of anesthesia	Epidural Catheter	Epidural Solution	Loading dose	CEI	PIEB	Rescue analgesia	Follow-up
Avinash 2024	30	55%	60.3 [7.95]	I-II	Lower limb	Spinal	L2-3; L3-4	Ropivacaine 0.1% + Morphine 1 mg	-	6 mL.h^−1^	6 mL/ 60 min	PCEA: 4 mL; LT: 30 min; IV tramadol 50 mg	24 hours
Bang 2024	74	0%	35.3 [3.38]	II	Cesarean	Spinal	L2-3	Ropivacaine 0.11% + Fentanyl 3.7 μg.mL^−1^	-	4 mL.h^−1^	4 mL/ 60 min	PCEA: 2 mL; LT: 15 min; MV: 12 mL; IM Ketoprofen 100 mg	36 hours
Duncan 1998	40	0%	46.4 [14.83]	I-III	Lower abdominal	GA	T10–11; T11-12; T12–L1; L2–3	Bupivacaine 0.375%	Intraoperative or at end of surgery: 10 mL of bupivacaine 0.5%	5 mL.h^−1^	5 mL/ 60 min	PCA: morphine 1 mg; LT: 5 min	24 hours
Higashi 2019	42	-	65 [3.34]	-	Open lung lobectomy or partial lobectomy	GA	T4-6	Ropivacaine 0.2% + Fentanyl 2 μg.mL^−1^	Postoperative: 5 mL of ropivacaine 0.2% + Fentanyl 2 μg.mL^−1^	3.4 mL.h^−1^	5.1 mL/ 90 min	PCEA: 3 mL; LT: 15 min	36 hours
Po-Yi 2020	120	29%	56.5 [15.04]	I-III	Major abdominal	GA	T6-8; T9-10; T11-12	Ropivacaine 0.0625% + Fentanyl 2 μg.mL^−1^	Postoperative: 5 mL ropivacaine 0.0625% + Fentanyl 2 μg.mL^−1^	8 mL.h^−1^	4 mL/ 30 min	PCEA - CEI 2 mL; LT: 15 min PCEA - PIEB 2 mL; LT: 10 min; Opioids	72 hours
Satomi 2018	54	0%	53.5 [11.42]	I-III	Gynecological	GEA	T10-11; T11-12	Ropivacaine 0.2% + Fentanyl 2 μg.mL^−1^	At induction: 6 mL of ropivacaine 0.2% + Fentanyl 1.6 mcg.mL^−1^	4 mL.h^−1^ *	4 mL/ 60 min**	PCEA: 4 mL/h; LT: 1 hour. MV: 12 mL/h	48 hours
Su-sung 2022	70	68%	63.1 [9.11]	I-III	Open thoracotomy	GA	T6-7; T7-8	Levobupivacaine 0.2% + Fentanyl 3.3 μg.mL^−1^	At induction: 7.5 mL of levobupivacaine 0.2% + Fentanyl 6.67 mcg.mL^−1^	1.1 mL.h^−1^	3 mL/ 180 min	PCEA: 3 mL; LT: 30 min; Ketorolac 30 mg	48 hours
Wang 2022	58	0%	31.6 [4.29]	I-II	Cesarean	Spinal	L1-2	Ropivacaine 0.2% + Fentanyl 2 μg.mL^−1^	Postoperative: 6 mL of ROPI 0.2% + Fentanyl 2 μg.mL^−1^	3 mL.h^−1^*	3 mL/ 60 min**	PCEA: 2 mL; LT: 15 min; MV: 12 mL	48 hours
Wiesmann 2018	84	28%	60.6 [14.18]	I-III	Major abdominal & gynecological cancer	GEA	T8-9; T9-10	Ropivacaine 0.2% + Sufentanil 0.75 μg.mL^−1^	At induction: 15 mL of ropivacaine 0.375%	6 mL.h^−1^	6 mL/ 60 min	PCEA: 4 mL; LT: 30 min	48 hours
Xiaofei 2022	120	0%	33 [4.97]	II-III	Cesarean	CSE	L2-3; L3-4	Ropivacaine 0.1%	Postoperative: 8 mL of ropivacaine 0.125% + hydromorphone 0.6 mg + naloxone 0.04 mg	6 mL.h^−1^	6 mL/ 60 min ***	PCEA: 6 mL; LT: 15 min. MV: 24 mL; Rectal diclofenac 100 mg	36 hours

Age expressed in Mean [SD]; ASA, American Society of Anesthesiologists Physical Status; CEI, Continuous epidural infusion; PIEB, Programmed Intermittent Epidural Bolus; GA, General anesthesia; PCA, Patient controlled analgesia; GEA, Combined general-epidural anesthesia; PCEA, Patient controlled epidural analgesia; LT, lockout time; MV, Maximum volume; CSE, Combined spinal-epidural; IV, Intravenous.

⁎ Started immediately after loading dose;

⁎⁎ Started 1 hour after loading dose;

⁎⁎⁎ Started 30 minutes after loading dose.

### Outcomes

#### Primary outcomes

**Figure 3:**
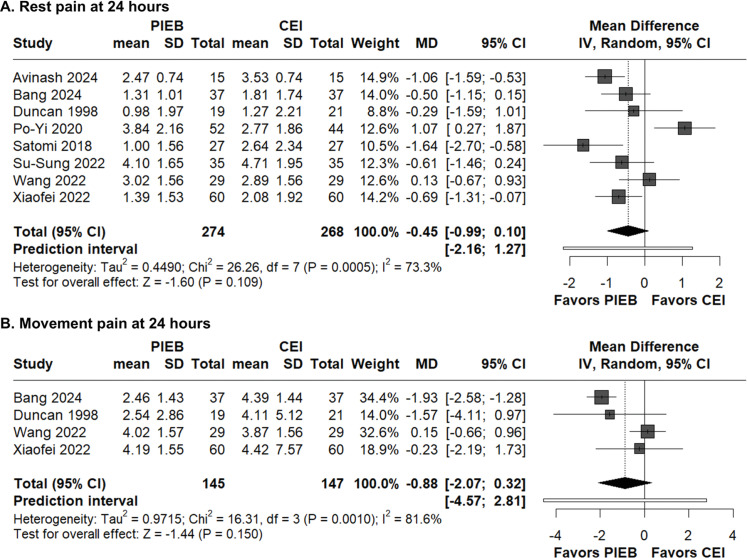
**Pain at 24 hours.** Forest plots comparing PIEB and CEI (both with PCEA) on postoperative rest and movement-related pain (defined as pain elicited by movement), in a scale ranging from 0 to 10, at 24 hours. CEI, Continuous Epidural Infusion; PCEA, Patient-Controlled Epidural Analgesia; PIEB, Programmed Intermittent Epidural Bolus.

The PIEB group showed no statistically significant difference in rest pain at 24 hours (MD = -0.45; 95% CI -0.99 to 0.10; 95% PI = -2.16 to 1.27; p = 0.109; *I*^2^ = 73.3%; Fig. 3A) or movement-related pain scores at 24 hours (MD = -0.88; 95% CI -2.07 to 0.32; 95% PI -4.57 to 2.81; p = 0.15; *I*^2^ = 81.6%; Fig. 3B).

#### Secondary outcomes

**Figure 4:**
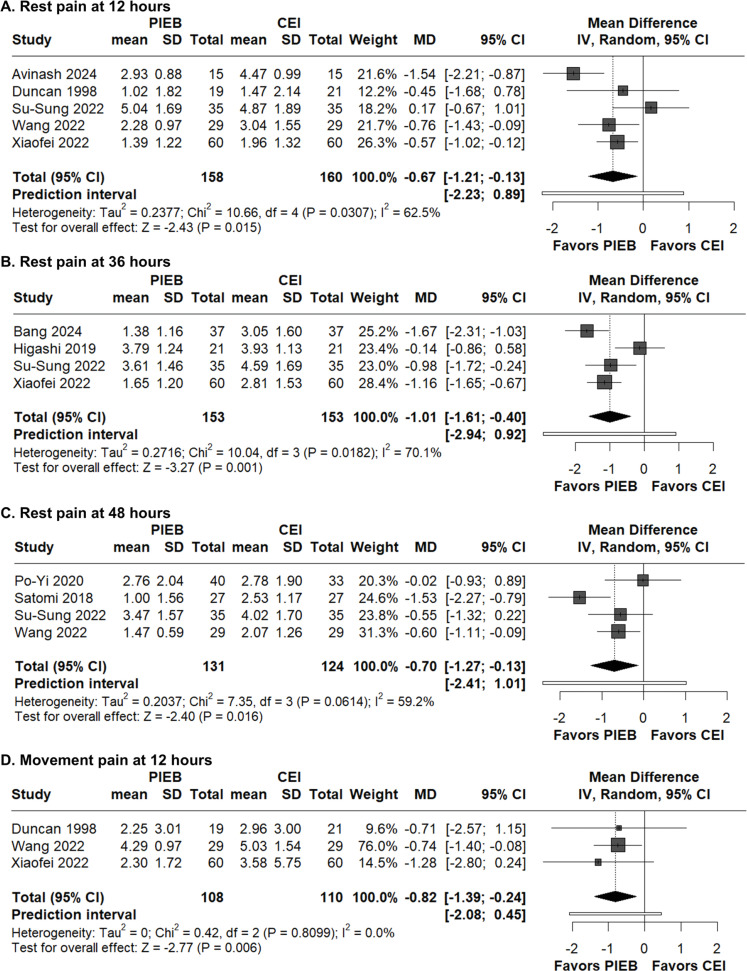
**Pain at other intervals.** Forest plots comparing PIEB and CEI (both with PCEA) on postoperative rest pain at 12 (A), 36 (B) and 48 hours (C), as well as movement-related pain (defined as pain elicited by movement) at 12 hours (D), both in a scale ranging from 0 to 10. CEI, Continuous Epidural Infusion; PCEA, Patient-Controlled Epidural Analgesia; PIEB, Programmed Intermittent Epidural Bolus.

Rest pain was significantly lower with PIEB at 12 hours (MD = -0.67; 95% CI -1.21 to -0.13; 95% PI -2.23 to 0.89; p = 0.015; *I*^2^ = 62.5%; Fig. 4A), at 36 hours (MD = -1.01; 95% CI -1.61 to -0.40; 95% PI -2.94 to 0.92; p = 0.001; *I*^2^ = 70.1%; Fig. 4B), and at 48 hours (MD = -0.70; 95% CI -1.27 to -0.13; 95% PI -2.41 to 1.01; p = 0.016; *I*^2^ = 59.2%; Fig. 4C). Meanwhile, movement-related pain was lower at 12 hours (MD = -0.82; 95% CI -1.39 to -0.24; 95% PI -2.08 to 0.45; p = 0.006; *I*^2^ = 0.0%; Fig. 4D), but not at 36 hours (Fig. S1). Insufficient data precluded the analysis of movement-related pain at 48 hours.

**Figure 5:**
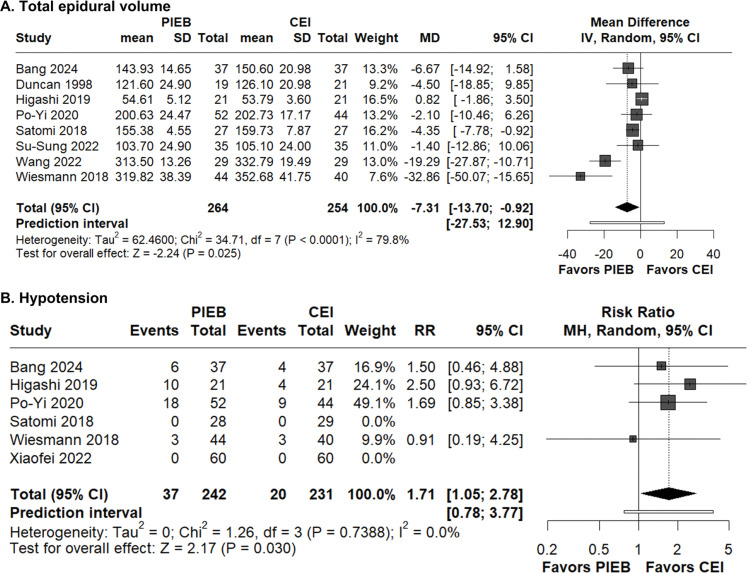
**Total epidural volume and hypotension.** Forest plots comparing PIEB and CEI (both with PCEA) on total epidural volume infused (in milliliters) (A) and the occurrence of postoperative hypotension (B). CEI, Continuous Epidural Infusion; PCEA, Patient-Controlled Epidural Analgesia; PIEB, Programmed Intermittent Epidural Bolus.

Regarding indirect pain-related outcomes, PIEB was associated with a lower total epidural volume administered (MD = -7.31 mL; 95% CI -13.7 to -0.92 mL; 95% PI -27.53 to 12.90 mL; p = 0.025; *I*^2^ = 79.8%; Fig. 5A). For binary secondary outcomes, PIEB was associated with higher incidence of hypotension (RR = 1.71; 95% CI 1.05 to 2.78; 95% PI 0.78 to 3.77; p = 0.03; *I*^2^ = 0.0%; Fig. 5B).

Finally, no statistically significant differences were found between groups in PCEA administrations (Fig. S2), incidence of pruritus, urinary retention, PONV, motor block, or paresthesia (Fig. S3).

### Subgroups and sensitivity analysis

Subgroup analyses by obstetric setting showed that cesarean patients receiving PIEB had lower movement-related pain at 36 hours (MD = -2.11; 95% CI -3.00 to -1.22; 95% PI -9.18 to 4.95; *I*^2^ = 12.4%) and higher satisfaction (MD = 8.59; 95% CI 1.89 to 15.28; 95% PI -56.04 to 73.22; *I*^2^ = 57.6%), with reduced heterogeneity and narrower 95% CIs for rest pain at 12 and 36 hours. Higher flow rates were associated with lower rest pain at 24 hours and a narrower 95% PI (MD = -0.85; 95% CI -1.23 to -0.47; 95% PI -1.69 to -0.01; *I*^2^ = 0.0%).

Studies at low RoB favored PIEB for satisfaction (MD = 8.59; 95% CI 1.89 to 15.28; 95% PI -56.04 to 73.22; *I*^2^ = 57.6%) and movement-related pain at 36 hours (MD = -2.11; 95% CI -3.00 to -1.22; 95% PI -9.18 to 4.95; *I*^2^ = 12.4%). Unlike studies with increased RoB, those with low-RoB also demonstrated statistically significant differences favoring PIEB for rest pain at 12, 36, and 48 hours, movement-related pain at 12 hours, and total epidural volume.

Further directed subgroup analyses for rest pain at 24 hours showed significant effects favoring PIEB in epidural solutions containing only local anesthetics (MD = -0.62; 95% CI -1.18 to -0.06; 95% PI -4.25 to 3.02; *I*^2^ = 0.0%), PIEB intervals of 60-min (MD = -0.68; 95% CI -1.11 to -0.25; 95% PI -1.77 to 0.40; *I*^2^ = 48.2%), and studies without skewed data (MD = -0.75; 95% CI -1.24 to -0.26; 95% PI -2.08 to 0.58; *I*^2^ = 54.4%). No surgical site subgroup favored either intervention. Movement-related pain at 24 hours was not subjected to subgroup analysis because of the limited number of studies.

Stratification of secondary outcomes by follow-up duration did not materially alter results; however, hypotension lost statistical significance in both < 24h and ≥ 24h subgroups. All subgroup analyses were exploratory and hypothesis-generating and should be interpreted cautiously given multiple testing and limited sample sizes (Supplemental Figs. S4–S9).

Leave-one-out sensitivity analyses reduced heterogeneity and favored PIEB for rest pain at 24 hours after exclusion of Po-Yi et al.^28^ (MD = -0.68; 95% CI -1.04 to -0.32; *I*^2^ = 38.1%; Fig. S7) and for movement-related pain at 24 hours after exclusion of Wang et al.^32^ (MD = -1.51; 95% CI -2.56 to -0.47; *I*^2^ = 24.0%). Exclusion of Bang et al.^33^ eliminated heterogeneity without altering statistical significance. Leave-one-out sensitivity analyses are presented in Supplemental Figures S10.

### Risk of Bias

Based on the RoB-2 assessment, five of the ten included studies had some concerns, mainly related to missing outcome data and loss to follow-up, and one study^27^ was judged at high risk of bias due to multiple domain-level concerns (Fig. 2).

Contour-enhanced funnel plots did not show clear evidence of reporting bias; studies were generally symmetrically distributed around pooled effects, with any observed asymmetry occurring within statistically significant contours and not altering the direction of effect. Interpretation was limited by imprecision and the small number of contributing trials, and no outcome included ≥ 10 RCTs to permit Egger’s regression (Fig. S11).

### Quality assessment

In the GRADE assessment, rest pain at 24 hours, total epidural volume, hypotension, and PONV were considered to have low certainty of evidence due to the relevant heterogeneity and high study variability. Meanwhile, movement-related pain at 24 hours, opioid rescue analgesia, and patient satisfaction were considered to have a very low certainty of evidence, as they also presented few events or studies. The evidence profile and summary of findings are presented in Supplemental Tables S6–S7.

## Discussion

This systematic review and meta-analysis of 10 RCTs, encompassing 692 patients, compared the clinical outcomes of PIEB versus CEI, both combined with PCEA, for postoperative analgesia. The primary analysis revealed no statistically significant difference in pain scores at 24 hours, a key time point in postoperative recovery. Moreover, although PIEB was associated with mildly lower rest pain at other time points (12, 36, and 48 hours) and reduced total anesthetic volumes, these benefits were offset by a higher incidence of hypotension. No significant differences were observed in rescue opioid consumption, PCEA demands, patient satisfaction, or other adverse effects, including motor block and PONV.

The superior analgesic effects attributed to PIEB in labor analgesia^10^, ^11^, ^12^^,^^34^ have been linked to a wider epidural spread from high-pressure intermittent boluses, a mechanism supported by experimental studies.^35^, ^36^, ^37^, ^38^ This broader spread may enhance dermatomal coverage and analgesic efficiency.^39^, ^40^, ^41^ However, such a physiological advantage likely depends on relatively homogeneous pain patterns in labor, whereas postoperative pain is more heterogeneous, involving variable somatic, visceral, and neuropathic components across surgical contexts. This variability, particularly when combined with PCEA-mediated bolus dosing, may attenuate any incremental benefit of programmed boluses.

Although PIEB was associated with statistically significant pain reductions at some timepoints, most effects did not reach the Minimal Clinically Important Difference (MCID) of one point on a 0–10 scale.^42^ Instances exceeding the MCID, such as rest pain at 36 hours in mixed cohorts and movement-related pain at 36 hours in cesarean patients, or where the 95% CI exceeded the MCID, in movement-related pain at 36 hours in low RoB studies, were supported by limited data. Thus, these isolated findings should be interpreted cautiously and in the context of adjacent timepoints that did not demonstrate clinically meaningful differences.

Furthermore, the combination of a non-significant pooled effect with substantial heterogeneity and wide PIs in the primary outcome suggests clinically meaningful variation across settings, indicating that the pooled estimates may not adequately represent individual surgical contexts. Variations in surgical pain intensity, catheter tip position, and solution viscosity likely influenced epidural spread, contributing to heterogeneity and attenuating the relative impact of the epidural component. Consistently, the absence of clinically relevant differences in pain scores, opioid consumption, and PCEA use challenges the hypothesis that PIEB provides superior analgesic efficacy over CEI when both are combined with PCEA. It is plausible that the bolus mechanism inherent to PCEA, together with concurrent multimodal analgesia, further diminishes any incremental benefit of programmed boluses.

Exploratory subgroup and sensitivity analyses demonstrated only modest variations in effect estimates when evaluating the potential influence of labor-related physiological changes in postoperative settings ‒ through analyses restricted to patients undergoing cesarean delivery ‒ as well as when stratifying by epidural flow rate, RoB, PIEB interval, surgical site, anesthetic solution, data skewness, and follow-up duration. Despite isolated subgroup differences and multiple circumstances, the overall findings remained consistent with the primary analysis, showing no uniform clinically meaningful reduction in pain scores and a persistently increased risk of hypotension associated with PIEB.

Moreover, the overall consistency observed across subgroup and sensitivity analyses ‒ despite several scenarios in which heterogeneity was reduced, such as analyses limited to cesarean delivery, studies at low RoB, and protocols using local anesthetics alone ‒ suggests that, although residual confounding related to the inclusion of mixed surgical settings cannot be excluded, the observed effects are likely reflective of the underlying clinical comparison of interest. Given the number of subgroup analyses performed, these findings are subject to an increased risk of false-positive results and should be interpreted cautiously.

Furthermore, exclusion of Po-Yi et al.,^28^ which uniquely reported pain as “average pain”, or Wang et al.,^32^ which applied an unclear definition of movement-related pain, shifted pooled estimates in favor of PIEB and markedly reduced heterogeneity. While these findings may indicate outcome-definition-related bias in individual studies, they more importantly underscore the fragility of the available evidence and support the overall conclusion that clinical significance remains uncertain, despite the presence of isolated statistically significant results.

A key finding of this analysis is the increased risk of hypotension associated with PIEB, with an absolute risk increase of approximately 6.2% (range 0.4%–15.4%), a signal inconsistently reported in prior meta-analyses.^10^^,^^11^ This effect is biologically plausible, reflecting broader sympathetic blockade from enhanced epidural spread and reduced vascular tone.^35^ Follow-up-stratified subgroup analyses (< 24 lt; 24 vs. ≥ 24 hours), performed to explore potential temporal attenuation of hypotension as PCEA requirements decreased, were limited by reduced statistical power and did not reach statistical significance. Given that postoperative hypotension is an independent risk factor for major complications, including myocardial injury and acute kidney injury,^43^^,^^44^ any potential analgesic benefit of PIEB should be weighed against this hemodynamic risk.

These findings should be interpreted within the evolving landscape of postoperative pain management. The role of epidural analgesia itself is progressively being re-evaluated against newer regional techniques,^5^^,^^6^ which may carry a lower risk of failure, complications, and contraindications.^7^^,^^8^^,^^45^ The choice between PIEB and CEI represents refinement within a single technique, and clinicians should also evaluate less invasive alternatives according to patient and surgical factors.

Our analyses indicate that, contrary to theoretical expectations and previous findings in other settings, PIEB does not provide a significant analgesic advantage over CEI when both are combined with PCEA. Clinicians should consider these findings when selecting an epidural modality, balancing PIEB’s inconsistent analgesic benefits against its potential for hemodynamic instability. Further high-quality RCTs with standardized protocols and clearly defined outcomes are needed to clarify the role of PIEB in specific surgical populations.

This meta-analysis has several limitations. Considerable heterogeneity existed across trials in surgical procedures, epidural techniques, bolus volumes and intervals, and follow-up duration. The predominance of female participants ‒ largely reflecting gynecologic and obstetric surgery ‒ limits generalizability to male patients. Outcome definitions and assessment time points varied, and several studies reported incomplete or imprecise data. In addition, the small number of trials per outcome precluded formal assessment of small-study effects. To address these issues, we standardized outcomes when feasible, performed prespecified subgroup and sensitivity analyses, and qualitatively evaluated funnel plot asymmetry.

## Conclusion

Our findings indicate that PIEB does not provide a clinically meaningful or consistent analgesic advantage over CEI when both are combined with PCEA for postoperative analgesia. However, definitive inferences are limited by substantial heterogeneity across studies. In contrast, PIEB is associated with an increased risk of hypotension, representing a clinically relevant adverse effect that should be considered when selecting an epidural maintenance strategy.

## Data availability statement

The dataset and R code used to reproduce all analyses are publicly available at Zenodo (DOI: https://doi.org/10.5281/zenodo.20102829). GRADE assessments were performed using the GRADEpro GDT online tool (gradepro.org), and risk-of-bias visualizations were generated using the Robvis web application; these outputs are not reproduced by the provided R script.

## AI assistance disclosure

The AI tool “Manus AI” was used exclusively for language polishing. All outputs were reviewed and edited by the authors. No AI or automated tool was involved in the core intellectual processes of the research. The authors assume full responsibility for the content and originality of this work.

## Authors’ contributions

Conception and design of the research: G.L.G. Acquisition of data: G.L.G., V.F.S., I.L.L.M., and B.F.M.W. Analysis and interpretation of the data: G.L.G., B.F.M.W., G.R.M.W., and A.M.M. Quality assessment: G.L.G., G.R.M.W., and I.L.L.M. Writing of the manuscript: All authors; Critical revision of the manuscript for intellectual content: All authors. Project supervision: G.L.G. and T.S.N.

## Funding

This research did not receive any specific grant from funding agencies in the public, commercial, or not-for-profit sectors.

## Conflicts of interest

The authors declare no conflicts of interest.

## Appendix Supplementary materials

Image, application 1

## Footnotes

Supplementary material associated with this article can be found in the online version at doi:10.1016/j.bjane.2026.844766.
