# Supplementary material for: Programmed intermittent epidural bolus versus continuous epidural infusion combined with patient-controlled epidural analgesia for postoperative analgesia: a meta-analysis of randomized controlled trials
Source: Braz J Anesthesiol. 2026 May 23;76(4):844766. doi: 10.1016/j.bjane.2026.844766 (PMC13315847; doi:10.1016/j.bjane.2026.844766)
Supplement: Supplementary file 1 [file mmc1.docx]

BJAN-D-25-00729

**SUPPLEMENTARY APPENDIX**

### **Programmed intermittent epidural bolus versus continuous epidural infusion combined with patient-controlled epidural analgesia for postoperative analgesia: a meta-analysis of randomized controlled trials**

**Gabriel Lemos González^a^, Vinícius Fernandes Sarmento^b^, Bruno Francisco Minetto Wegner^c^, Gustavo Roberto Minetto Wegner^d^, Alesson Marinho Miranda^e^, Iaci Luisa Lopes de Mattos^a^, Tatiana Souza do Nascimento^f,^***

^a^ Universidade Federal do Estado do Rio de Janeiro (UNIRIO), Rio de Janeiro, RJ, Brazil

^b^ Hospital do Servidor Público Estadual de São Paulo, São Paulo, SP, Brazil

^c^ Universidade Federal do Rio Grande do Sul, Porto Alegre, RS, Brazil

^d^ Universidade Federal da Fronteira Sul, Chapecó, SC, Brazil

^e^ Universidade Federal de São Paulo, São Paulo, SP, Brazil

^f^ University of Iowa, Iowa, United States of America

**Table of Contents**

**Supplemental Table S1. PRISMA 2020 main checklist……………………….…...….3**

**Supplemental Table S2. Details of search strategy…………………...………....……5**

**Supplemental Table S3 Heterogeneous outcome availability and interpretation..6**

**Supplemental Table S4. Follow-up duration of each outcome………..……….…...7**

**Supplemental Table S5. Excluded studies and reasons for exclusion…………...8**

**Supplemental Table S6. GRADE Evidence Profile………………………………….…9**

**Supplemental Table S7. GRADE Summary of Findings………………………………10**

**Supplemental Figure S1. Movement-related pain at 36 hours………………..…....11**

**Supplemental Figure S2. Remaining continuous secondary outcomes..............12**

**Supplemental Figure S3. Remaining binary secondary outcomes…….………....13**

**Supplemental Figure S4. Subgroup analyses: obstetric or non-obstetric setting……………………………………………………………………………………….15**

**Supplemental Figure S5. Subgroup analyses: epidural infusion flow rate………23**

**Supplemental Figure S6. Subgroup analyses: risk of bias stratification………..31**

**Supplemental Figure S7. Rest pain at 24 hours: other subgroup analyses……..39**

**Supplemental Figure S8. Rest pain at 24 hours: skewed data subgroup………..41**

**Supplemental Figure S9. Secondary outcome subgroup analysis………….……42**

**Supplemental Figure S10. Leave-one-out sensitivity analysis…………….……...45**

**Supplemental Figure S11. Funnel plots………………………….…………….……...46**

**Supplemental Table S1. PRISMA 2020 main checklist**

| **Section and Topic** | **Item#** | **Checklist item** | **Location where items is reported** |
| --- | --- | --- | --- |
| **TITLE** | | |  |
| Title | 1 | Identify the report as a systematic review. | Pg 1 at MS |
| **ABSTRACT** | | |  |
| Abstract | 2 | See the PRISMA 2020 for Abstracts checklist. | Pg 1 at MS |
| **INTRODUCTION** | | |  |
| Rationale | 3 | Describe the rationale for the review in the context of existing knowledge. | Pg 2 at MS |
| Objectives | 4 | Provide an explicit statement of the objective(s) or question(s) the review addresses. | Pg 3 at MS |
| **METHODS** | | |  |
| Eligibility criteria | 5 | Specify the inclusion and exclusion criteria for the review and how studies were grouped for the syntheses. | Pg 4 at MS |
| Information sources | 6 | Specify all databases, registers, websites, organizations, reference lists and other sources searched or consulted to identify studies. Specify the date when each source was last searched or consulted. | Pg 4 at MS |
| Search strategy | 7 | Present the full search strategies for all databases, registers and websites, including any filters and limits used. | Table S2 |
| Selection process | 8 | Specify the methods used to decide whether a study met the inclusion criteria of the review, including how many reviewers screened each record and each report retrieved, whether they worked independently, and if applicable, details of automation tools used in the process. | Pg 4-5 at MS |
| Data collection process | 9 | Specify the methods used to collect data from reports, including how many reviewers collected data from each report, whether they worked independently, any processes for obtaining or confirming data from study investigators, and if applicable, details of automation tools used in the process. | Pg 4-5 at MS |
| Data items | 10a | List and define all outcomes for which data were sought. Specify whether all results that were compatible with each outcome domain in each study were sought (e.g. for all measures, time points, analyses), and if not, the methods used to decide which results to collect. | Pg 5 at MS |
|  | 10b | List and define all other variables for which data were sought (e.g. participant and intervention characteristics, funding sources). Describe any assumptions made about any missing or unclear information. | Pg 5 at MS and Table 1 and S3 |
| Study risk of bias assessment | 11 | Specify the methods used to assess risk of bias in the included studies, including details of the tool(s) used, how many reviewers assessed each study and whether they worked independently, and if applicable, details of automation tools used in the process. | Pg 7 at MS, Figure 2, Tables S3 and S7 |
| Effect measures | 12 | Specify for each outcome the effect measure(s) (e.g. risk ratio, mean difference) used in the synthesis or presentation of results. | Pg 7-8 at MS |
| Synthesis methods | 13a | Describe the processes used to decide which studies were eligible for each synthesis (e.g. tabulating the study intervention characteristics and comparing against the planned groups for each synthesis (item #5)). | Pg 5-6 at MS, Table S3 |
|  | 13b | Describe any methods required to prepare the data for presentation or synthesis, such as handling of missing summary statistics, or data conversions. | Pg 5-6 at MS |
|  | 13c | Describe any methods used to tabulate or visually display results of individual studies and syntheses. | Pg 7-8 and Table 1 |
|  | 13d | Describe any methods used to synthesize results and provide a rationale for the choice(s). If meta-analysis was performed, describe the model(s), method(s) to identify the presence and extent of statistical heterogeneity, and software package(s) used. | Pg 7-8 at MS |
|  | 13e | Describe any methods used to explore possible causes of heterogeneity among study results (e.g. subgroup analysis, meta-regression). | Pg 6 at MS |
|  | 13f | Describe any sensitivity analyses conducted to assess robustness of the synthesized results. | Pg 6 at MS |
| Reporting bias assessment | 14 | Describe any methods used to assess risk of bias due to missing results in a synthesis (arising from reporting biases). | Pg 7 at MS |
| Certainty assessment | 15 | Describe any methods used to assess certainty (or confidence) in the body of evidence for an outcome. | Pg 7 at MS |
| **RESULTS** | | |  |
| Study selection | 16a | Describe the results of the search and selection process, from the number of records identified in the search to the number of studies included in the review, ideally using a flow diagram. | Pg 9 at MS and Figure 1 |
|  | 16b | Cite studies that might appear to meet the inclusion criteria, but which were excluded, and explain why they were excluded. | Pg 9 at MS, Table S4 |
| Study characteristics | 17 | Cite each included study and present its characteristics. | Table 1 |
| Risk of bias in studies | 18 | Present assessments of risk of bias for each included study. | Figure 2 |
| Results of individual studies | 19 | For all outcomes, present, for each study: (a) summary statistics for each group (where appropriate) and (b) an effect estimates and its precision (e.g. confidence/credible interval), ideally using structured tables or plots. | Pg 9-11 at MS |
| Results of syntheses | 20a | For each synthesis, briefly summarize the characteristics and risk of bias among contributing studies. | Pg 9-11 at MS |
|  | 20b | Present results of all statistical syntheses conducted. If meta-analysis was done, present for each the summary estimate and its precision (e.g. confidence/credible interval) and measures of statistical heterogeneity. If comparing groups, describe the direction of the effect. | Fig 3-5,  Fig S1-S11 |
|  | 20c | Present results of all investigations of possible causes of heterogeneity among study results. | Fig S4-S11 |
|  | 20d | Present results of all sensitivity analyses conducted to assess the robustness of the synthesized results. | Pg 10-11 at MS |
| Reporting biases | 21 | Present assessments of risk of bias due to missing results (arising from reporting biases) for each synthesis assessed. | Pg 11-12 at MS |
| Certainty of evidence | 22 | Present assessments of certainty (or confidence) in the body of evidence for each outcome assessed. | Table S6 and S7 |
| **DISCUSSION** | | |  |
| Discussion | 23a | Provide a general interpretation of the results in the context of other evidence. | Pg 13-14 at MS |
|  | 23b | Discuss any limitations of the evidence included in the review. | Pg 13-16 at MS |
|  | 23c | Discuss any limitations of the review processes used. | Pg 16 at MS |
|  | 23d | Discuss implications of the results for practice, policy, and future research. | Pg 15-16 at MS |
| **OTHER INFORMATION** | | |  |
| Registration and protocol | 24a | Provide registration information for the review, including register name and registration number, or state that the review was not registered. | Pg 4 at MS |
|  | 24b | Indicate where the review protocol can be accessed, or state that a protocol was not prepared. | Pg 4 at MS |
|  | 24c | Describe and explain any amendments to information provided at registration or in the protocol. | NA |
| Support | 25 | Describe sources of financial or non-financial support for the review, and the role of the funders or sponsors in the review. | Pg 16 at MS |
| Competing interests | 26 | Declare any competing interests of review authors. | Pg 16 at MS |
| Availability of data, code and other materials | 27 | Report which of the following are publicly available and where they can be found: template data collection forms; data extracted from included studies; data used for all analyses; analytic code; any other materials used in the review. | Pg 16 at MS |

*Abbreviations: MS, manuscript.*

**Supplemental Table S2. Details of search strategy**

| **Database** | **Search strategy** |
| --- | --- |
| **PubMed** | (surgery OR surgical OR operative OR "Postoperative Period" [mh] OR anesthesia OR "Anesthesia Recovery Period"[mh] OR "Surgical Procedures, Operative"[mh]) AND (continuous OR infusion OR "constant rate" OR maintenance) AND (analgesia OR analgesic OR "pain relief" OR "pain score" OR pain [mh] OR "Analgesics, Opioid/therapeutic use" [mh] OR "Pain Management" [mh]) AND (Epidural OR peridural OR extradural OR "Analgesia, Epidural"[mh]) AND (sequential OR intermittent OR programmed OR PIEB OR pib) |
| **Embase** | (surgery OR surgical OR operative OR ‘Postoperative Period’/exp OR anesthesia OR ‘Anesthesia Recovery Period’/exp OR ‘Surgical Procedures, Operative’/exp) AND (continuous OR infusion OR ‘constant rate’ OR maintenance) AND (analgesia OR analgesic OR ‘pain relief’ OR ‘pain score’ OR pain/exp OR ‘Analgesics, Opioid/therapeutic use’/exp OR ‘Pain Management’/exp) AND (Epidural OR peridural OR extradural OR ‘Analgesia, Epidural’/exp) AND (sequential OR intermittent OR programmed OR PIEB OR pib) |
| **Cochrane Library** | #1 MeSH descriptor: [Postoperative Period] explode all trees  #2 MeSH descriptor: [Anesthesia Recovery Period] explode all trees  #3 MeSH descriptor: [Surgical Procedures, Operative] explode all trees  #4 MeSH descriptor: [Pain] explode all trees  #5 MeSH descriptor: [Analgesics, Opioid] explode all trees  #6 MeSH descriptor: [Pain Management] explode all trees  #7 MeSH descriptor: [Analgesia, Epidural] explode all trees  #8 (surgery OR surgical OR operative OR #1 OR anesthesia OR #2 OR #3) AND (continuous OR infusion OR "constant rate" OR maintenance) AND (analgesia OR analgesic OR "pain relief" OR "pain score" OR #4 OR #5 OR #6) AND (Epidural OR peridural OR extradural OR #7) AND (sequential OR intermittent OR programmed OR PIEB OR pib) |

**Supplemental Table S3. Heterogeneous outcome availability and interpretation**

| **Study** | **Pain scale** | **Pain time** | **Hypotension** | **PONV** | **Satisfaction** |
| --- | --- | --- | --- | --- | --- |
| Avinash 2024 | VAS 0 – 10 | Hours | - | - | - |
| Bang 2024 | NRS 0 – 10 | Hours | ↓20% baseline or  MAP < 65 mmHg | Nausea | NRS 0 – 10 |
| Duncan 1998 | VAS 0 – 100 | Hours | - | - | 4-point scale |
| Higashi 2019 | VAS 0 – 100 | POD | ↓20% baseline;  SBP < 90 mmHg* | PONV | - |
| Po-Yi 2020 | mBPI 0 – 10 | Hours | MAP < 60 mmHg | Nausea | 0 – 100 scale |
| Satomi 2018 | NRS 0 – 10 | Hours | NI | - | - |
| Su-sung 2022 | VAS 0 – 10 | POD | - | Nausea | - |
| Wang 2022 | NRS 0 – 10 | Hours | - | PONV | - |
| Wiesmann 2018 | NRS 0 – 10 | POD | Need for vasopressors | Nausea;  Vomiting* | NRS 0 – 10 |
| Xiaofei 2022 | VAS 0 – 100 | Hours | ↓20% baseline or  SBP < 90 mmHg | Vomiting | VAS 0 – 10 |
| **Interpretation** | | | | | |
| **Pain Scale** | Any continuous numerical scale was accepted. | | | | |
| **Pain time** | POD were converted to “hours” whenever possible:   - Higashi et al: all other outcomes were measured at 36h, we considered POD 1 as a 36h interval. - Wiesmann et al did not specify time of surgery and defined time of evaluation vaguely as “evening”, thus it was excluded from analysis.   Su-sung et al informed surgery time as “the first scheduled in the morning to match the investigation time” and evaluation was performed at “day” from 9-11 AM and at “night” from 9-11 PM, thus POD 0 night, POD 1 day, POD 1 night and POD 2 day were considered approximate to 12, 24, 36, and 48h respectively. | | | | |
| **Hypotension** | Any criteria were accepted.  *Higashi et al presented two separate outcomes for hypotension: “SBP < 90 mmHg” was chosen as a more common criterion | | | | |
| **PONV** | Any criteria were accepted.  *Wiesmann et al presented PONV as two separate outcomes: “nausea” was chosen as a more common criterion. | | | | |
| **Satisfaction** | Any continuous numerical scale was accepted.  Duncan et al. was excluded for presenting a categorical scale. | | | | |

*Supplemental Table S3. Availability and interpretation of outcomes with heterogeneous definitions. VAS: visual analog scale. NRS: numerical rating scale. mBPI: modified brief pain inventory. POD: postoperative day. MAP: mean arterial pressure. SBP: systolic blood pressure. PONV: postoperative nausea and vomiting. NI: not informed.*

**Supplemental Table S4. Follow-up duration of each outcome**

| **Study** | **Opioid consumption** | **PCEA** | **Total epidural volume** | **Satisfaction** | **Pruritus** | **Hypotension** | **Motor block** | **Paresthesia** | **Urinary retention** | **PONV** |
| --- | --- | --- | --- | --- | --- | --- | --- | --- | --- | --- |
| Avinash  2024 | - | - | - | - | - | - | - | - | - | - |
| Bang  2024 | - | 36 hours | 36 hours | 36 hours | 48 hours | 24 hours | 24 hours | 48 hours | 36 hours | 36 hours |
| Duncan  1998 | 4 hours | 24 hours | 24 hours | - | - | - | 24 hours | - | - | - |
| Higashi  2019 | - | 36 hours | 12 hours | - | - | 12 hours | - | - | 36 hours | 36 hours |
| Po-Yi  2020 | 24 hours | 24 hours | 24 hours | 24 hours | 24 hours | 24 hours | - | - | - | 24 hours |
| Satomi  2018 | 48 hours | 48 hours | 40 hours | - | - | 48 hours | 24 hours | - | - | 3 hours |
| Su-sung  2022 | - | 48 hours | 72 hours | - | 48 hours | - | - | 36 hours | 48 hours | 48 hours |
| Wang  2022 | 48 hours | - | 48 hours | - | 24 hours | - | - | 48 hours | - | 48 hours |
| Wiesmann  2018 | 24 hours | 48 hours | 48 hours | 48 hours | - | 12 hours | - | - | - | 12 hours |
| Xiaofei  2022 | - | 36 hours | - | 36 hours | 36 hours | 36 hours | 24 hours | - | 36 hours | 36 hours |

*Supplemental Table S4. Characteristics of follow-up duration of each applicable outcome administrations. PONV: postoperative nausea and vomiting. PCEA: patient-controlled epidural analgesia.*

**Supplemental Table S5. Excluded studies and reasons for exclusion**

| **Excluded study** | **Reason for exclusion** |
| --- | --- |
| Prien (2018) | Abstract only |
| Jinaworn (2024) | Abstract only |
| Komatsu (1998) | Wrong intervention: there was only CEI infusion during nighttime and there was no PIEB group. |
| Kang (2013) | Methodological concerns:  publication as a letter to the editor, no information regarding peer review, and controversial data (no clarification on whether the short data interval represents standard error or standard deviation). |

*Supplemental Table S5. Details on study exclusion. CEI: continuous epidural infusion; PIEB: programmed intermittent epidural bolus.*

**Supplemental Table S6. GRADE Evidence Profile**

| **Certainty assessment** | | | | | | | **№ of patients** | | **Effect** | | **Certainty** | **Importance** |
| --- | --- | --- | --- | --- | --- | --- | --- | --- | --- | --- | --- | --- |
| **№ of studies** | **Study design** | **Risk of bias** | **Inconsistency** | **Indirectness** | **Imprecision** | **Other considerations** | **PIEB** | **CEI** | **Relative (95% CI)** | **Absolute (95% CI)** |  |  |
| **Rest pain at 24 hours (Scale from: 0 to 10)** | | | | | | | | | | | | |
| 8 | randomised trials | not serious | serious^a^ | serious^b^ | not serious | none | 274 | 268 | - | MD **0.45 lower** (0.99 lower to 0.1 higher) | ⨁⨁◯◯ Low^a,b^ | CRITICAL |
| **Movement pain at 24 hours (Scale from: 0 to 10)** | | | | | | | | | | | | |
| 4 | randomised trials | not serious | serious^a^ | serious^b^ | serious^d^ | none | 145 | 147 | - | MD **0.88 lower** (2.07 lower to 0.32 higher) | ⨁◯◯◯ Very low^a,b,d^ | CRITICAL |
| **Opioid rescue analgesia consumption (assessed with: oral morphine milligram equivalent)** | | | | | | | | | | | | |
| 5 | randomised trials | not serious | not serious | serious^b^ | very serious^e^ | none | 171 | 161 | - | MD **0 mg**  (0.4 lower to 0.4 higher) | ⨁◯◯◯ Very low^b,e^ | IMPORTANT |
| **Total epidural volume** | | | | | | | | | | | | |
| 7 | randomised trials | not serious | serious^a^ | serious^b^ | not serious | none | 264 | 254 | - | MD **7.31 mL lower** (13.7 lower to 0.92 lower) | ⨁⨁◯◯ Low^a,b^ | NOT IMPORTANT |
| **Hypotension** | | | | | | | | | | | | |
| 6 | randomised trials | not serious | not serious | Very serious^b,c^ | not serious | none | 37/242 (15.3%) | 20/231 (8.7%) | **RR 1.71** (1.05 to 2.78) | **61 more per 1.000** (from 4 more to 154 more) | ⨁⨁◯◯ Low^b,c^ | IMPORTANT |
| **Postoperative nausea and vomiting (PONV)** | | | | | | | | | | | | |
| 8 | randomised trials | not serious | not serious | Very serious^b,c^ | not serious | none | 81/306 (26.5%) | 73/295 (24.7%) | **RR 1.00** (0.76 to 1.31) | **0 fewer per 1.000** (from 59 fewer to 77 more) | ⨁⨁◯◯ Low^b,c^ | IMPORTANT |
| **Patient satisfaction (Scale from: 0 to 100)** | | | | | | | | | | | | |
| 4 | randomised trials | not serious | serious^a^ | serious^b^ | serious^d^ | none | 193 | 181 | - | MD **3.7 higher** (7.98 lower to 15.39 higher) | ⨁◯◯◯ Very low^a,b,d^ | IMPORTANT |

**CI:** confidence interval; **MD:** mean difference; **RR:** risk ratio; **PIEB:** programmed intermittent epidural bolus; **CEI:** continuous epidural infusion.

#### Explanations

a. High heterogeneity; b. The included studies involved heterogeneous populations and surgical procedures; c. There was variability in the definition of outcomes; d. Analysis contains few studies; e. Possible small-study effect.

**Supplemental Table S7. GRADE Summary of Findings**

|  | | | | | | |
| --- | --- | --- | --- | --- | --- | --- |
| **PIEB analgesia compared to CEI, associated with PCEA, in postoperative analgesia** | | | | | | |
| **Patient or population:** adult patients **Setting:** postoperative period **Intervention:** PIEB with PCEA **Comparison:** CEI with PCEA | | | | | | |
| Outcomes | **Anticipated absolute effects^*^** (95% CI) | | Relative effect (95% CI) | № of participants (studies) | Certainty of the evidence (GRADE) | Comments |
|  | **Risk with CEI** | **Risk with PIEB** |  |  |  |  |
| Rest pain at 24 hours Scale from: 0 to 10 | The mean rest pain at 24 hours ranged from **1.27 to 4.71** | MD **0.45 lower** (0.99 lower to 0.1 higher) | - | 542 (8 RCTs) | ⨁⨁◯◯ Low^a,b^ | The evidence suggests that programmed intermittent epidural analgesia results in little to no difference in rest pain at 24 hours. |
| Movement pain at 24 hours Scale from: 0 to 10 | The mean movement pain at 24 hours ranged from **3.87 to 4.42** | MD **0.88 lower** (2.07 lower to 0.32 higher) | - | 292 (4 RCTs) | ⨁◯◯◯ Very low^a,b,d^ | The evidence is very uncertain about the effect of programmed intermittent epidural analgesia on movement pain at 24 hours. |
| Opioid rescue analgesia consumption assessed with: OMME | The mean opioid rescue analgesia consumption ranged from **0 to 39.68** mg | MD **0 mg**  (0.4 lower to 0.4 higher) | - | 332 (5 RCTs) | ⨁◯◯◯ Very low^b,e^ | The evidence is very uncertain about the effect of programmed intermittent epidural analgesia on opioid rescue analgesia consumption. |
| Total epidural volume | The mean total epidural volume ranged from **53.79 to 352.68** mL | MD **7.31 mL lower** (13.7 lower to 0.92 lower) | - | 518 (7 RCTs) | ⨁⨁◯◯ Low^a,b^ | Programmed intermittent epidural analgesia may result in a slight reduction in total epidural volume. |
| Hypotension | 87 per 1.000 | **148 per 1.000** (91 to 241) | **RR 1.71** (1.05 to 2.78) | 473 (6 RCTs) | ⨁⨁◯◯ Low^b,c^ | Programmed intermittent epidural analgesia may be associated with an increased incidence of hypotension. |
| Postoperative nausea and vomiting (PONV) | 247 per 1.000 | **247 per 1.000** (188 to 324) | **RR 1.00** (0.76 to 1.31) | 601 (8 RCTs) | ⨁⨁◯◯ Low^b,c^ | Programmed intermittent epidural analgesia may not increase/reduce postoperative nausea and vomiting. |
| Patient satisfaction Scale from: 0 to 100 | The mean patient satisfaction ranged from **80** **to 100** | MD **3.7 higher** (7.98 lower to 15.39 higher) | - | 374 (4 RCTs) | ⨁◯◯◯ Very low^a,b,d^ | The evidence is very uncertain about the effect of programmed intermittent epidural analgesia on patient satisfaction. |
| ***The risk in the intervention group** (and its 95% confidence interval) is based on the assumed risk in the comparison group and the **relative effect** of the intervention (and its 95% CI); **CI:** confidence interval; **MD:** mean difference; **RR:** risk ratio; **PIEB**: programmed intermittent epidural bolus; **CEI**: continuous epidural infusion; **PCEA:** patient-controlled epidural analgesia. | | | | | | |
| **GRADE Working Group grades of evidence** **High certainty:** we are very confident that the true effect lies close to that of the estimate of the effect. **Moderate certainty:** we are moderately confident in the effect estimate: the true effect is likely to be close to the estimate of the effect, but there is a possibility that it is substantially different. **Low certainty:** our confidence in the effect estimate is limited: the true effect may be substantially different from the estimate of the effect. **Very low certainty:** we have very little confidence in the effect estimate: the true effect is likely to be substantially different from the estimate of effect. | | | | | | |

#### Explanations

a. High heterogeneity

b. The included studies involved heterogeneous populations and surgical procedures.

c. There was variability in the definition of outcomes.

d. Analysis contains few studies.

e. Possible small-study effect.

**Supplemental Figure S1. Movement-related pain at 36 hours**

**
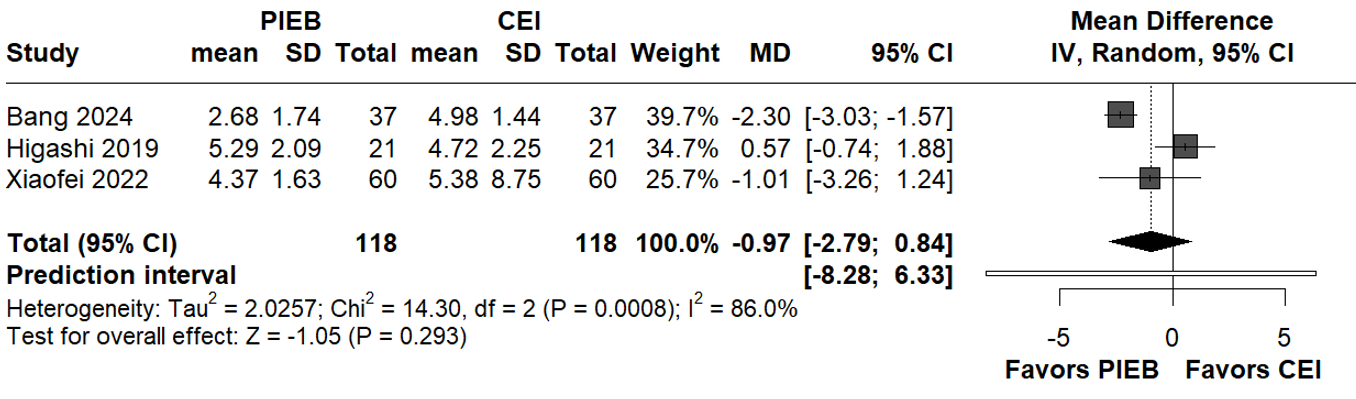
**

*Figure S1. Forest plots comparing PIEB and CEI (both associated with patient-controlled epidural analgesia - PCEA) on postoperative movement-related pain (defined as pain elicited by movement), on a 0-10 scale, at 36 hours. CEI: continuous epidural infusion; PIEB: programmed intermittent epidural bolus.*

**Supplemental Figure S2. Remaining continuous secondary outcomes**

**
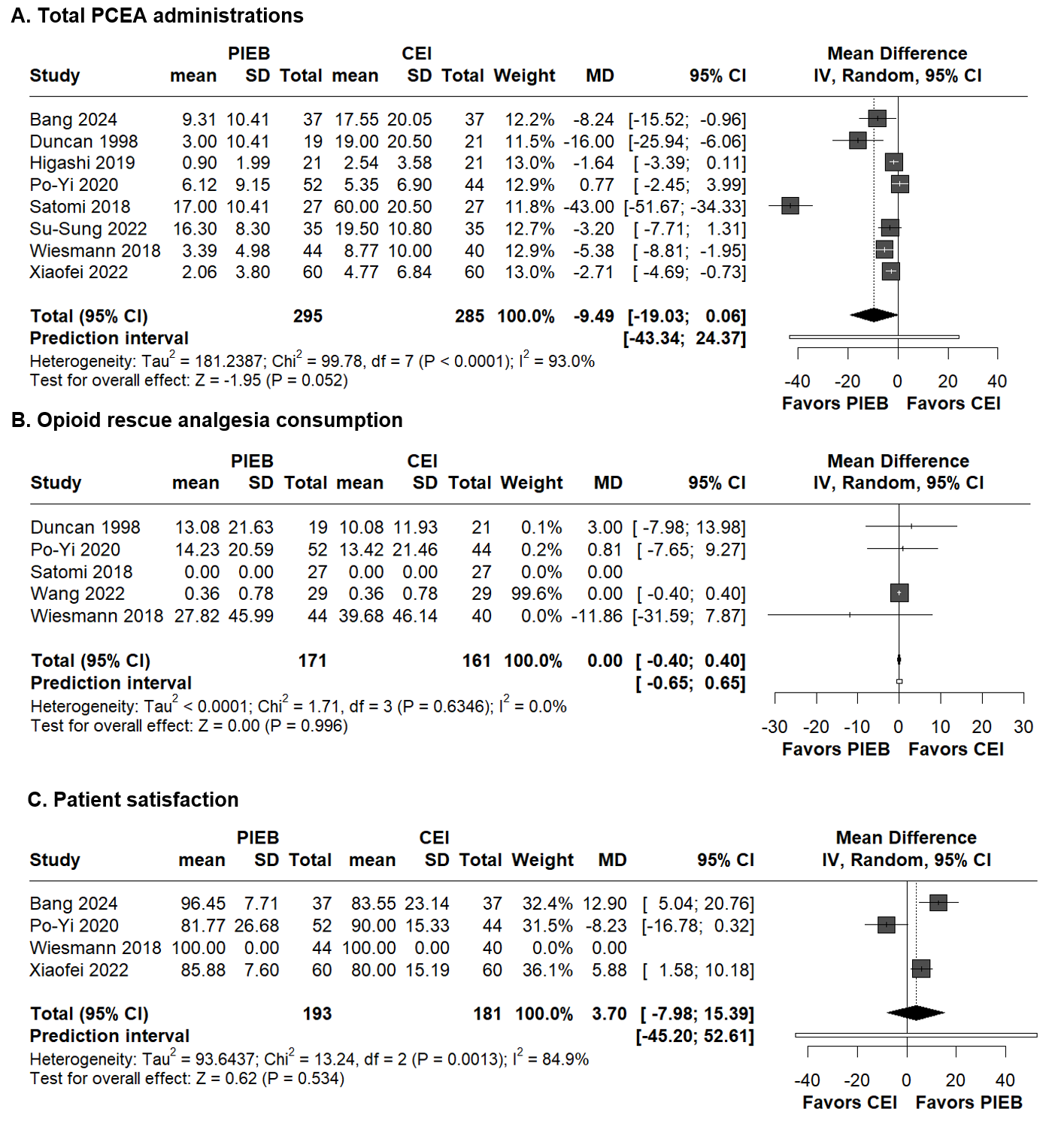
**

*Figure S2. Forest plots comparing PIEB and CEI (both associated with patient-controlled epidural analgesia - PCEA) on: A. Total patient-controlled epidural analgesia (PCEA) administrations delivered; B. Opioid rescue analgesia consumption in oral morphine milligram equivalents; C. Patient satisfaction on a visual analog 0-100 scale. CEI: continuous epidural infusion; PIEB: programmed intermittent epidural bolus.*

**Supplemental Figure S3.1. Remaining binary secondary outcomes**

**
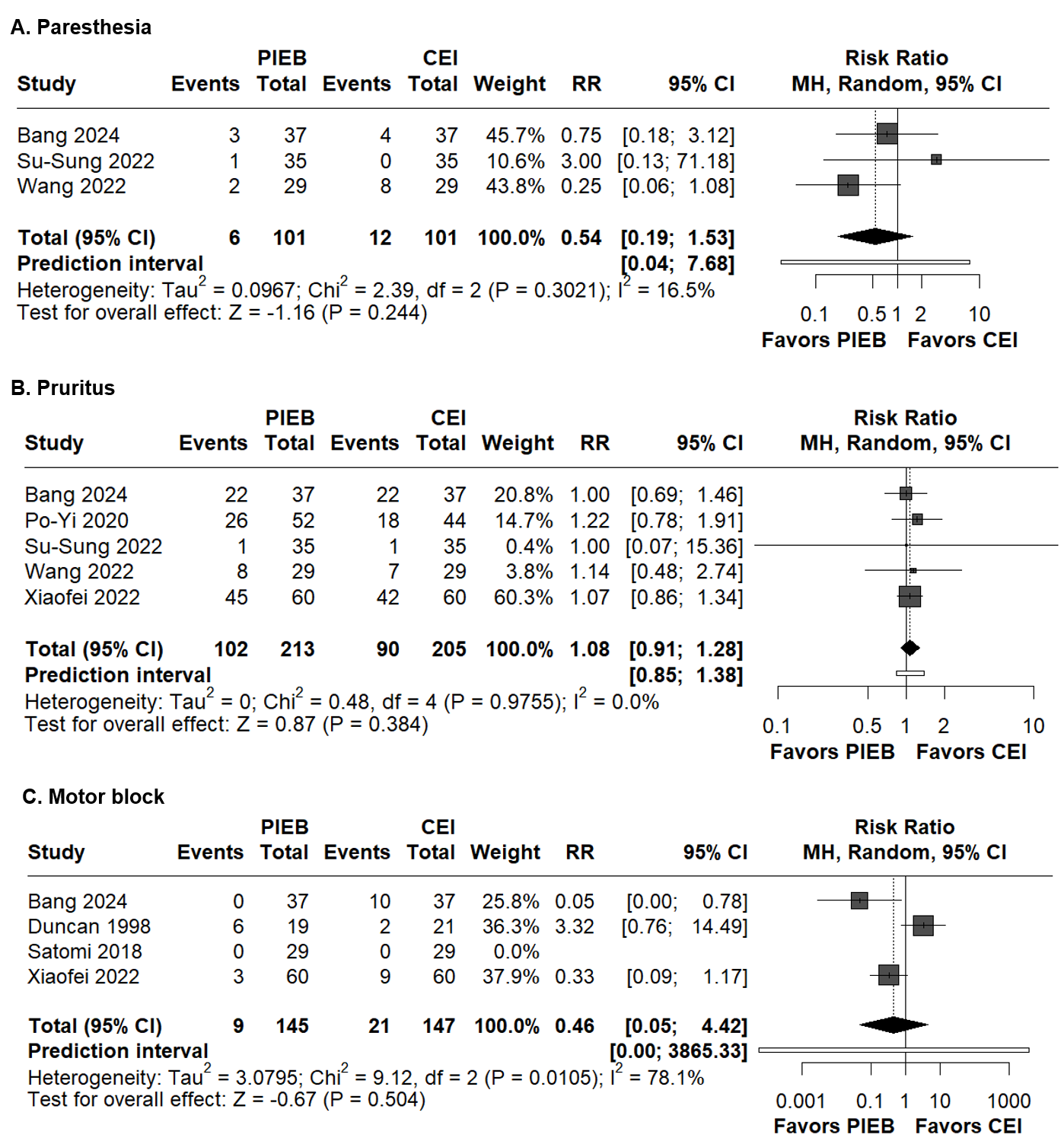
**

*Figure S3.1. Forest plots comparing PIEB and CEI (both associated with patient-controlled epidural analgesia - PCEA) on: A. Patients that reported paresthesia; B. Patients that presented complaints of pruritus; C. Patients presenting motor block at 24 hours follow-up, defined as Bromage scale higher than 0. CEI: continuous epidural infusion; PIEB: programmed intermittent epidural bolus.*

**Supplemental Figure S3.2. Remaining binary secondary outcomes**

**
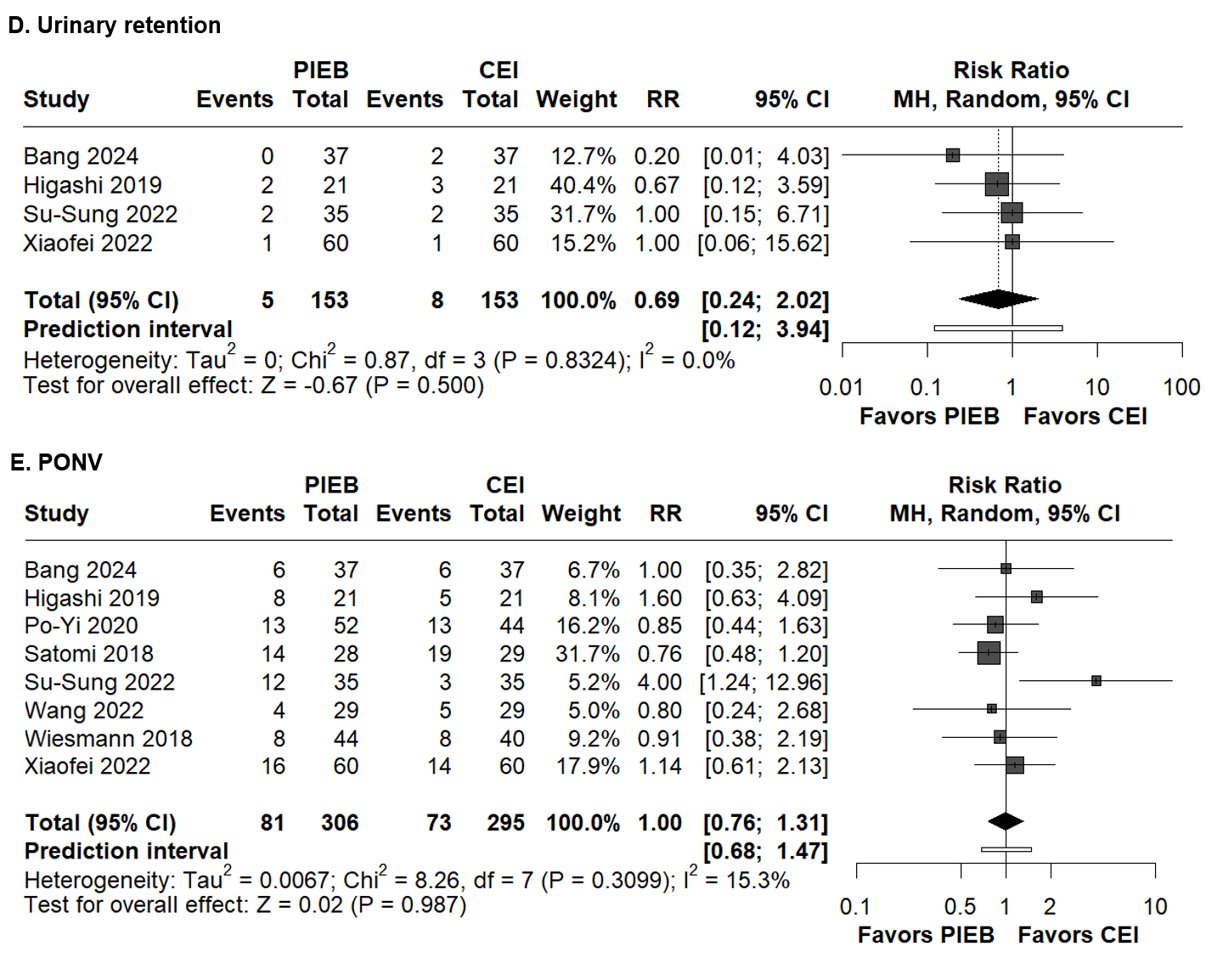
**

*Figure S3.2. Forest plots comparing PIEB and CEI (both associated with patient-controlled epidural analgesia - PCEA) on: D. Patients reporting episodes of urinary retention; E. Patients that presented postoperative nausea and/or vomiting (PONV). CEI: continuous epidural infusion; PIEB: programmed intermittent epidural bolus.*

**Supplemental Figure S4.1. Subgroup analyses: obstetric or non-obstetric setting**

**
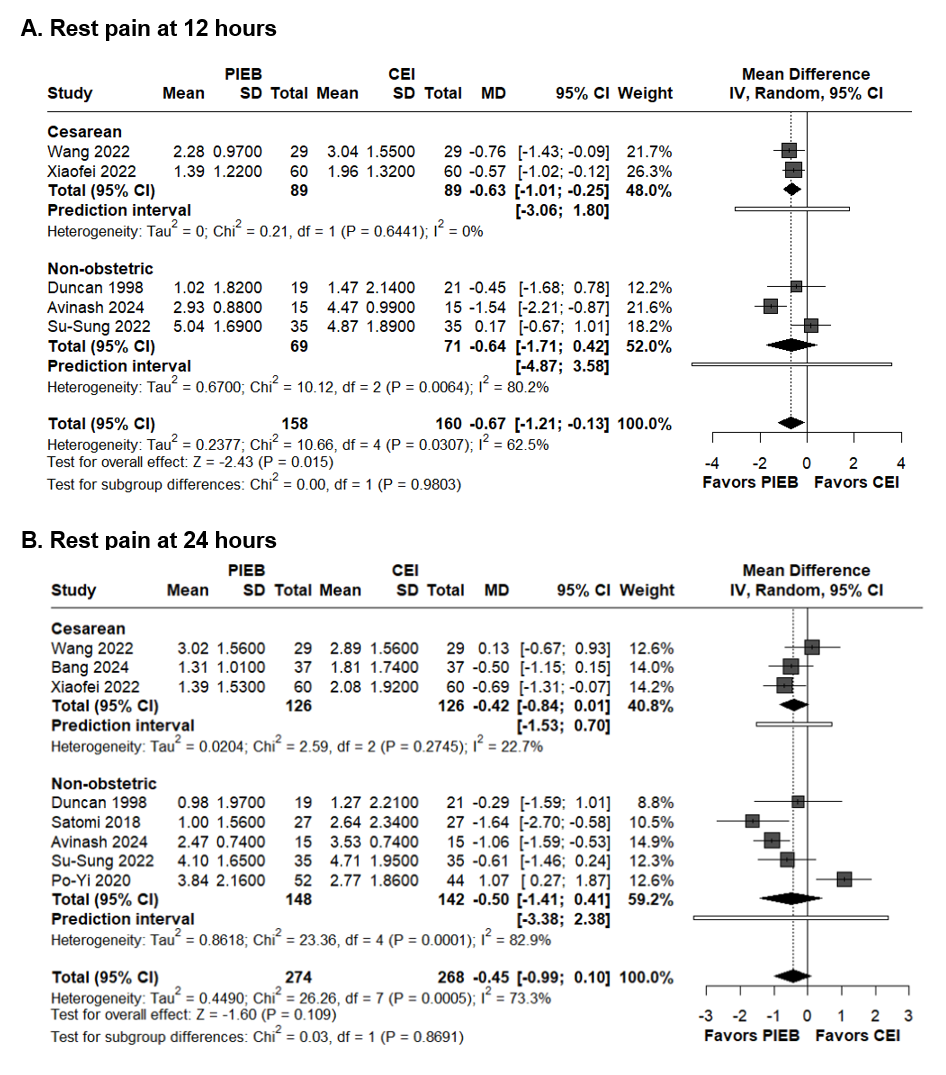
**

*Figure S4.1. Forest plots comparing PIEB and CEI (both associated with patient-controlled epidural analgesia - PCEA) on pain at rest, on a 0-10 scale, at 12 (A) and 24 hours (B). Data is stratified according to whether patients underwent cesarean delivery or not. CEI: continuous epidural infusion; PIEB: programmed intermittent epidural bolus.*

**Supplemental Figure S4.2. Subgroup analyses: obstetric or non-obstetric setting**

**
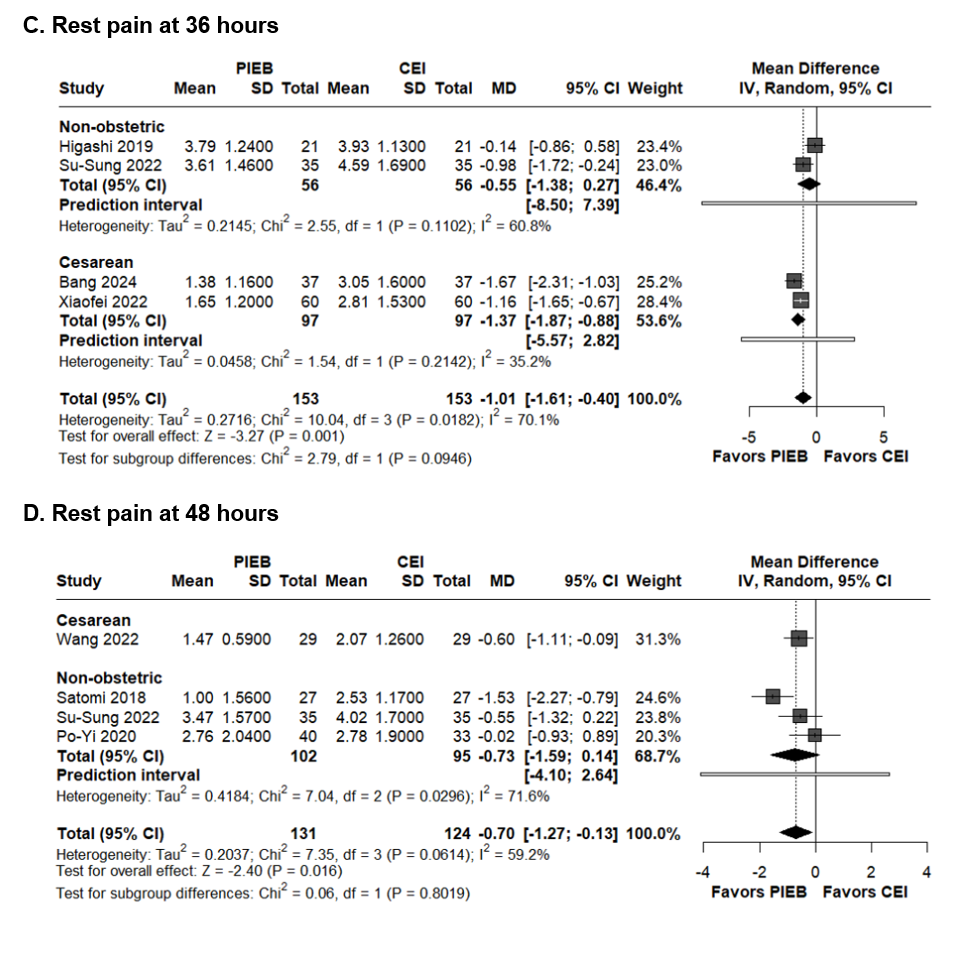
**

*Figure S4.2. Forest plots comparing PIEB and CEI (both associated with patient-controlled epidural analgesia - PCEA) on pain at rest, on a 0-10 scale, at 36 (A) and 48 hours (B). Data is stratified according to whether patients underwent cesarean delivery or not. CEI: continuous epidural infusion; PIEB: programmed intermittent epidural bolus.*

**Supplemental Figure S4.3. Subgroup analyses: obstetric or non-obstetric setting**

**
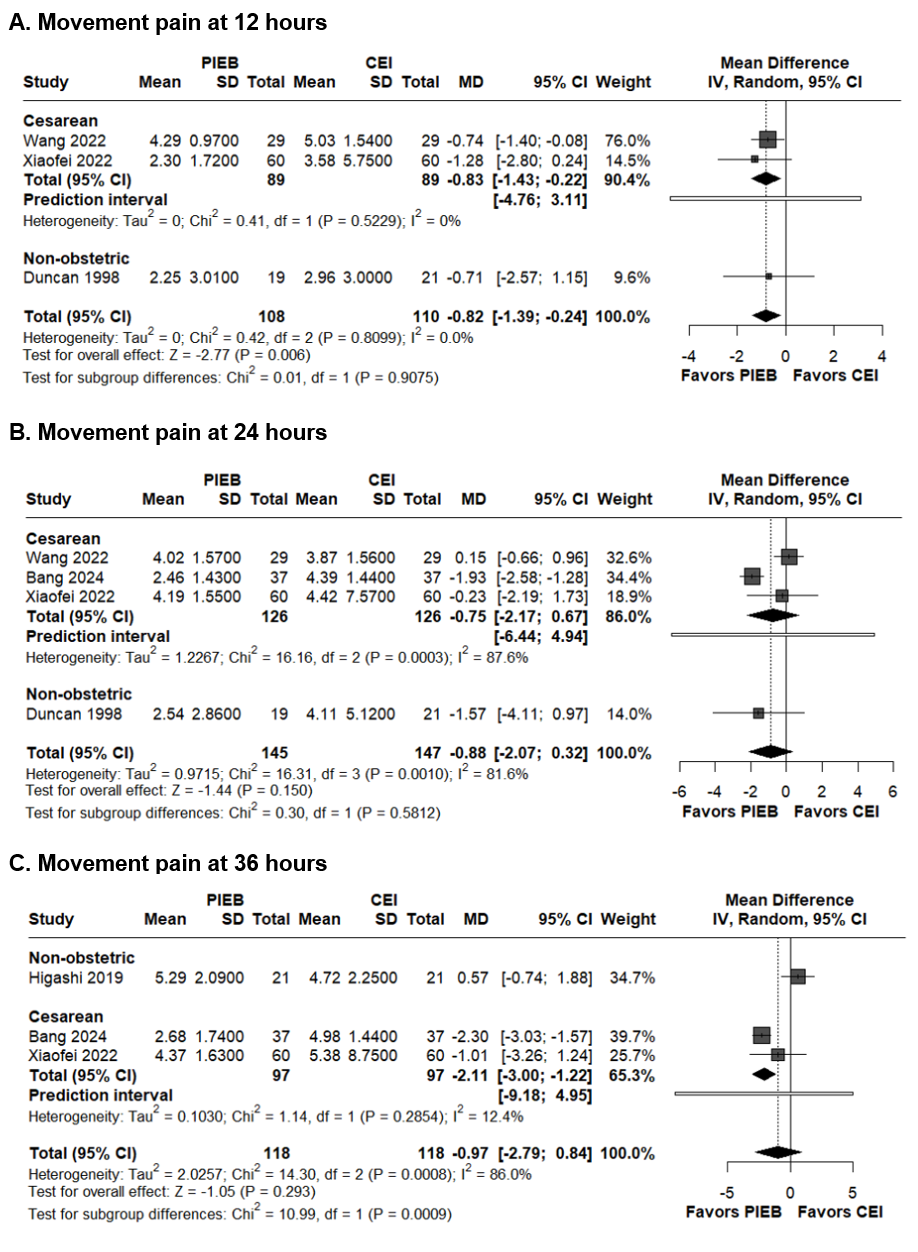
**

*Figure S4.3. Forest plots comparing PIEB and CEI (both associated with patient-controlled epidural analgesia - PCEA) on movement-related pain (defined as pain elicited by movement), on a 0-10 scale, at 12 (A), 24 (B) and 36 hours (C). Data is stratified according to whether patients underwent cesarean delivery or not. CEI: continuous epidural infusion; PIEB: programmed intermittent epidural bolus.*

**Supplemental Figure S4.4. Subgroup analyses: obstetric or non-obstetric setting**

**
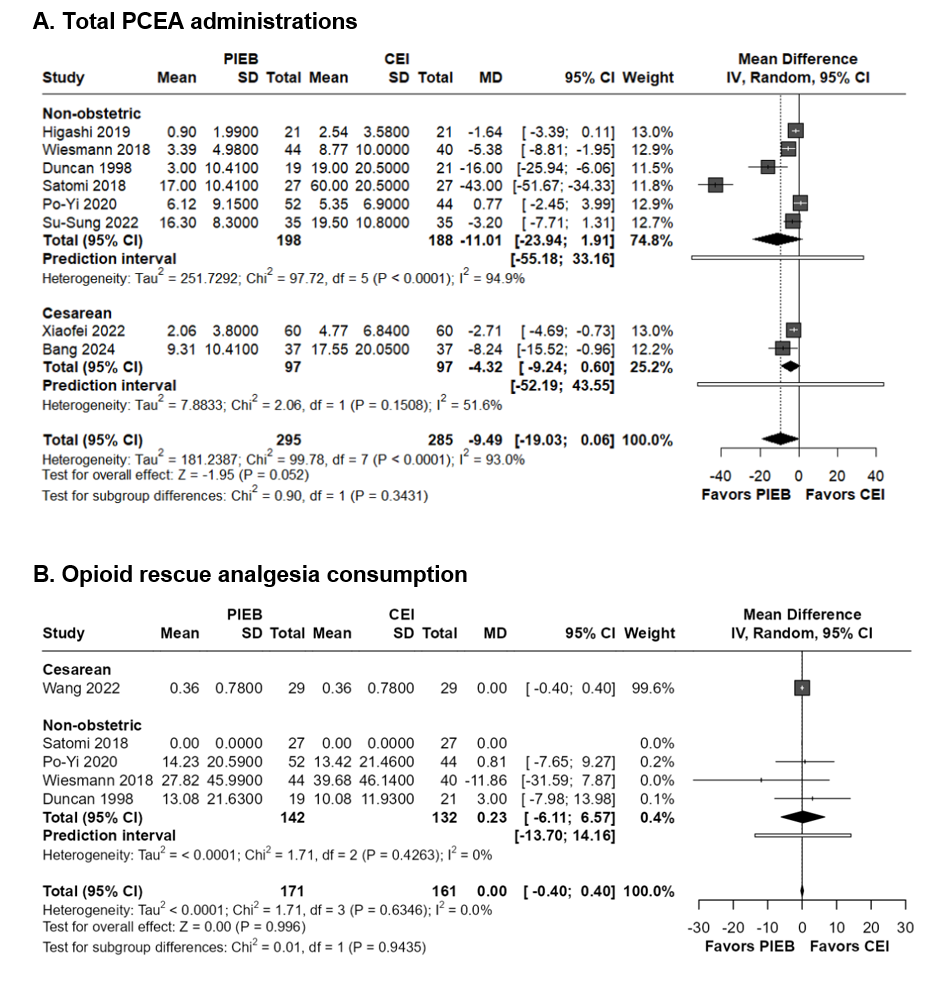
**

*Figure S4.4. Forest plots comparing PIEB and CEI (both associated with patient-controlled epidural analgesia - PCEA) on: A. Total PCEA administrations; B. Opioid rescue analgesia consumption in oral morphine milligram equivalents. Data is stratified according to whether patients underwent cesarean delivery or not. CEI: continuous epidural infusion; PIEB: programmed intermittent epidural bolus.*

**Supplemental Figure S4.5. Subgroup analyses: obstetric or non-obstetric setting**

**
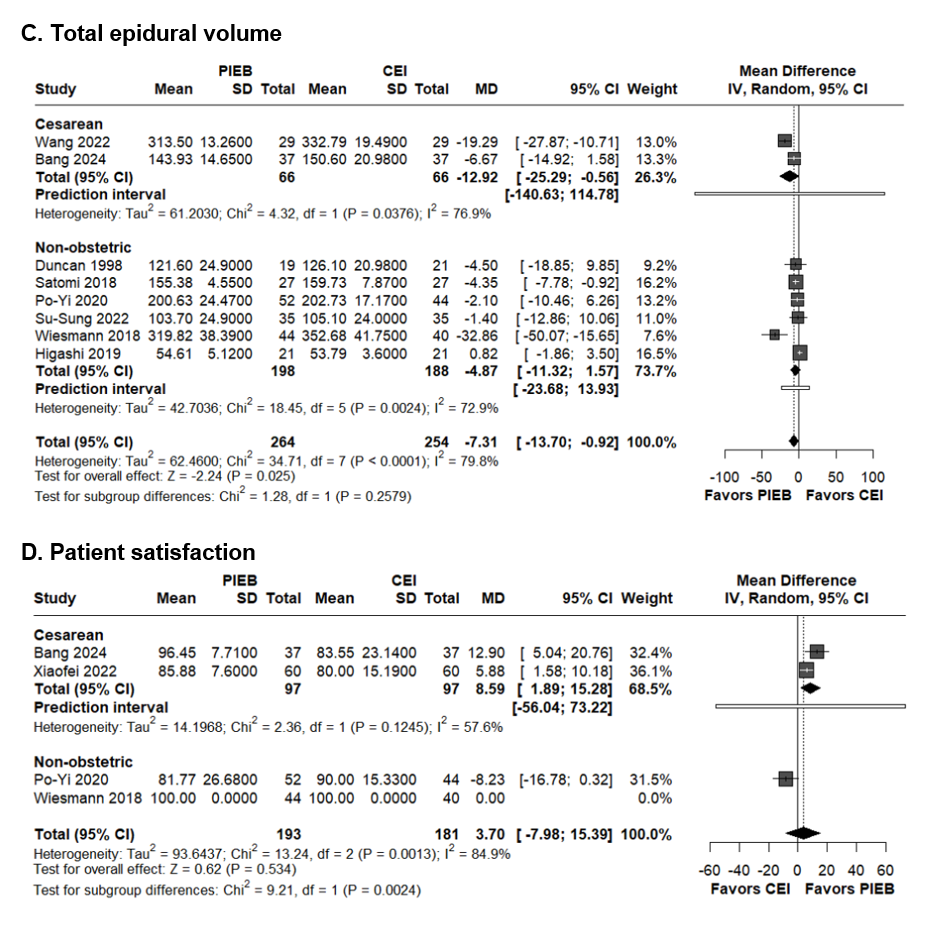
**

*Figure S4.5. Forest plots comparing PIEB and CEI (both associated with patient-controlled epidural analgesia - PCEA) on: C. Total epidural volume administered in mL; D. Patient satisfaction on a visual analog 0-100 scale. Data is stratified according to whether patients underwent cesarean delivery or not. CEI: continuous epidural infusion; PIEB: programmed intermittent epidural bolus.*

**Supplemental Figure S4.6. Subgroup analyses: obstetric or non-obstetric setting**

**
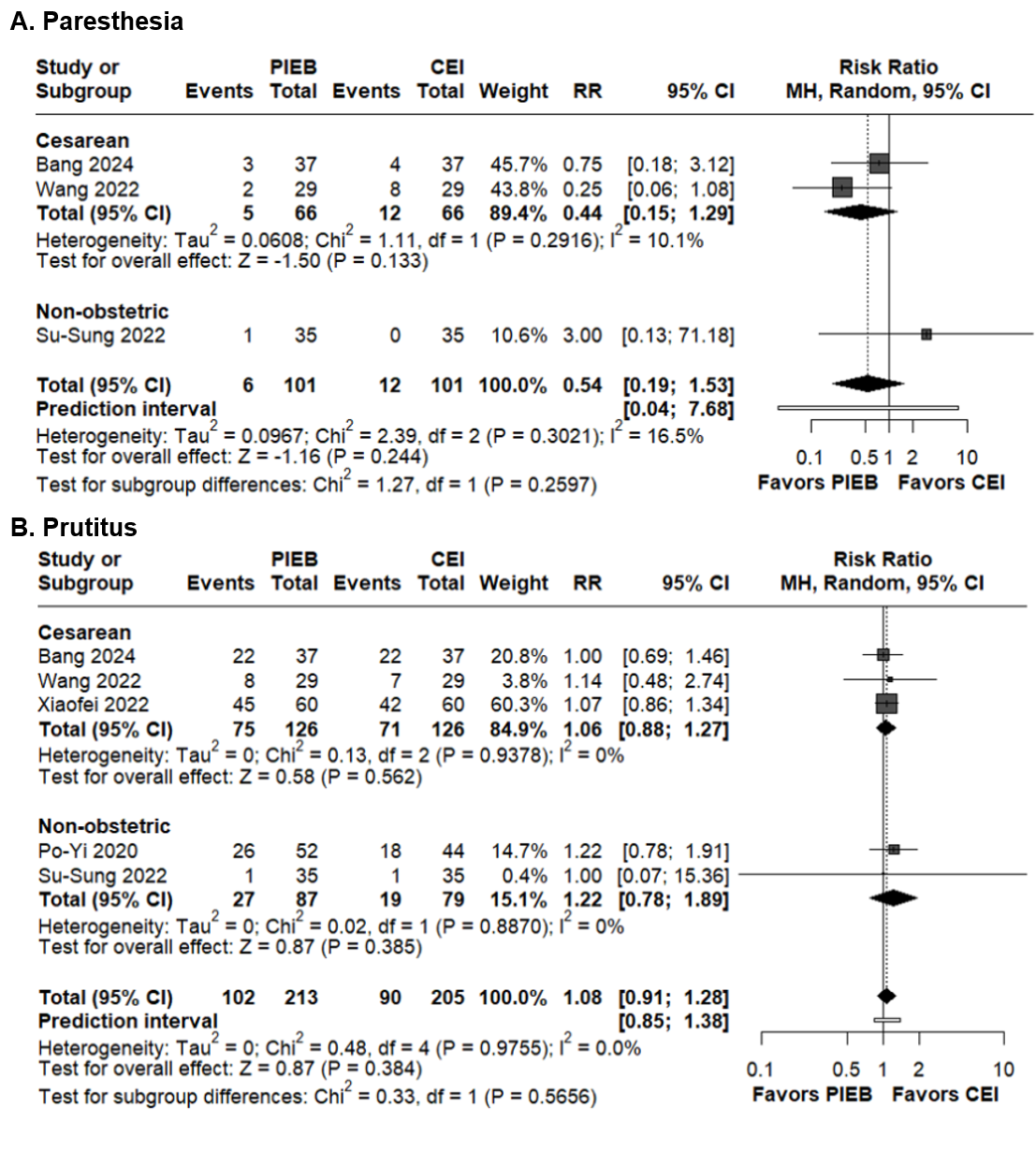
**

*Figure S4.6. Forest plots comparing PIEB and CEI (both associated with patient-controlled epidural analgesia - PCEA) on: A. Patients that reported paresthesia; B. Patients that presented complaints of pruritus. Data is stratified according to whether patients underwent cesarean delivery or not. CEI: continuous epidural infusion; PIEB: programmed intermittent epidural bolus.*

**Supplemental Figure S4.7. Subgroup analyses: obstetric or non-obstetric setting**

**
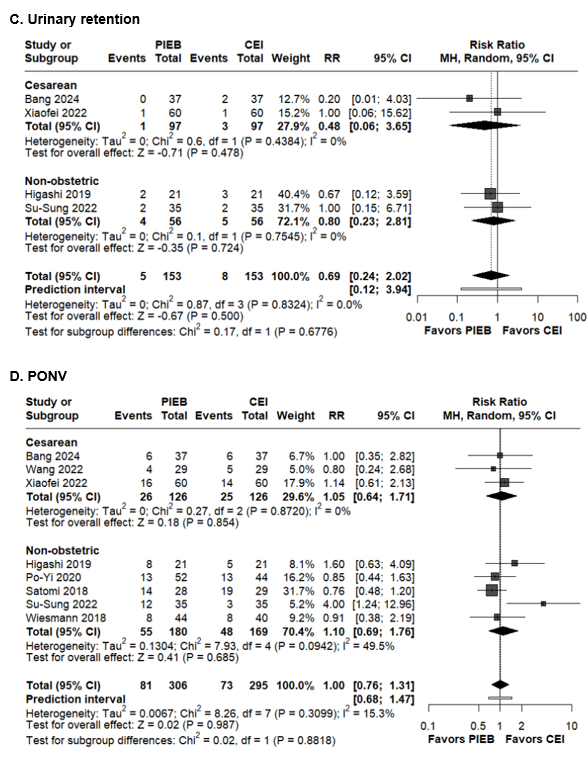
**

*Figure S4.7. Forest plots comparing PIEB and CEI (both associated with patient-controlled epidural analgesia - PCEA) on: C. Patients reporting episodes of urinary retention; D. Patients that presented with postoperative nausea and/or vomiting (PONV). Data is stratified according to whether patients underwent cesarean delivery or not. CEI: continuous epidural infusion; PIEB: programmed intermittent epidural bolus.*

**Supplemental Figure S4.8. Subgroup analyses: obstetric or non-obstetric setting**

**
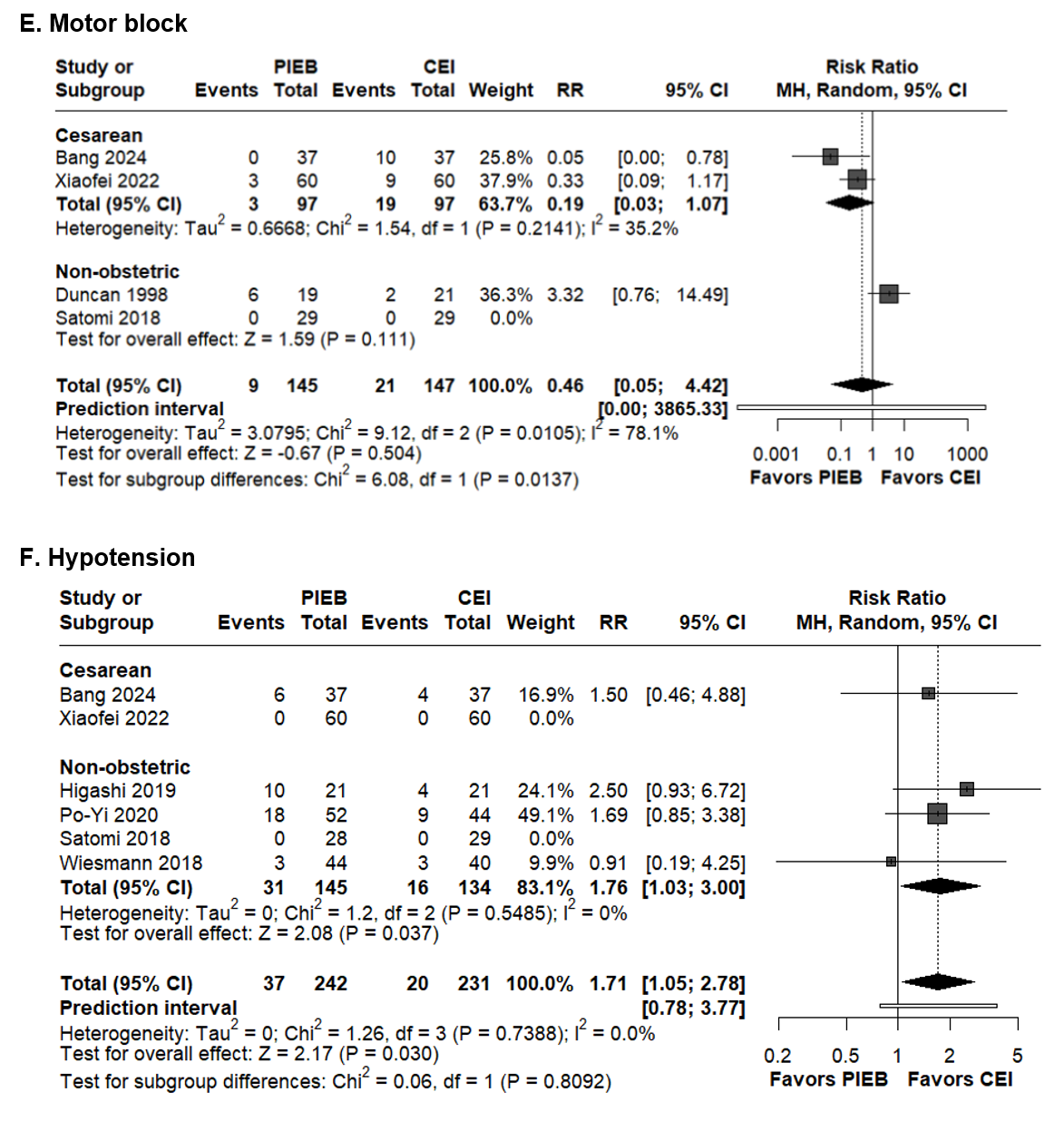
**

*Figure S4.8. Forest plots comparing PIEB and CEI (both associated with patient-controlled epidural analgesia - PCEA) on: E. Patients presenting motor block at 24 hours follow-up; F. the occurrence of postoperative hypotension. Data is stratified according to whether patients underwent cesarean delivery or not. CEI: continuous epidural infusion; PIEB: programmed intermittent epidural bolus.*

**Supplemental Figure S5.1. Subgroup analyses: epidural infusion flow rate**

**
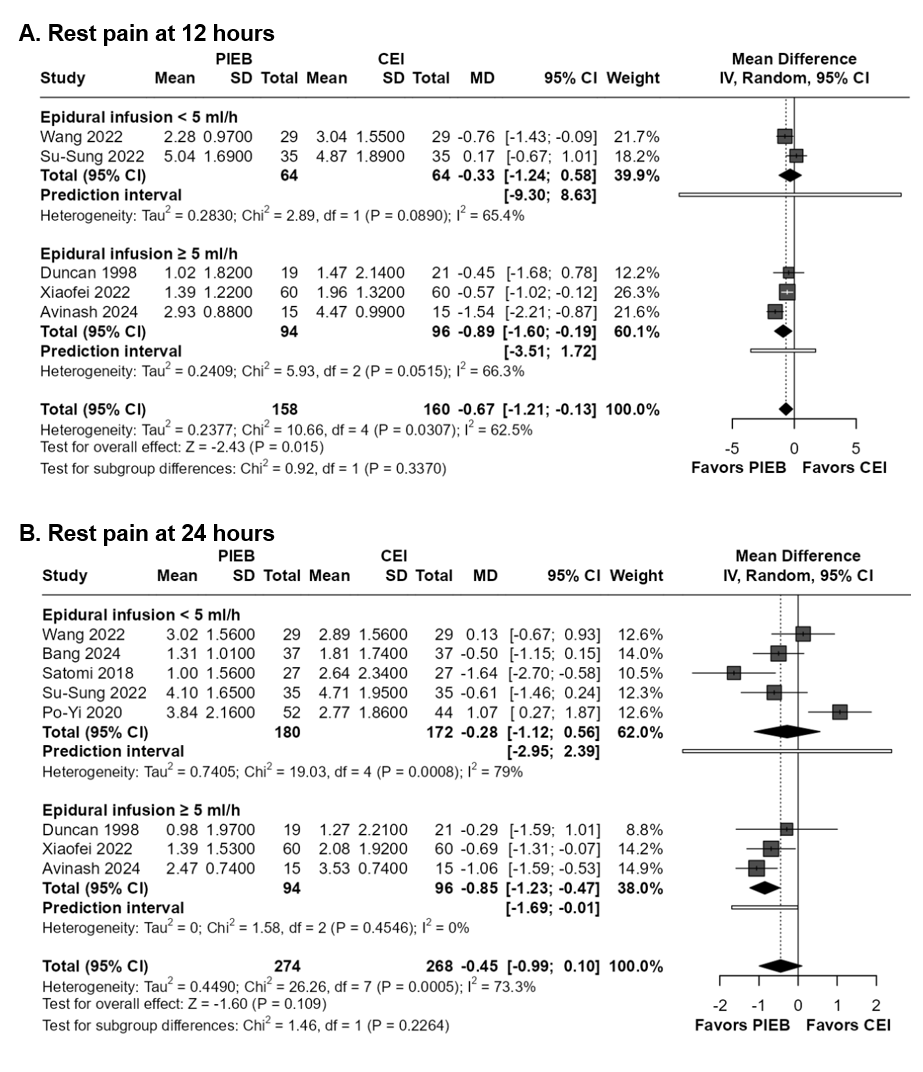
**

*Figure S5.1. Forest plots comparing PIEB and CEI (both associated with patient-controlled epidural analgesia - PCEA) on pain at rest, on a 0-10 scale, at 12 (A) and 24 hours (B). Data is stratified according to whether epidural infusion rate was lower than 5 mL/h or not. CEI: continuous epidural infusion; PIEB: programmed intermittent epidural bolus.*

**Supplemental Figure S5.2. Subgroup analyses: epidural infusion flow rate**

**
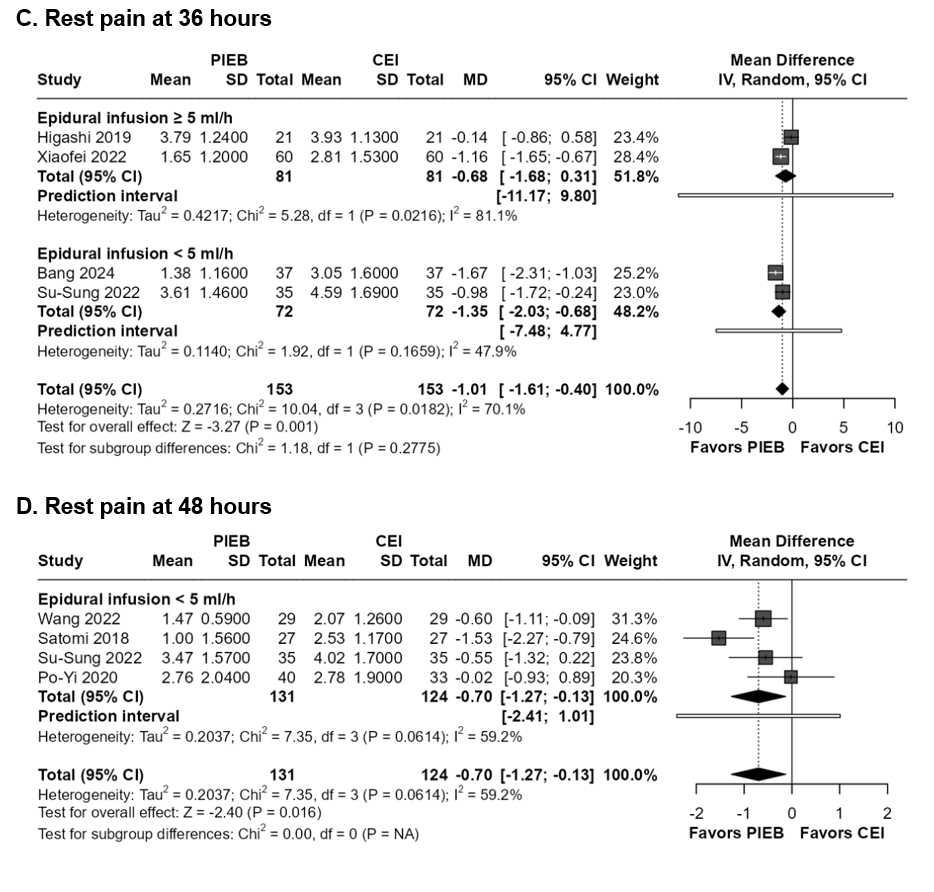
**

*Figure S5.2. Forest plots comparing PIEB and CEI (both associated with patient-controlled epidural analgesia - PCEA) on pain at rest, on a 0-10 scale, at 36 (A) and 48 hours (B). Data is stratified according to whether epidural infusion rate was lower than 5 mL/h or not. CEI: continuous epidural infusion; PIEB: programmed intermittent epidural bolus.*

**Supplemental Figure S5.3. Subgroup analyses: epidural infusion flow rate**

**
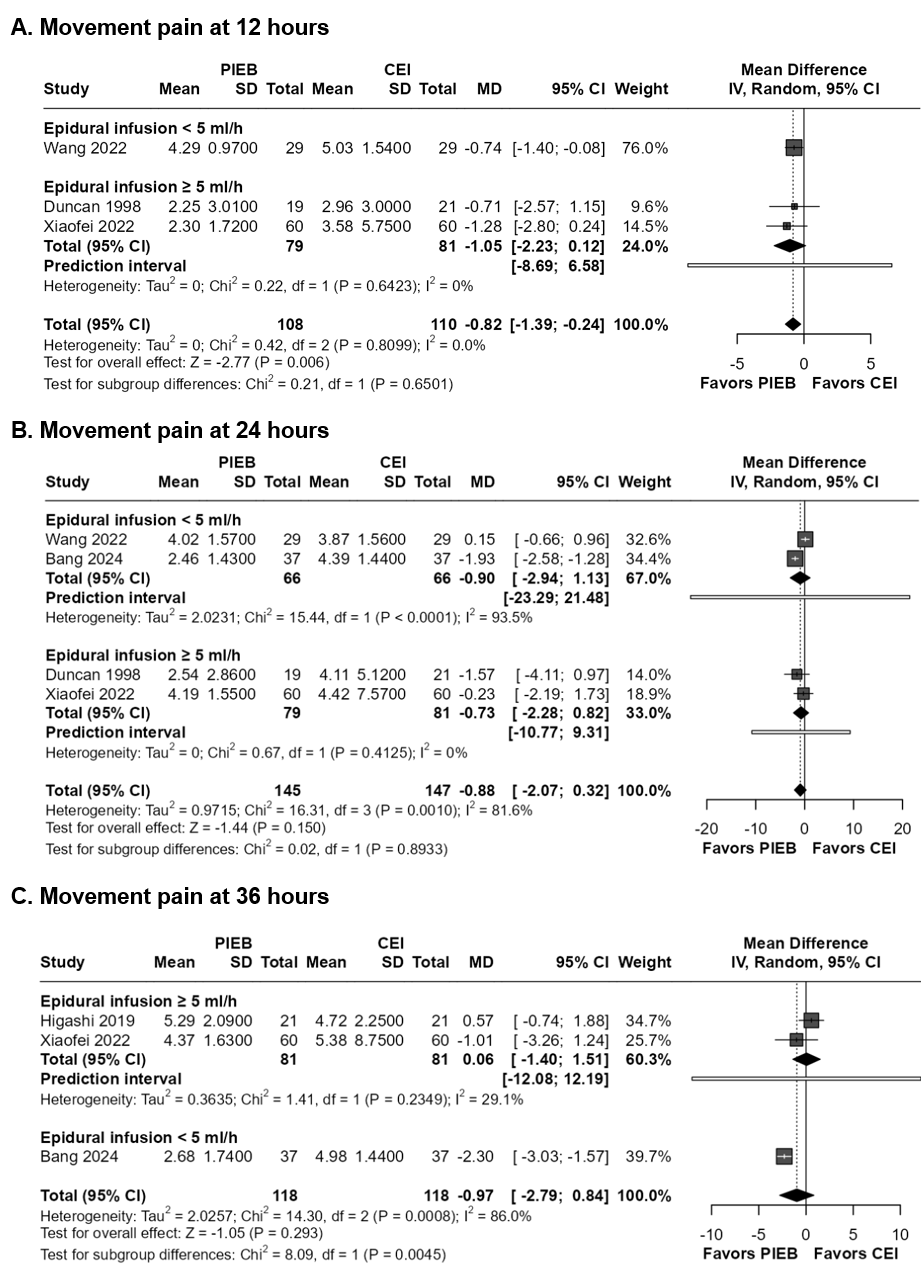
**

*Figure S5.3. Forest plots comparing PIEB and CEI (both associated with patient-controlled epidural analgesia - PCEA) on movement-related pain (defined as pain elicited by movement), on a 0-10 scale, at 12 (A), 24 (B) and 36 hours (C). Data is stratified according to whether epidural infusion rate was lower than 5 mL/h or not. CEI: continuous epidural infusion; PIEB: programmed intermittent epidural bolus.*

**Supplemental Figure S5.4. Subgroup analyses: epidural infusion flow rate**

**
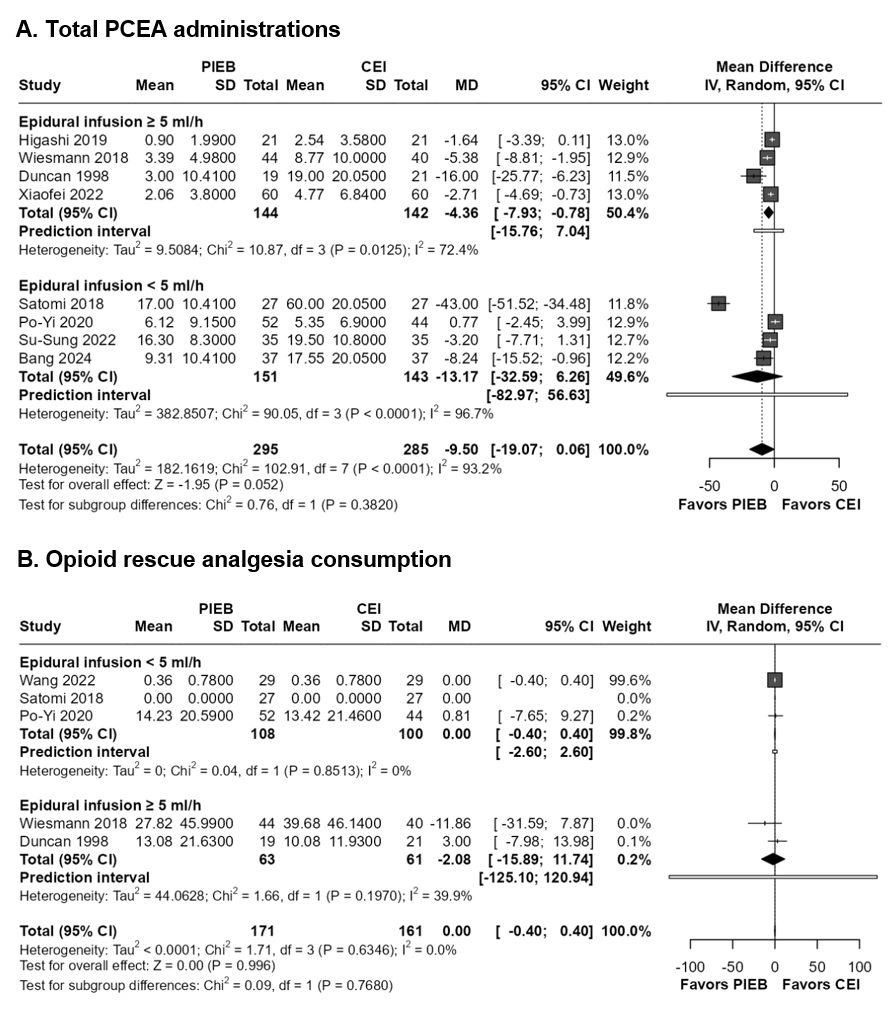
**

*Figure S5.4. Forest plots comparing PIEB and CEI (both associated with patient-controlled epidural analgesia - PCEA) on: A. Total PCEA administrations; B. Opioid rescue analgesia consumption in oral morphine milligram equivalents. Data is stratified according to whether epidural infusion rate was lower than 5 mL/h or not. CEI: continuous epidural infusion; PIEB: programmed intermittent epidural bolus.*

**Supplemental Figure S5.5. Subgroup analyses: epidural infusion flow rate**

**
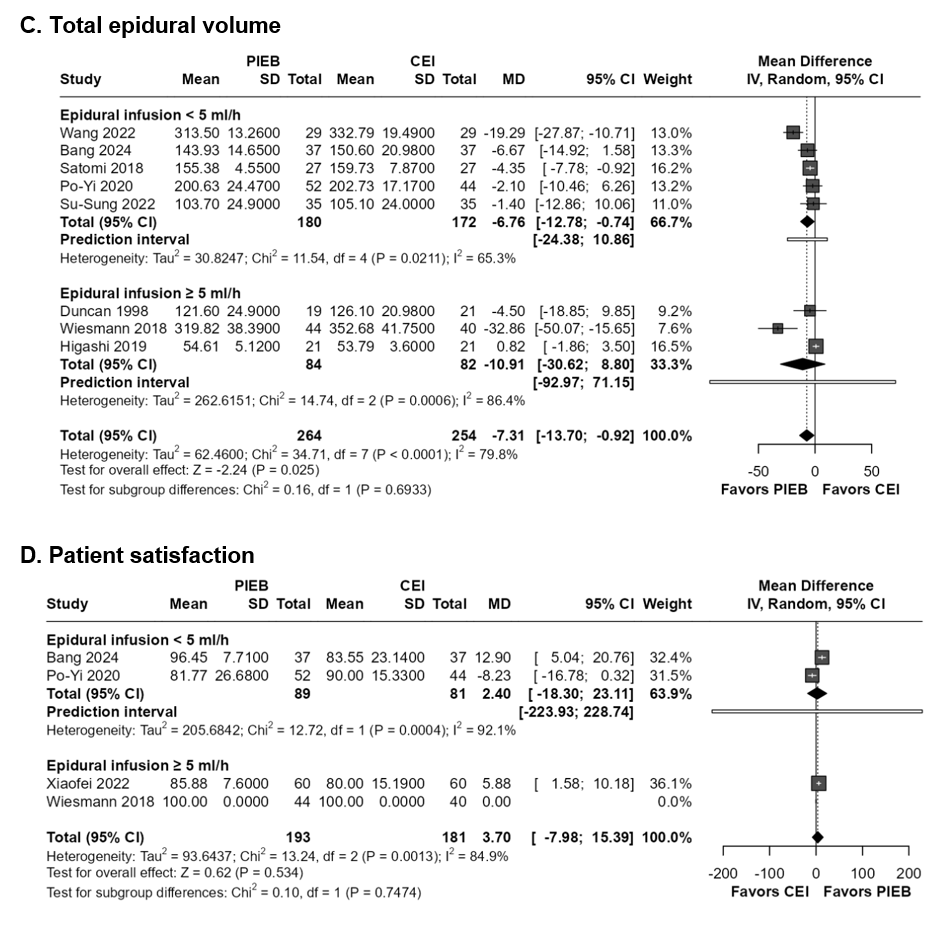
**

*Figure S5.5. Forest plots comparing PIEB and CEI (both associated with patient-controlled epidural analgesia - PCEA) on: C. Total epidural volume administered in mL; D. Patient satisfaction on a visual analog 0-100 scale. Data is stratified according to whether epidural infusion rate was lower than 5 mL/h or not. CEI: continuous epidural infusion; PIEB: programmed intermittent epidural bolus.*

**Supplemental Figure S5.6. Subgroup analyses: epidural infusion flow rate**

**
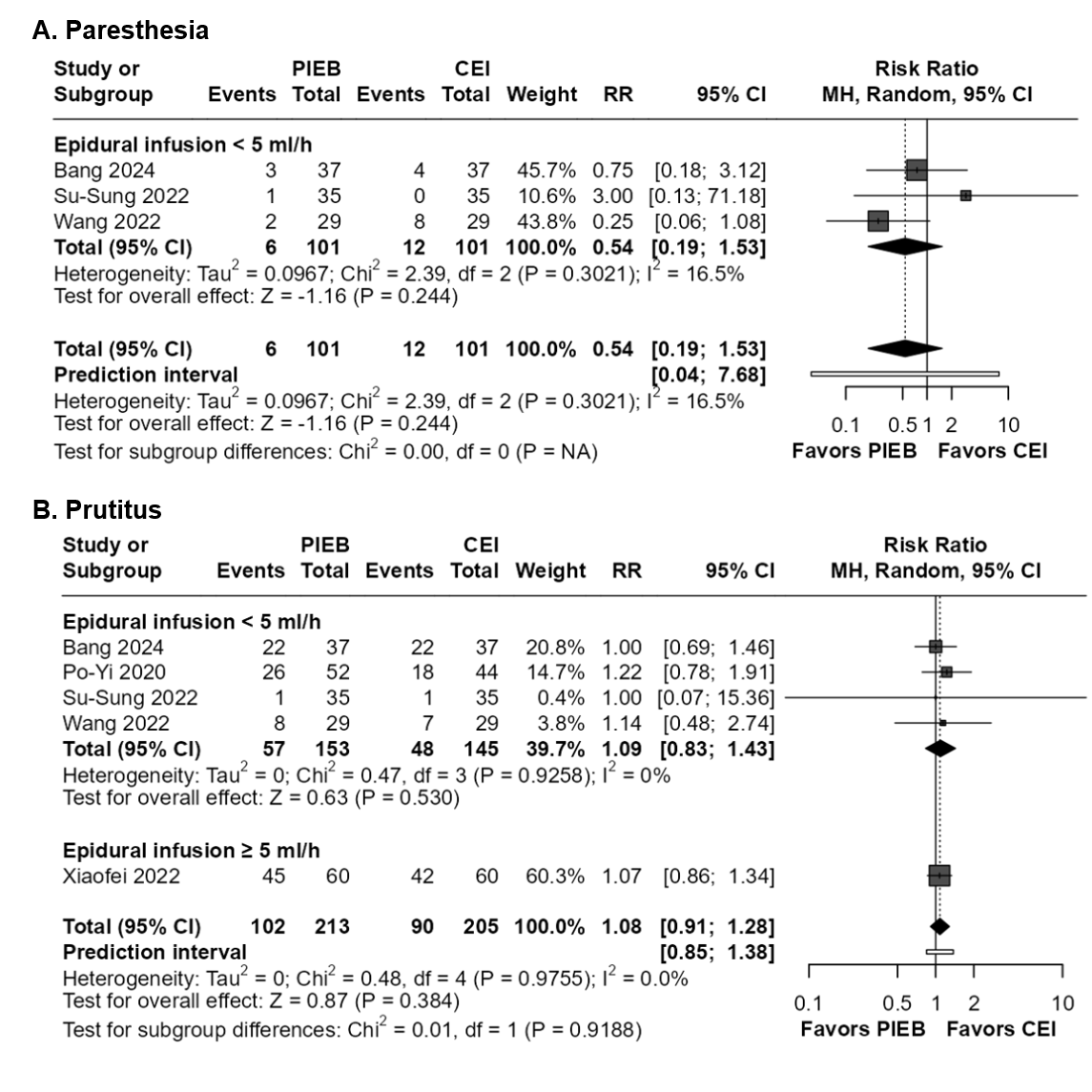
**

*Figure S5.6. Forest plots comparing PIEB and CEI (both associated with patient-controlled epidural analgesia - PCEA) on: A. Patients that reported paresthesia; B. Patients that presented complaints of pruritus. Data is stratified according to whether epidural infusion rate was lower than 5 mL/h or not. CEI: continuous epidural infusion; PIEB: programmed intermittent epidural bolus.*

**Supplemental Figure S5.7. Subgroup analyses: epidural infusion flow rate**

**
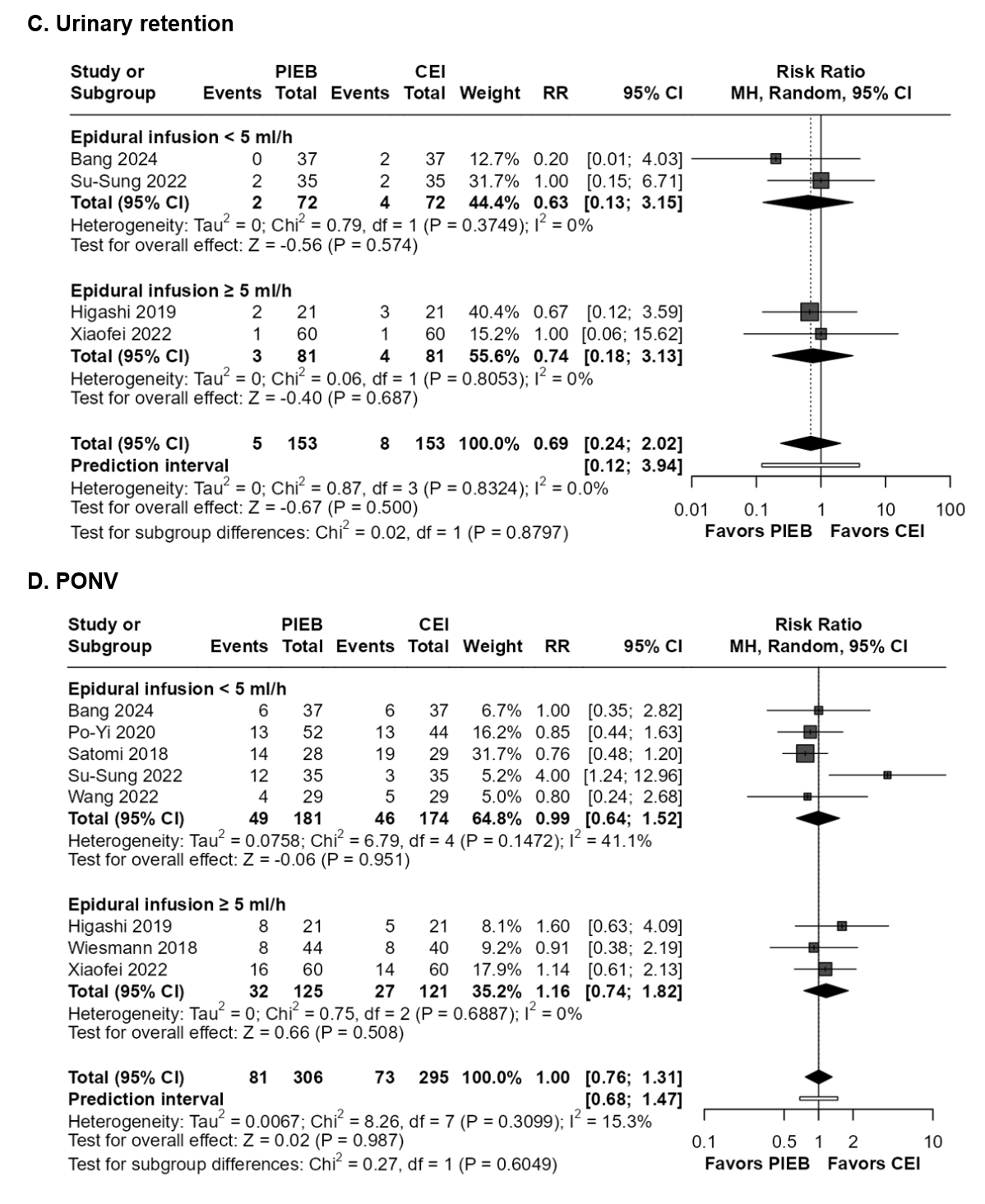
**

*Figure S5.7. Forest plots comparing PIEB and CEI (both associated with patient-controlled epidural analgesia - PCEA) on: C. Patients reporting episodes of urinary retention; D. Patients that presented with postoperative nausea and/or vomiting (PONV). Data is stratified according to whether epidural infusion rate was lower than 5 mL/h or not. CEI: continuous epidural infusion; PIEB: programmed intermittent epidural bolus.*

**Supplemental Figure S5.8. Subgroup analyses: epidural infusion flow rate**

**
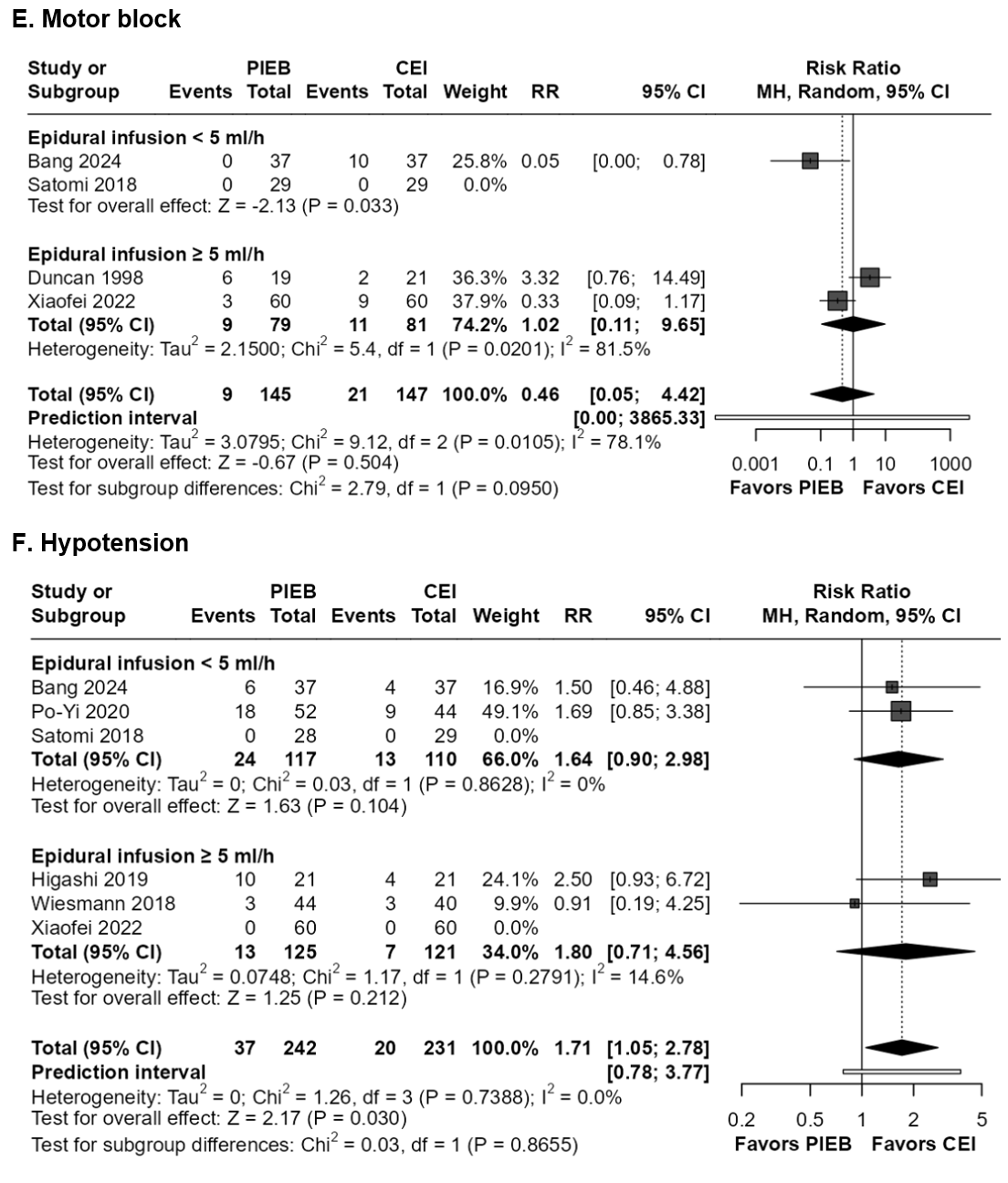
**

*Figure S5.8. Forest plots comparing PIEB and CEI (both associated with patient-controlled epidural analgesia - PCEA) on: E. Patients presenting motor block at 24 hours follow-up; F. the occurrence of postoperative hypotension. Data is stratified according to whether epidural infusion rate was lower than 5 mL/h or not. CEI: continuous epidural infusion; PIEB: programmed intermittent epidural bolus.*

**Supplemental Figure S6.1. Subgroup analyses: risk of bias stratification**

**
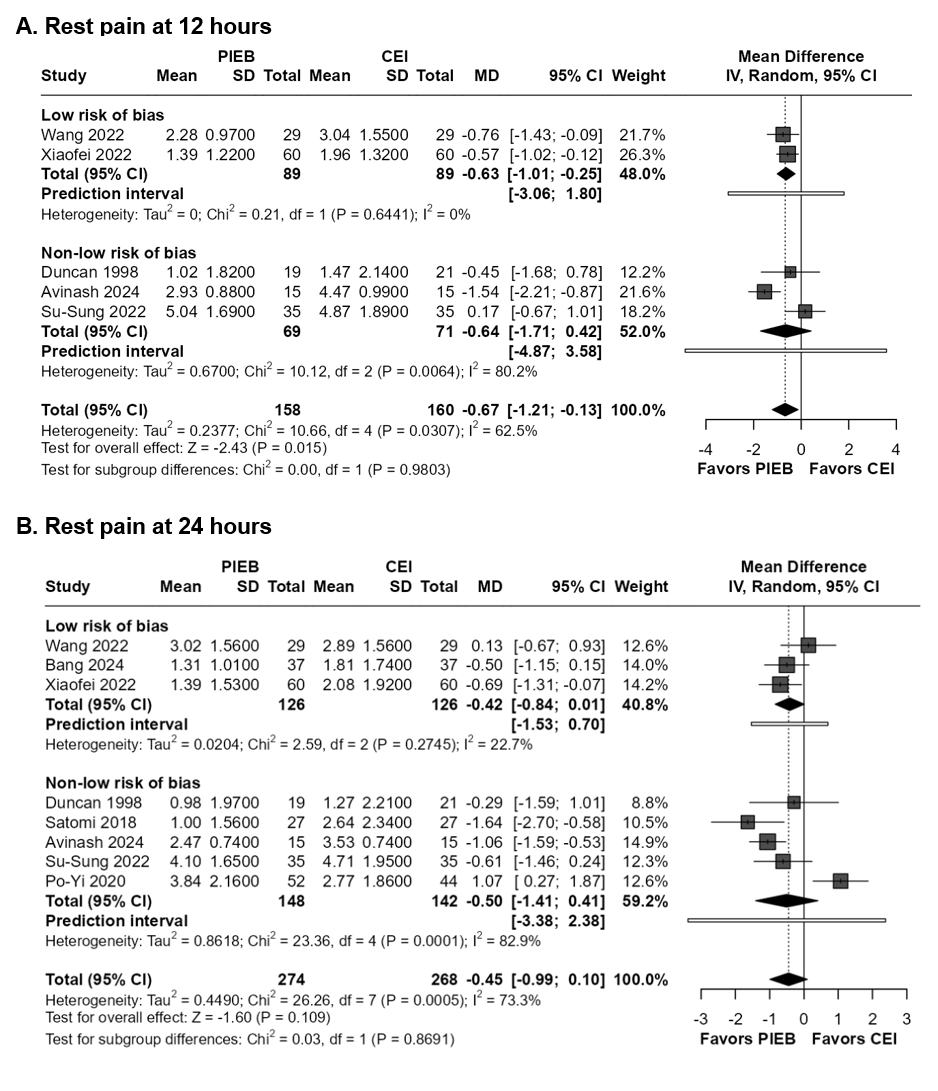
**

*Figure S6.1. Forest plots comparing PIEB and CEI (both associated with patient-controlled epidural analgesia - PCEA) on pain at rest, on a 0-10 scale, at 12 (A) and 24 hours (B). Data is stratified according to whether studies were assessed as presenting a low risk of bias or not. CEI: continuous epidural infusion; PIEB: programmed intermittent epidural bolus.*

**Supplemental Figure S6.2. Subgroup analyses: risk of bias stratification**

**
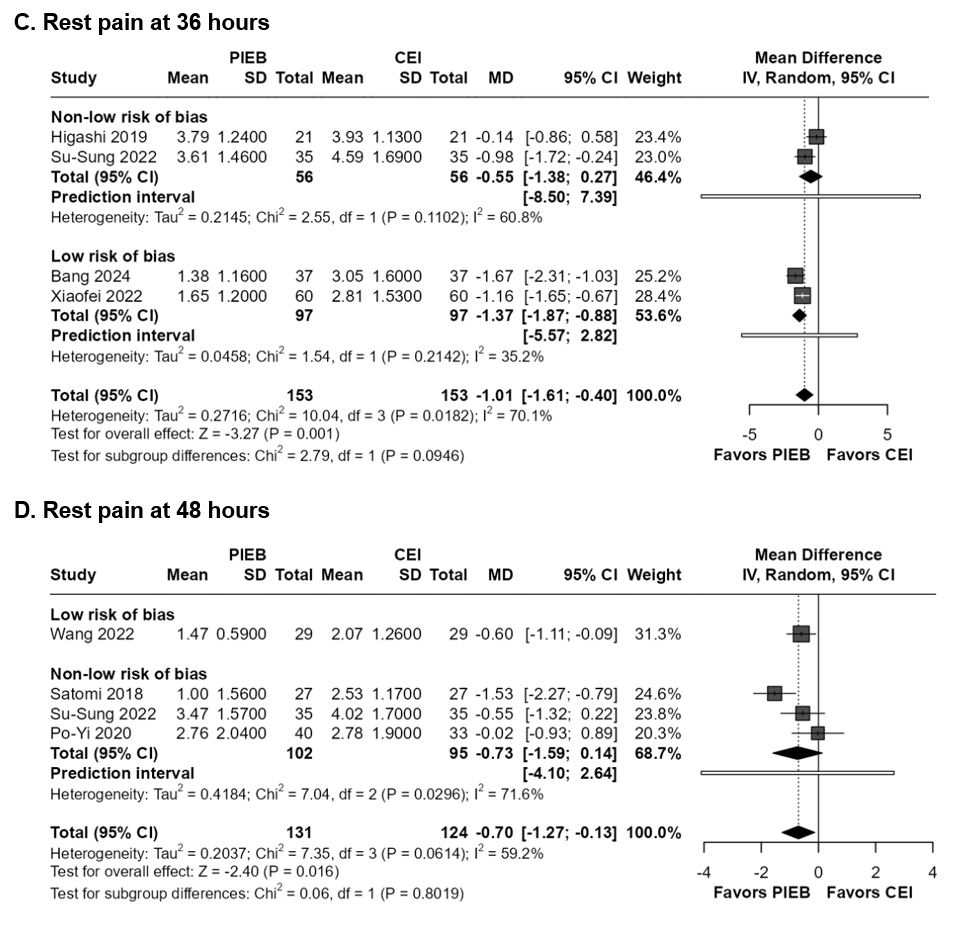
**

*Figure S6.2. Forest plots comparing PIEB and CEI (both associated with patient-controlled epidural analgesia - PCEA) on pain at rest, on a 0-10 scale, at 36 (A) and 48 hours (B). Data is stratified according to whether studies were assessed as presenting a low risk of bias or not. CEI: continuous epidural infusion; PIEB: programmed intermittent epidural bolus.*

**Supplemental Figure S6.3. Subgroup analyses: risk of bias stratification**

**
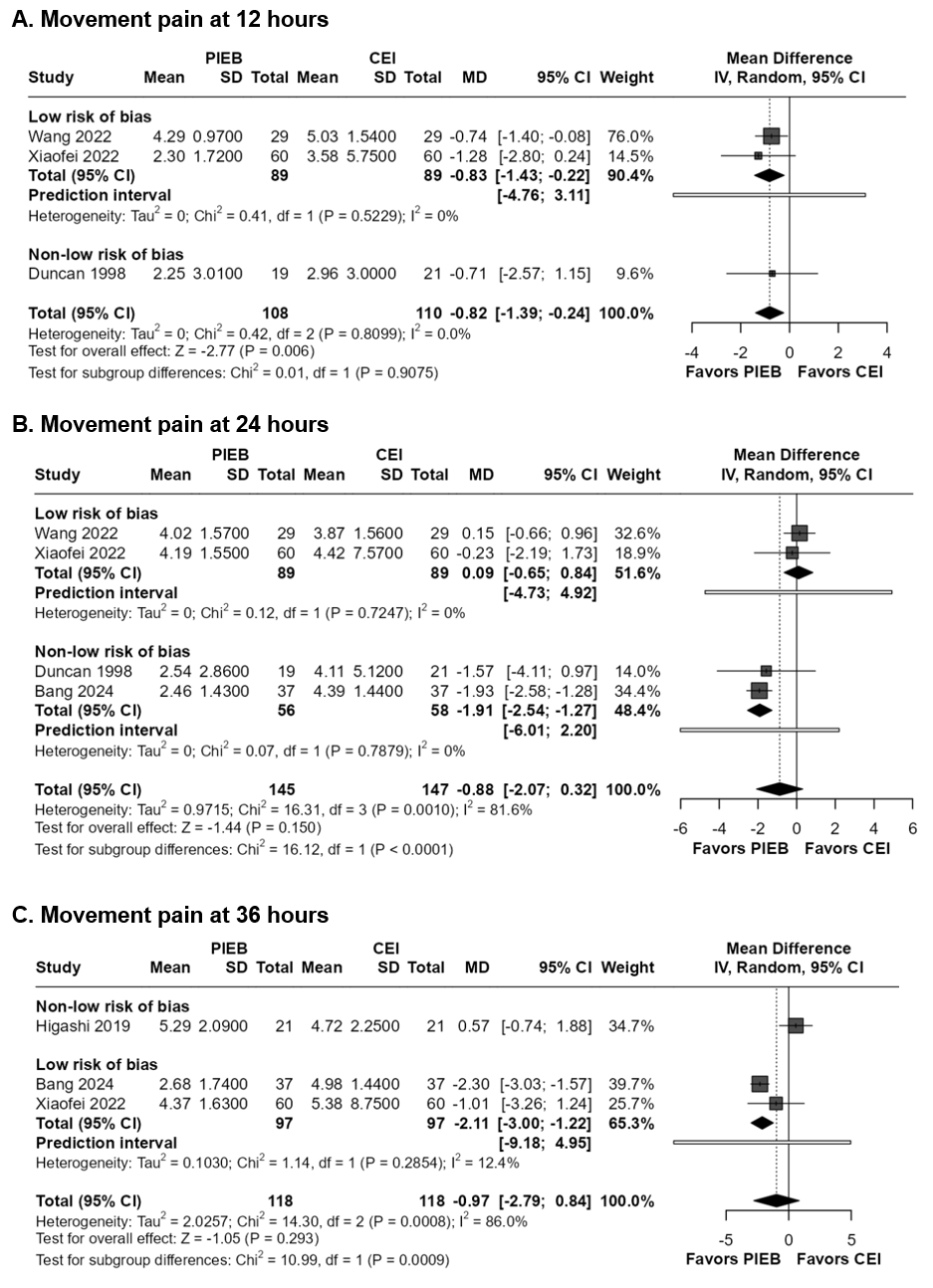
**

*Figure S6.3. Forest plots comparing PIEB and CEI (both associated with patient-controlled epidural analgesia - PCEA) on movement-related pain (defined as pain elicited by movement), on a 0-10 scale, at 12 (A), 24 (B) and 36 hours (C). Data is stratified according to whether studies were assessed as presenting a low risk of bias or not. CEI: continuous epidural infusion; PIEB: programmed intermittent epidural bolus.*

**Supplemental Figure S6.4. Subgroup analyses: risk of bias stratification**

**
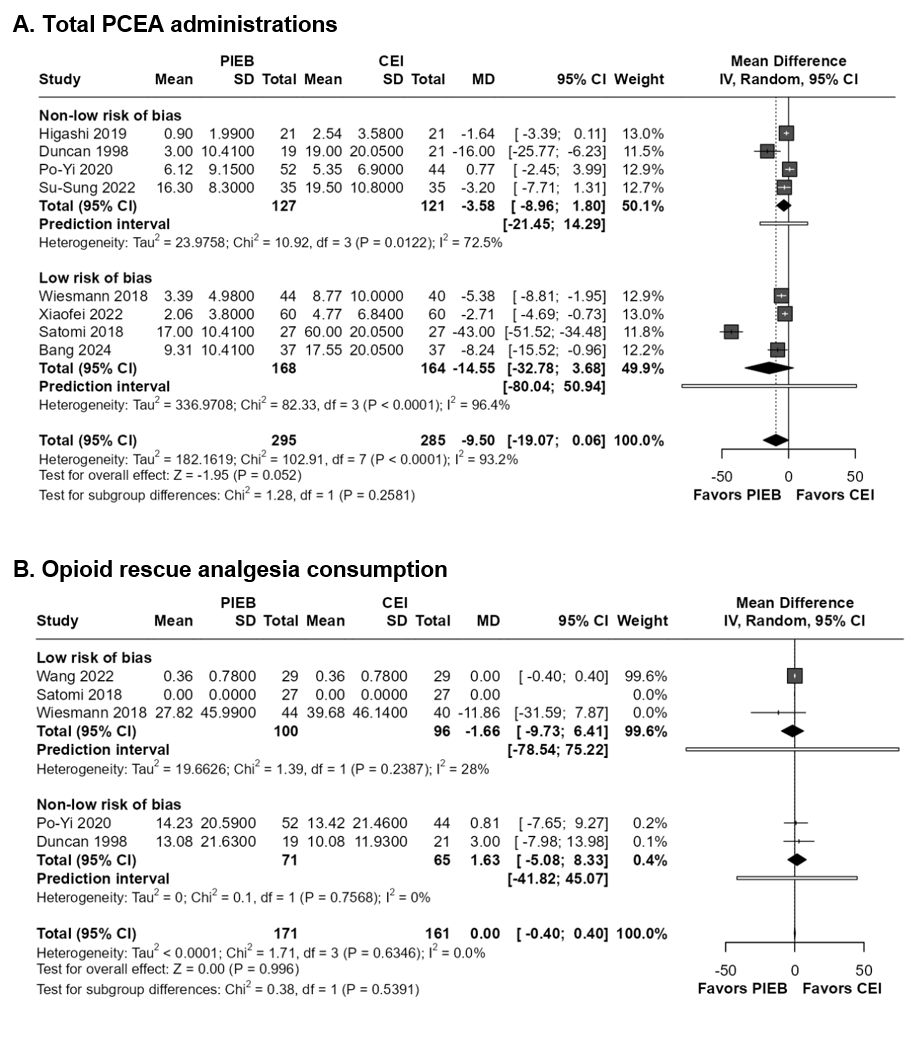
**

*Figure S6.4. Forest plots comparing PIEB and CEI (both associated with patient-controlled epidural analgesia - PCEA) on: A. Total PCEA administrations; B. Opioid rescue analgesia consumption in oral morphine milligram equivalents. Data is stratified according to whether studies were assessed as presenting a low risk of bias or not. CEI: continuous epidural infusion; PIEB: programmed intermittent epidural bolus.*

**Supplemental Figure S6.5. Subgroup analyses: risk of bias stratification**

**
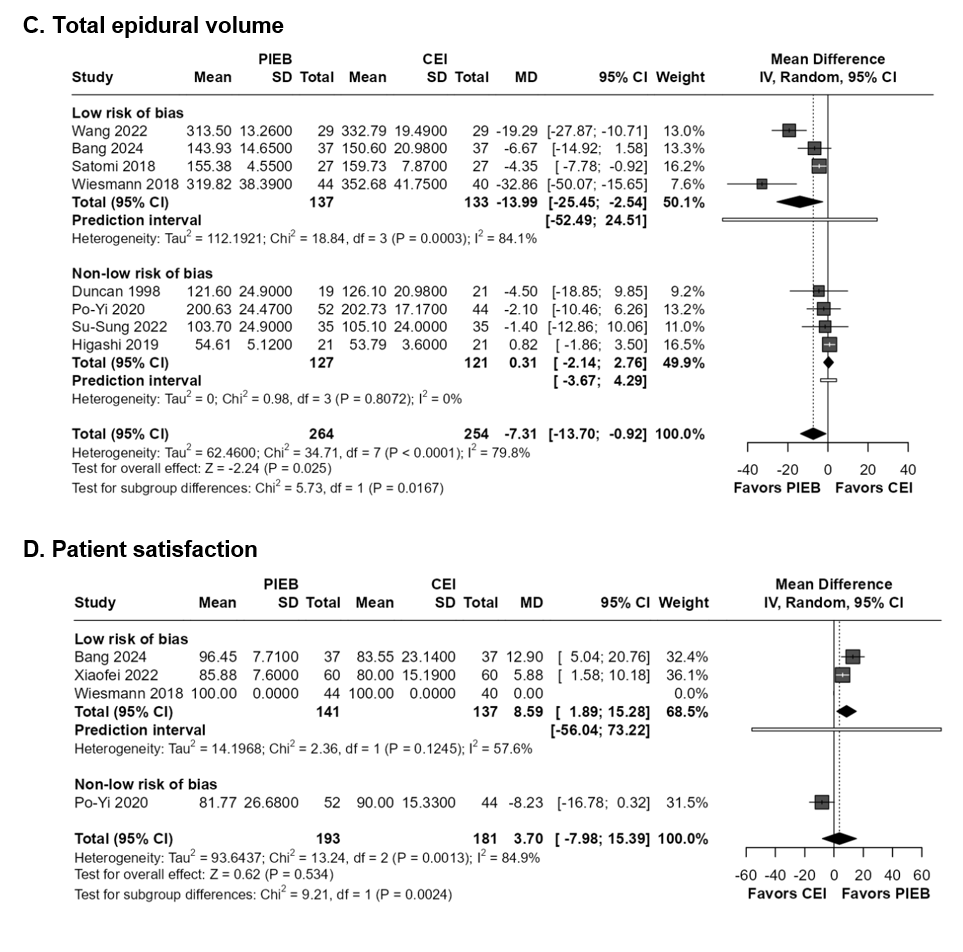
**

*Figure S6.5. Forest plots comparing PIEB and CEI (both associated with patient-controlled epidural analgesia - PCEA) on: C. Total epidural volume administered in mL; D. Patient satisfaction on a visual analog 0-100 scale. Data is stratified according to whether studies were assessed as presenting a low risk of bias or not. CEI: continuous epidural infusion; PIEB: programmed intermittent epidural bolus.*

**Supplemental Figure S6.6. Subgroup analyses: risk of bias stratification**

**
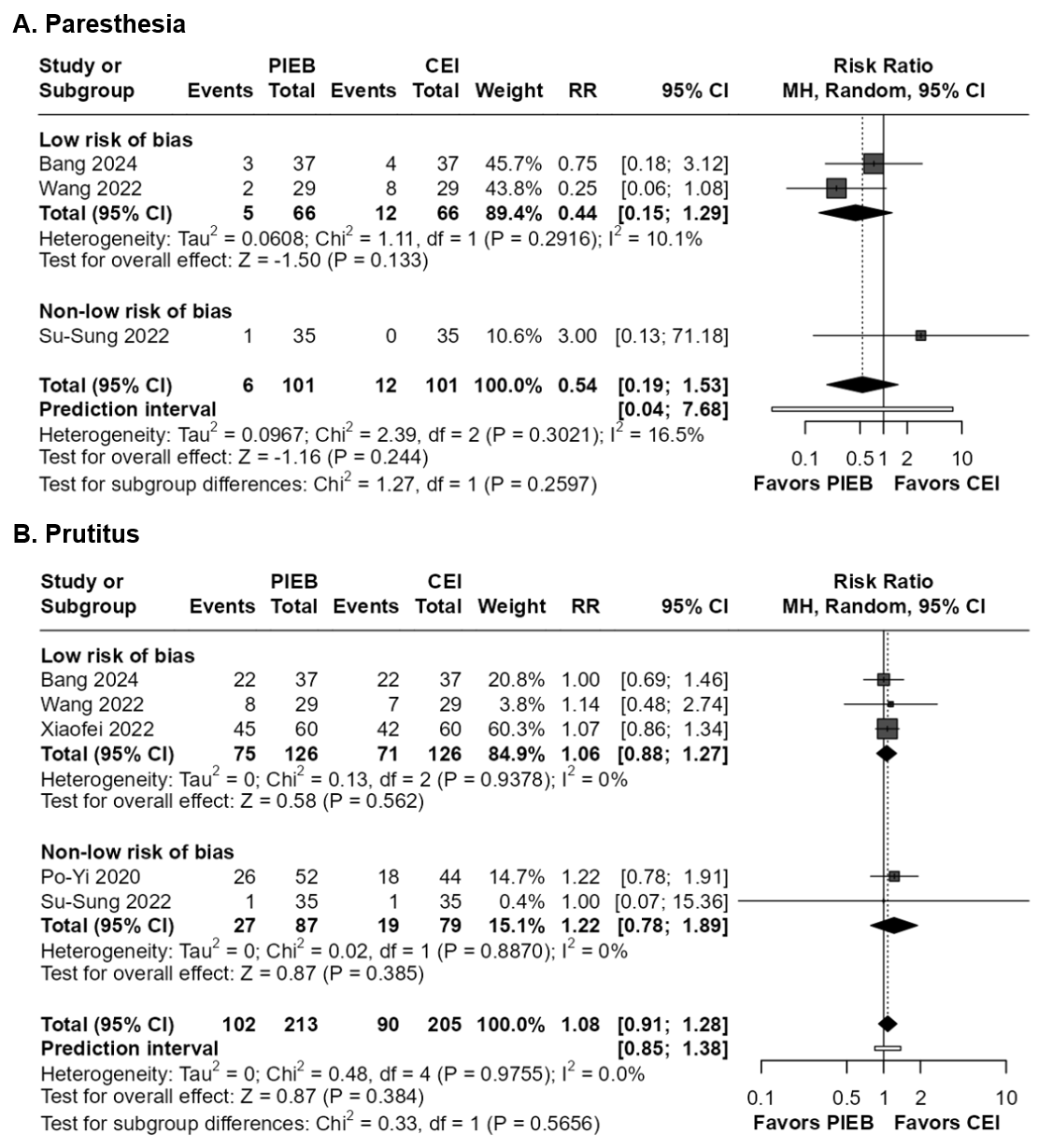
**

*Figure S6.6. Forest plots comparing PIEB and CEI (both associated with patient-controlled epidural analgesia - PCEA) on: A. Patients that reported paresthesia; B. Patients that presented complaints of pruritus. Data is stratified according to whether studies were assessed as presenting a low risk of bias or not. CEI: continuous epidural infusion; PIEB: programmed intermittent epidural bolus.*

**Supplemental Figure S6.7. Subgroup analyses: risk of bias stratification**

**
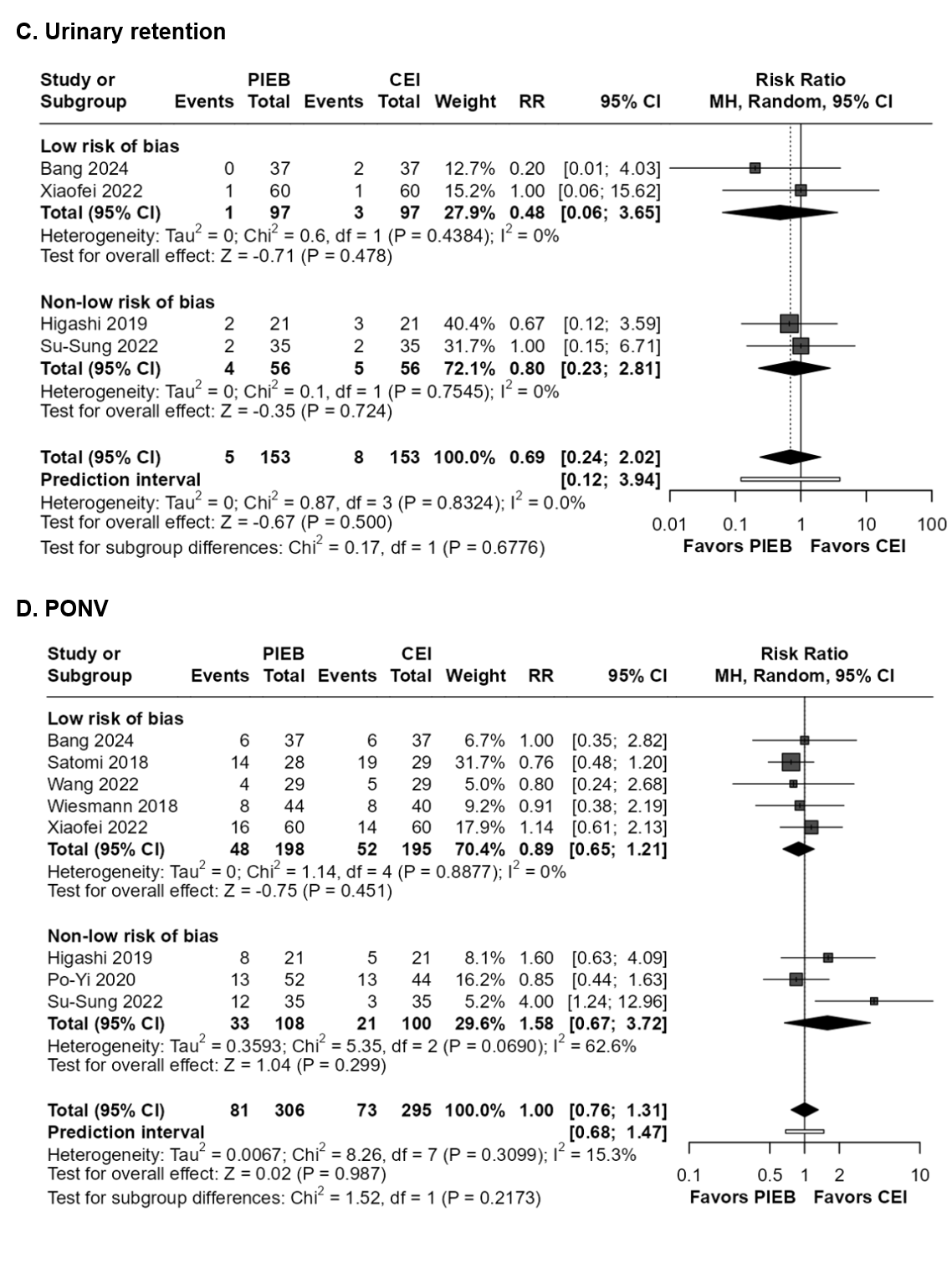
**

*Figure S6.7. Forest plots comparing PIEB and CEI (both associated with patient-controlled epidural analgesia - PCEA) on: C. Patients reporting episodes of urinary retention; D. Patients that presented with postoperative nausea and/or vomiting (PONV). Data is stratified according to whether studies were assessed as presenting a low risk of bias or not. CEI: continuous epidural infusion; PIEB: programmed intermittent epidural bolus.*

**Supplemental Figure S6.8. Subgroup analyses: risk of bias stratification**

**
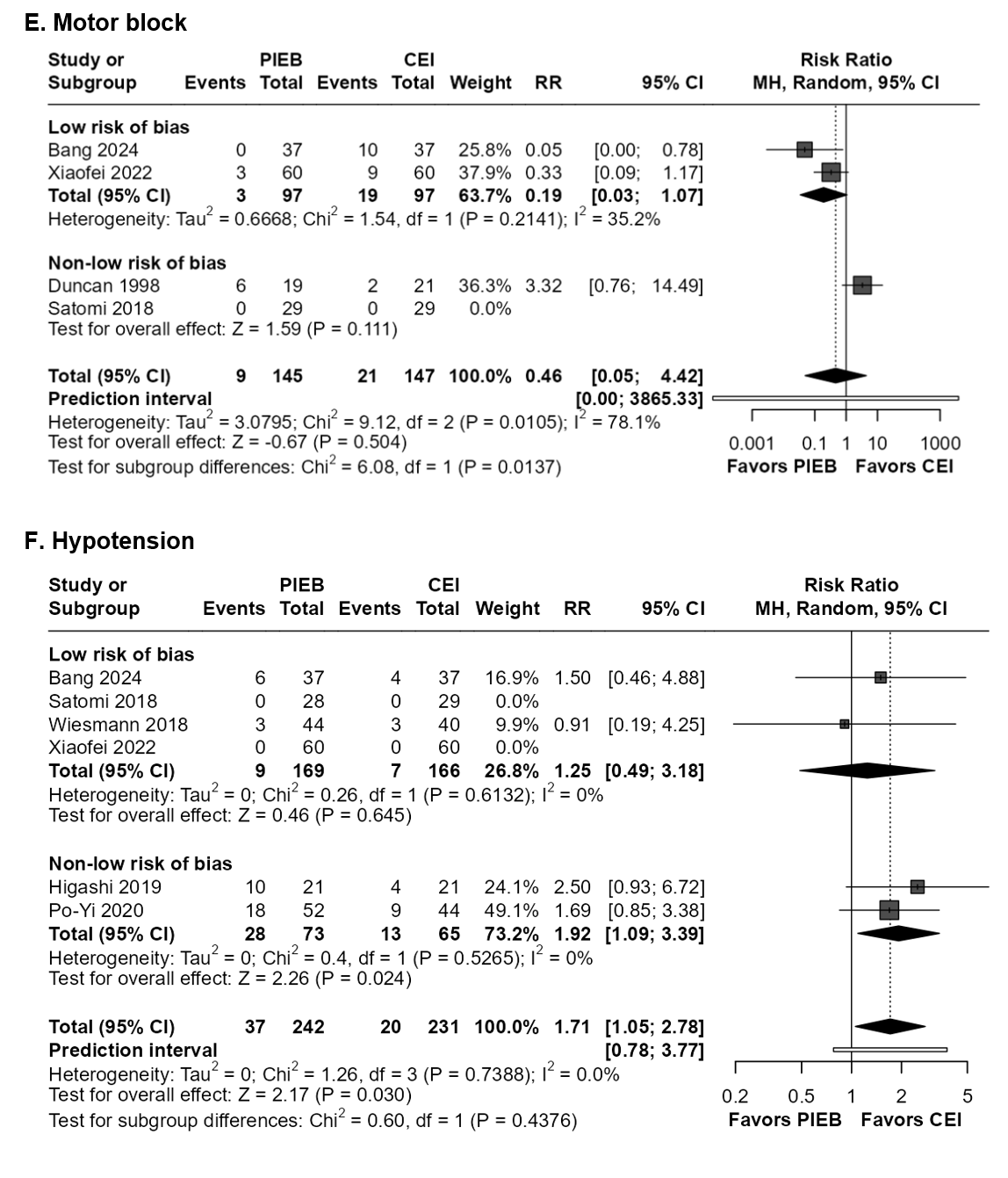
**

*Figure S6.8. Forest plots comparing PIEB and CEI (both associated with patient-controlled epidural analgesia - PCEA) on: E. Patients presenting motor block at 24 hours follow-up; F. the occurrence of postoperative hypotension. Data is stratified according to whether studies were assessed as presenting a low risk of bias or not. CEI: continuous epidural infusion; PIEB: programmed intermittent epidural bolus.*

**Supplemental Figure S7.1. Rest pain at 24 hours: other subgroup analyses**

*
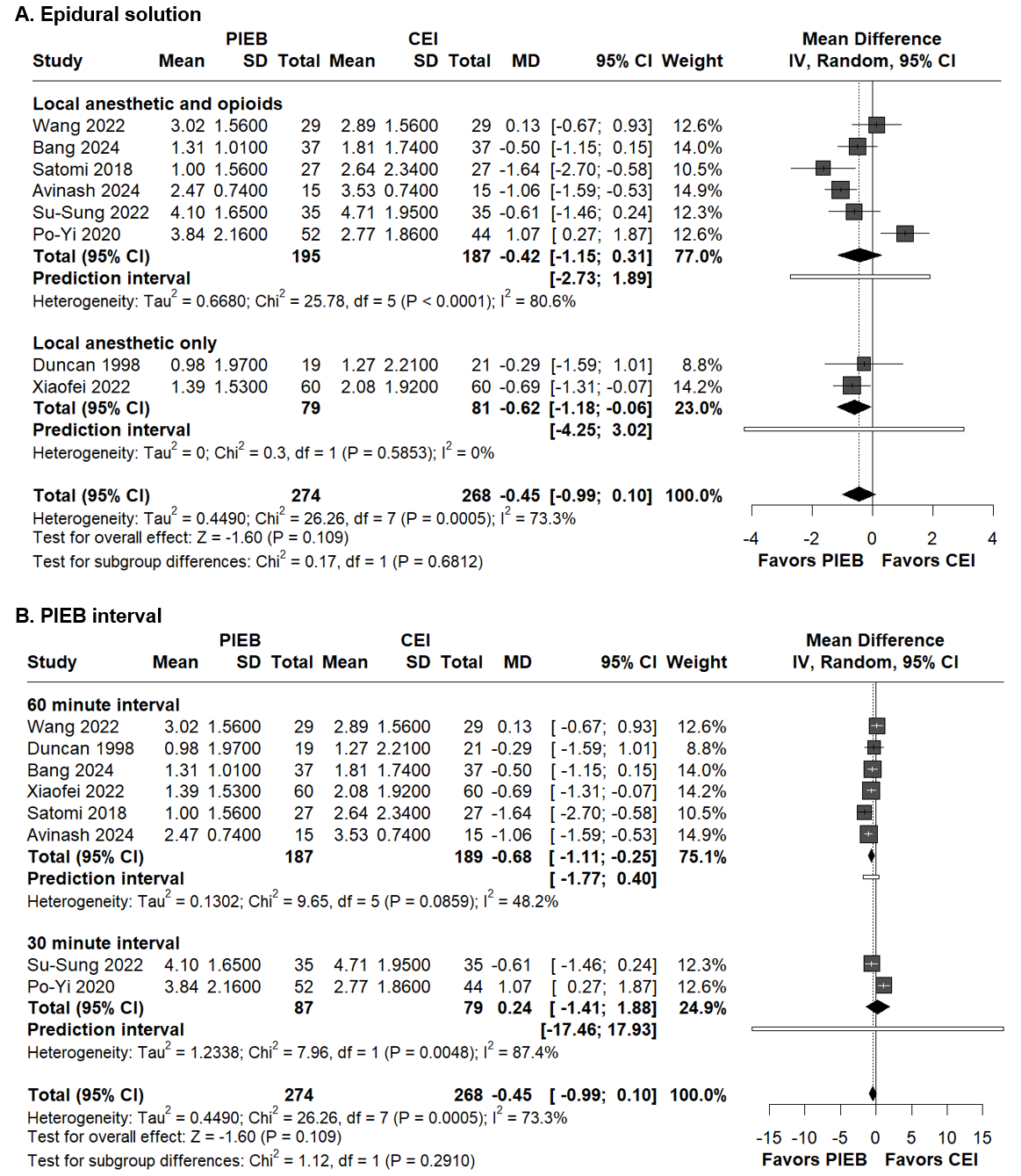
*

*Figure S7.1. Forest plots comparing PIEB and CEI (both associated with patient-controlled epidural analgesia - PCEA) on pain at rest, on a 0-10 scale, at 24 hours. Data is stratified in the following subgroups: A. Pharmacological composition of epidural anesthetic solution; B. Programmed intermittent epidural bolus (PIEB) interval of administration. CEI: continuous epidural infusion.*

**Supplemental Figure S7.2. Rest pain at 24 hours: other subgroup analyses**

*
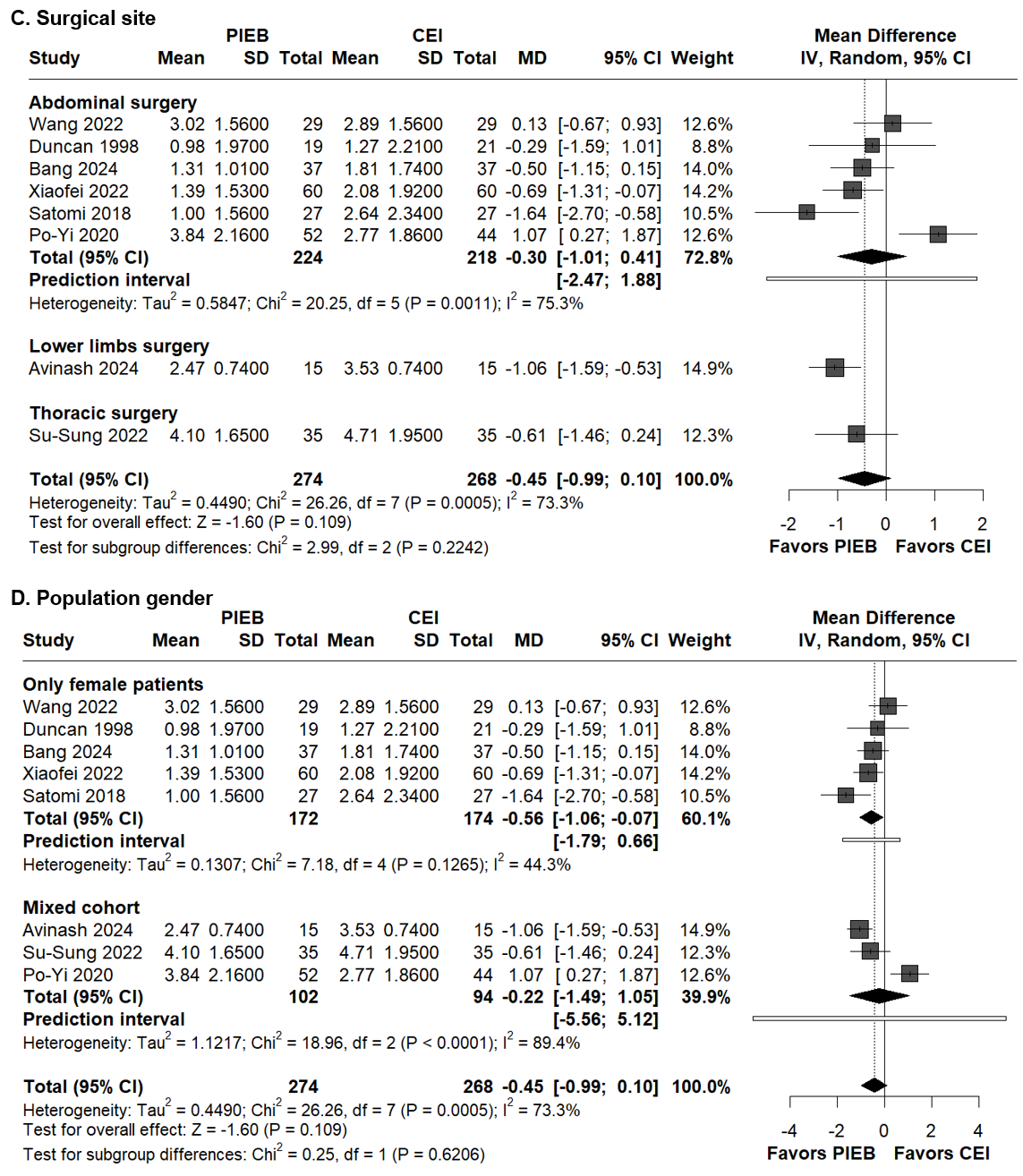
*

*Figure S7.2. Forest plots comparing PIEB and CEI (both associated with patient-controlled epidural analgesia - PCEA) on pain at rest, on a 0-10 scale, at 24 hours. Data is stratified in anatomical location of surgical site (C). CEI: continuous epidural infusion; PIEB: programmed intermittent epidural bolus.*

**Supplemental Figure S8. Rest pain at 24 hours: skewed data subgroup**

*
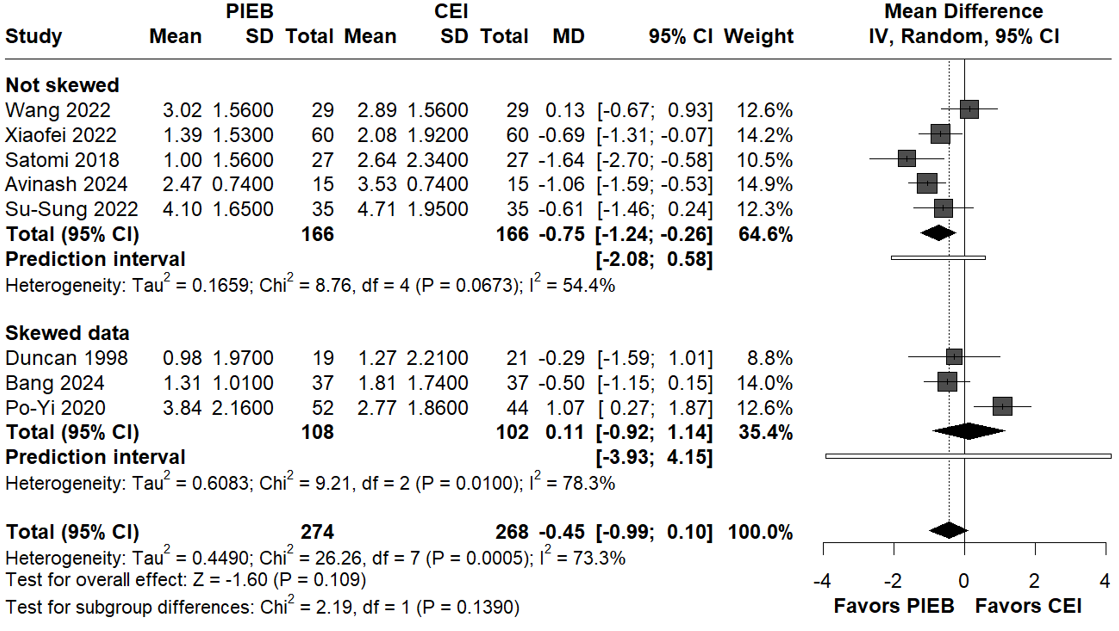
*

*Figure S8. Forest plots comparing PIEB and CEI (both associated with patient-controlled epidural analgesia - PCEA) on pain at rest, on a 0-10 scale, at 24 hours. Data is stratified in subgroups of studies containing either any skewed data after statistical conversion, or those that have only normal distribution. CEI: continuous epidural infusion; PIEB: programmed intermittent epidural bolus.*

**Supplemental Figure S9.1. Secondary outcome subgroup analysis**

**
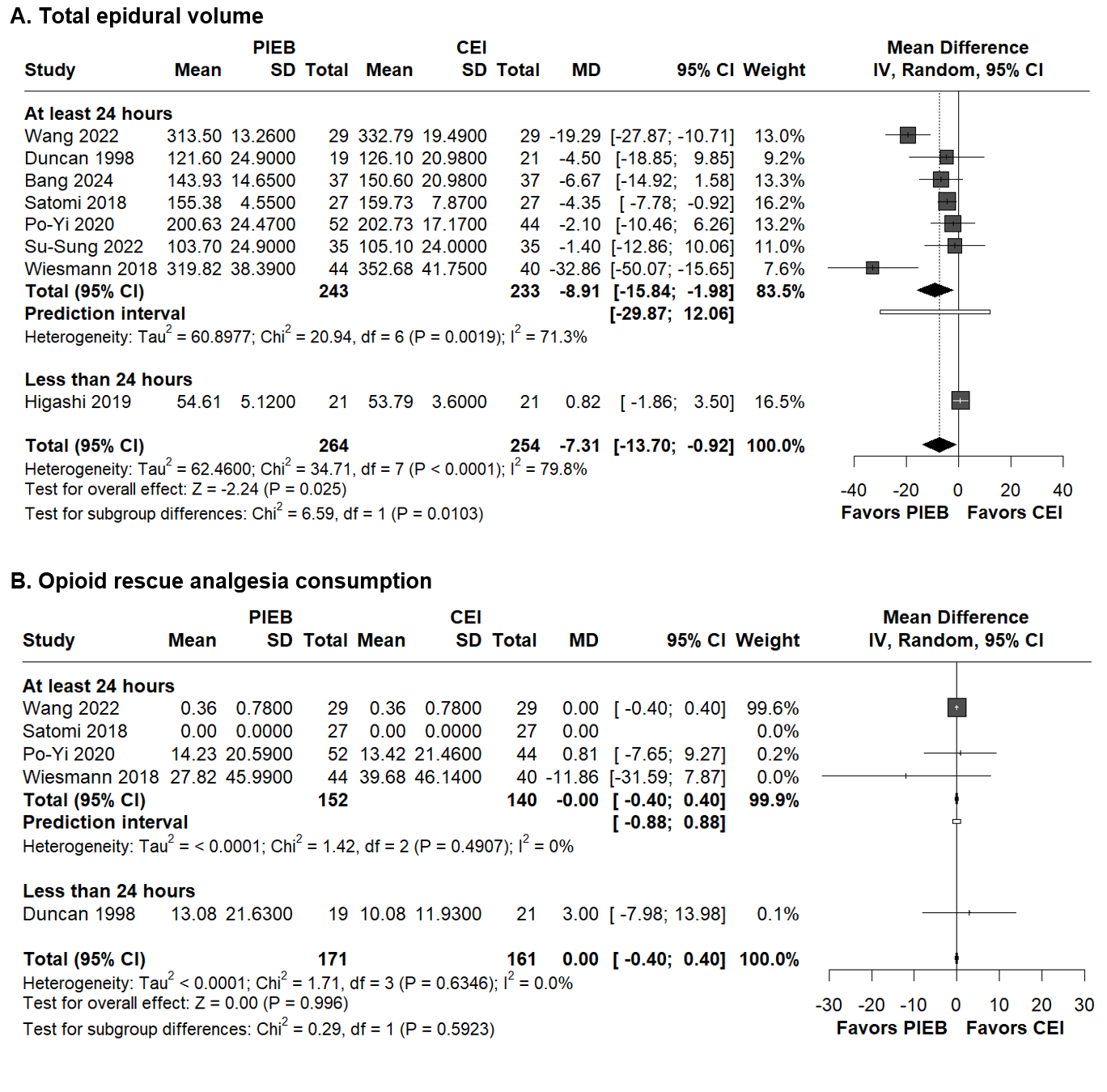
**

*Figure S9.1. Forest plots stratified by follow-up time* *comparing PIEB and CEI (both associated with patient-controlled epidural analgesia - PCEA) on: A. Total epidural volume administered in mL; B. Opioid rescue analgesia consumption in oral morphine milligram equivalents. CEI: continuous epidural infusion; PIEB: programmed intermittent epidural bolus.*

**Supplemental Figure S9.2. Secondary outcome subgroup analysis**

**
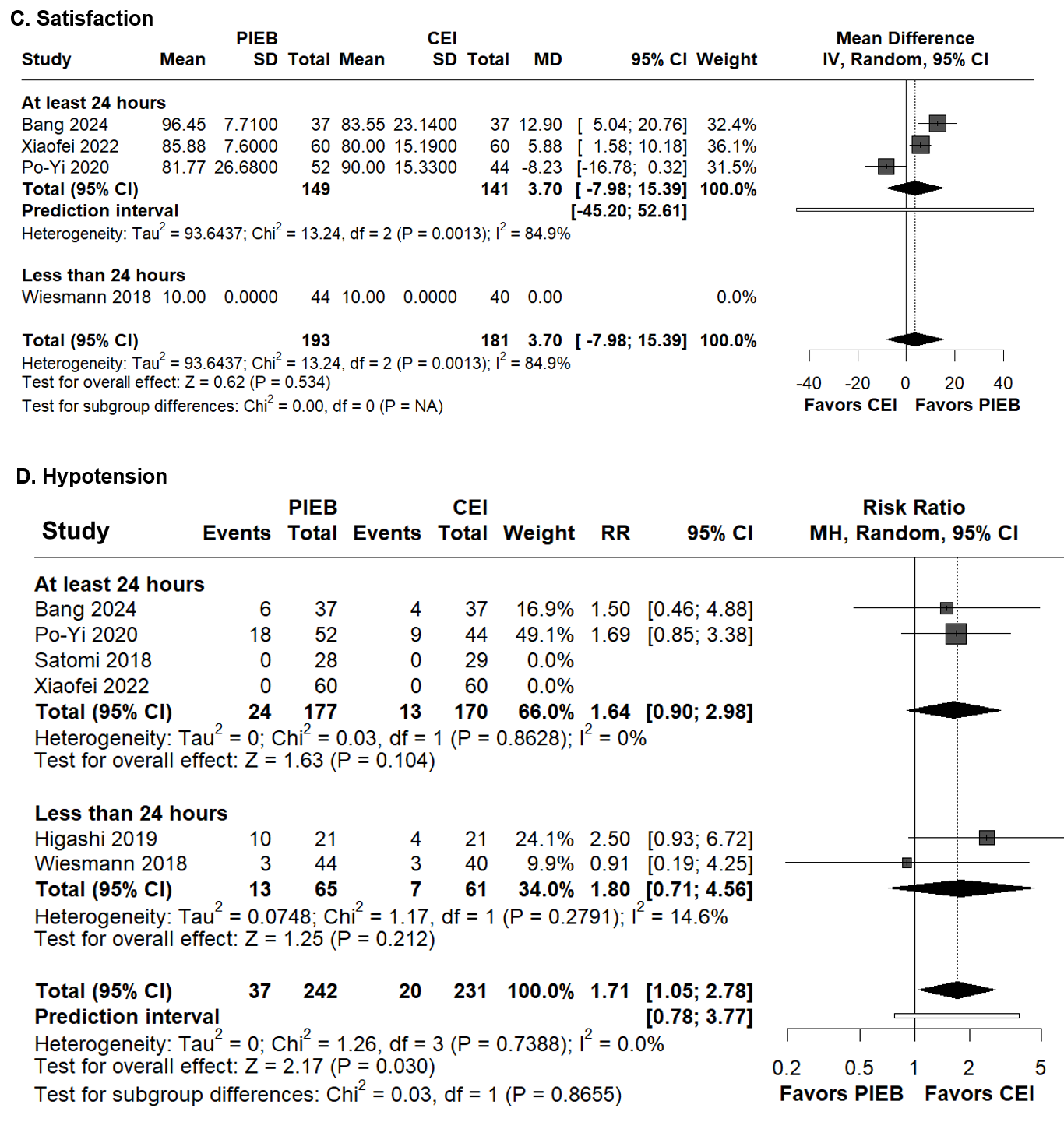
**

*Figure S9.2. Forest plots stratified by follow-up time comparing PIEB and CEI (both associated with patient-controlled epidural analgesia - PCEA) on: C. Patient satisfaction on a visual analog 0-100 scale; D. Patients presenting episodes of hypotension (multiple criteria based on either measured blood pressure or need for vasopressors). CEI: continuous epidural infusion; PIEB: programmed intermittent epidural bolus.*

**Supplemental Figure S9.3. Secondary outcome subgroup analysis**

**
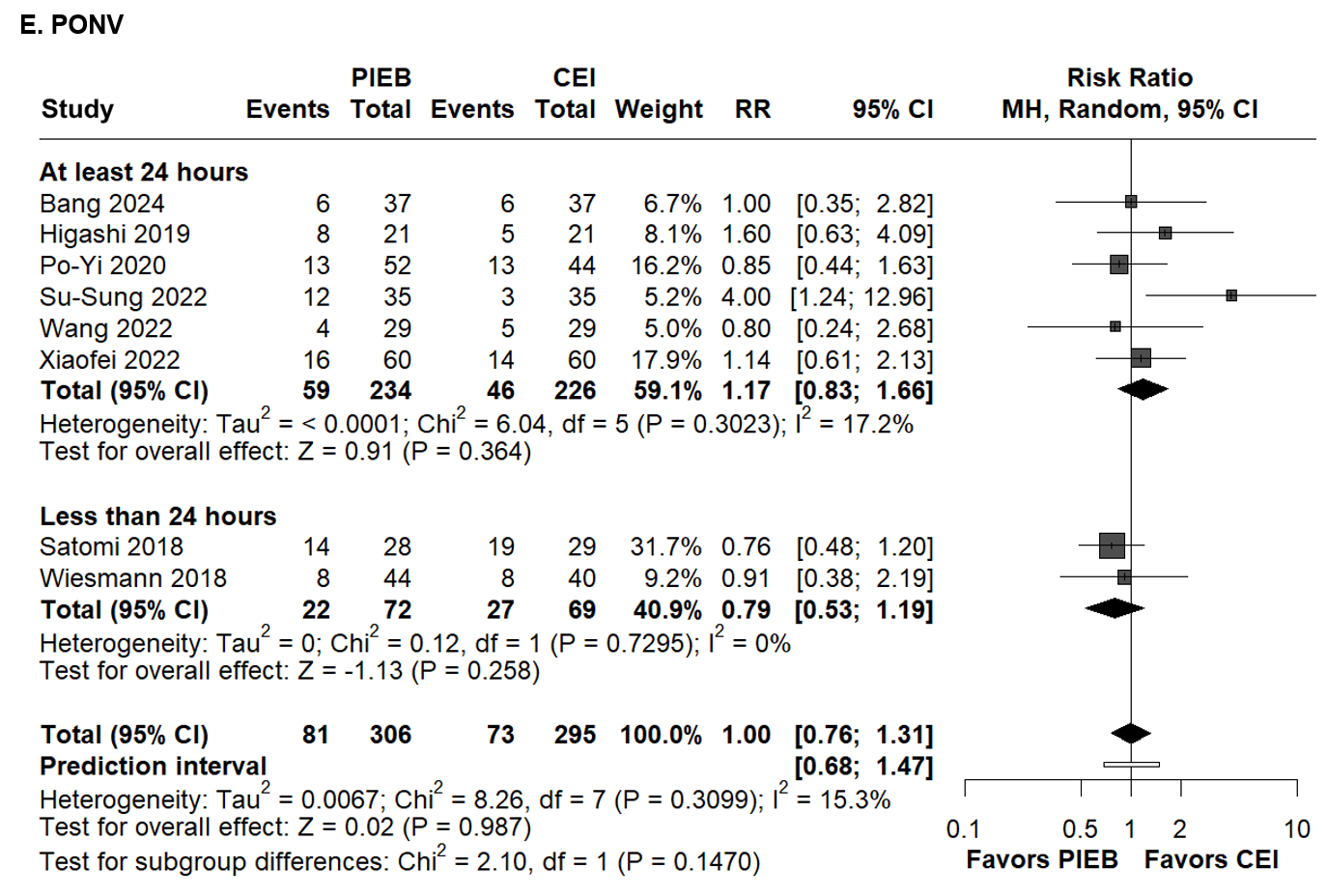
**

*Figure S9.3. Forest plots stratified by follow-up time comparing PIEB and CEI (both associated with patient-controlled epidural analgesia - PCEA) on: E. Patients that presented with postoperative nausea and/or vomiting (PONV). CEI: continuous epidural infusion; PIEB: programmed intermittent epidural bolus.*

**Supplemental Figure S10. Leave-one-out sensitivity analysis**

*
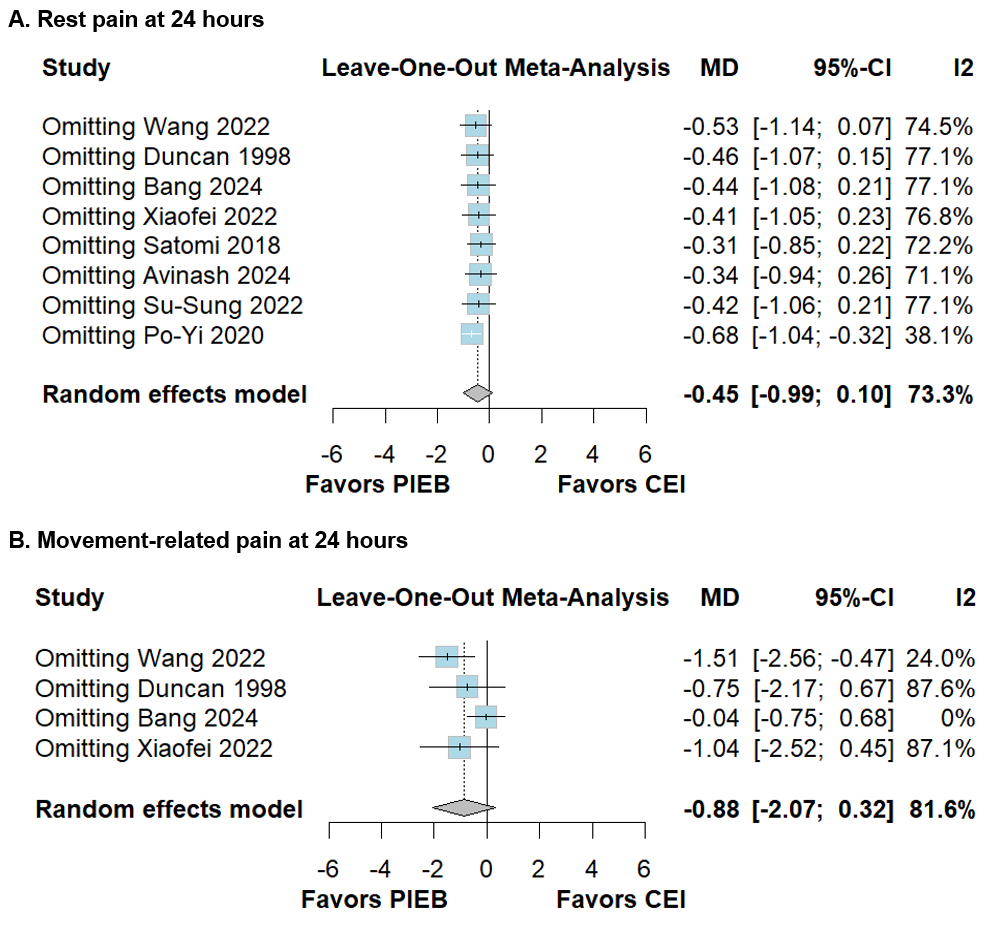
*

*Figure S10. Forest plots presenting results after omitting each study comparing PIEB and CEI (both associated with patient-controlled epidural analgesia - PCEA) on postoperative pain at rest (A) and movement-related pain (defined as pain elicited by movement) (B), both at 24 hours and on a 0-10 scale. CEI: continuous epidural infusion;* *PIEB: programmed intermittent epidural bolus.*

**Supplemental Figure S11.1. Funnel plots: pain at rest**

**
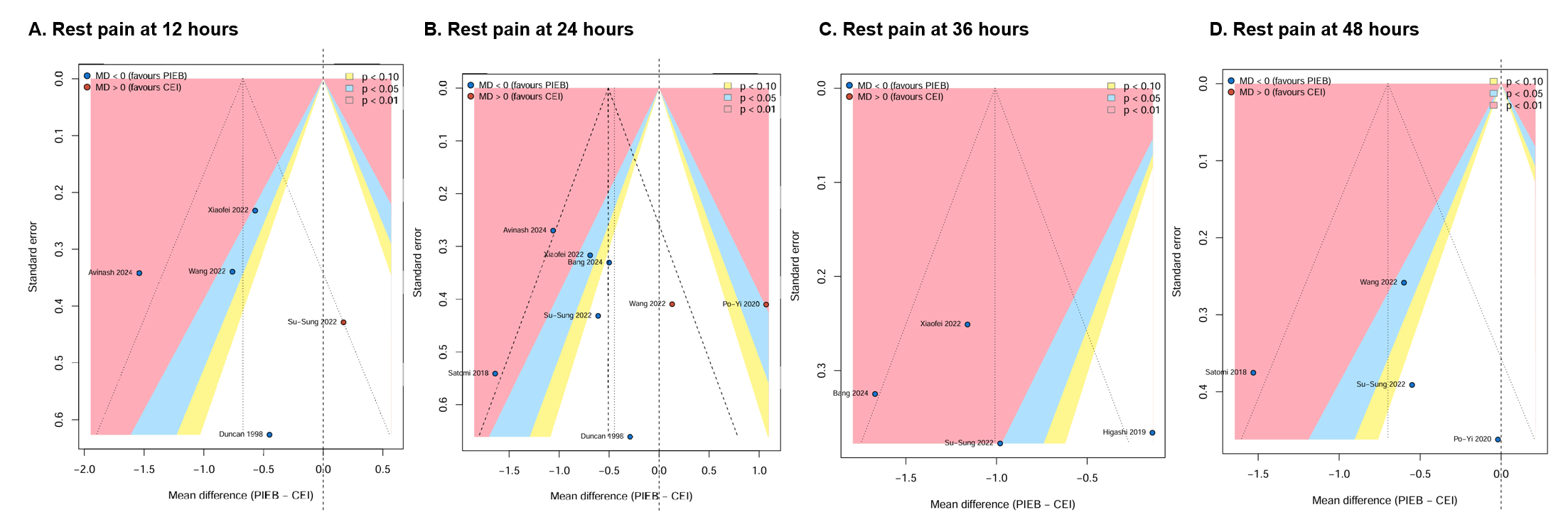
**

*Figure S11.1. Contour-enhanced funnel plots assessing small-study effects for randomized trials comparing programmed intermittent epidural bolus (PIEB) and continuous epidural infusion (CEI), both combined with patient-controlled epidural analgesia (PCEA), for postoperative pain at rest at 12 (A), 24 (B), 36 (C), and 48 (D) hours. The x-axis represents the mean difference (PIEB − CEI), with negative values favoring PIEB, and the y-axis represents the standard error. Shaded regions correspond to statistical significance contours (p < 0.10, p < 0.05, and p < 0.01). The vertical dashed line indicates the pooled effect estimate.*

**Supplemental Figure S11.2. Funnel plots: movement-related pain**

**
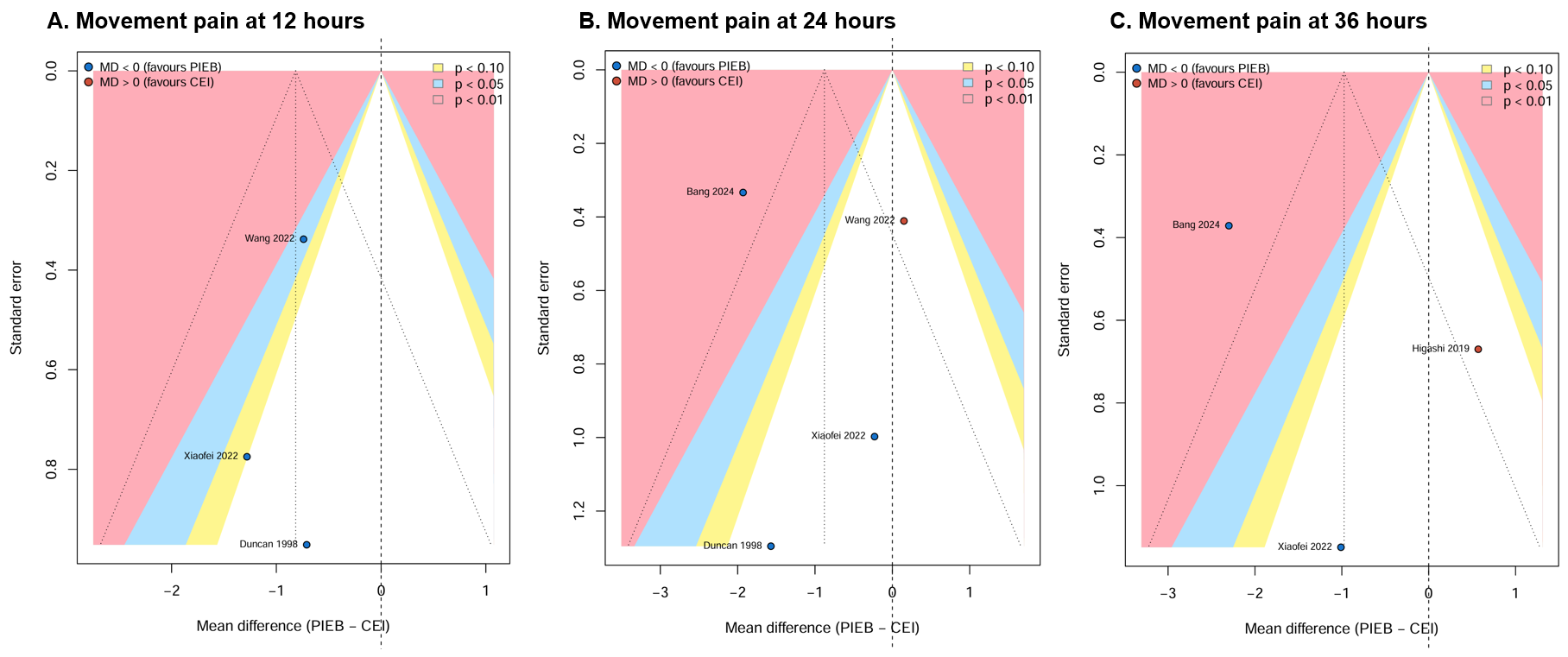
**

*Figure S11.2. Contour-enhanced funnel plots assessing small-study effects for randomized trials comparing programmed intermittent epidural bolus (PIEB) and continuous epidural infusion (CEI), both combined with patient-controlled epidural analgesia (PCEA), for postoperative movement-related pain at 12 (A), 24 (B), and 36 hours (C). The x-axis represents the mean difference (PIEB − CEI), with negative values favoring PIEB, and the y-axis represents the standard error. Shaded regions correspond to statistical significance contours (p < 0.10, p < 0.05, and p < 0.01). The vertical dashed line indicates the pooled effect estimate.*

**Supplemental Figure S11.3. Funnel plots: other continuous outcomes**

**
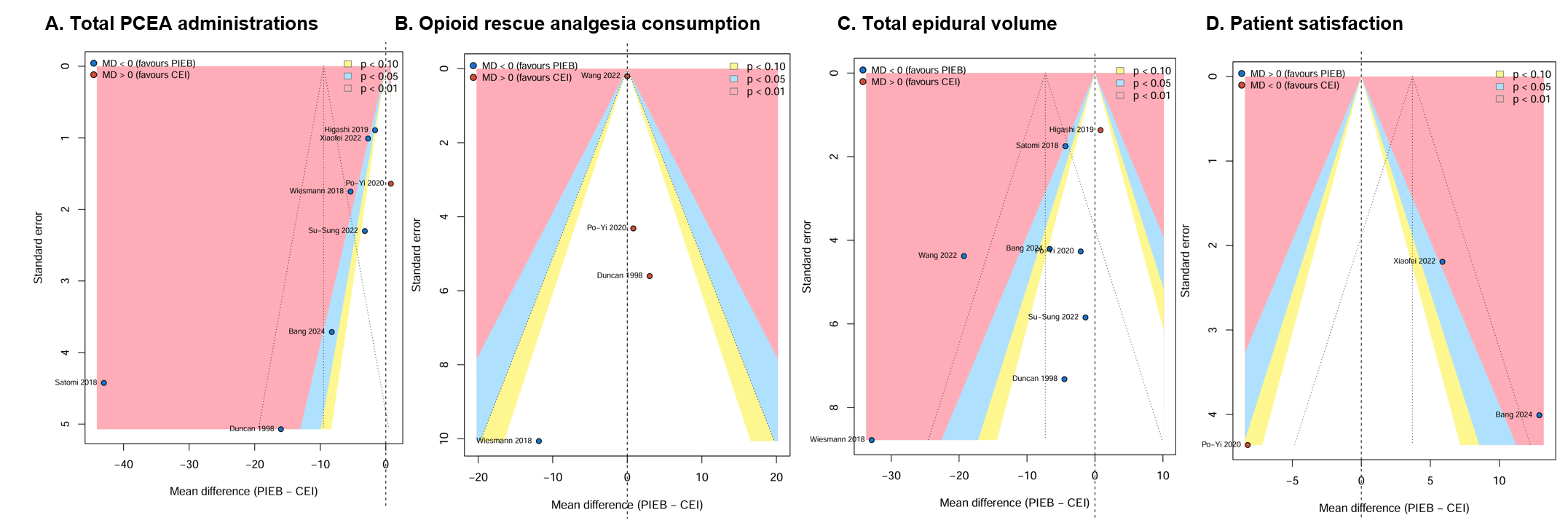
**

*Figure S11.3. Contour-enhanced funnel plots assessing small-study effects for randomized trials comparing programmed intermittent epidural bolus (PIEB) and continuous epidural infusion (CEI), both combined with patient-controlled epidural analgesia (PCEA), for the following postoperative outcomes: total PCEA administrations (A); opioid rescue analgesia consumption (B); total epidural volume infused (C); and, patient satisfaction, on a 0-100 scale (D).The x-axis represents the mean difference (PIEB − CEI), with negative values favoring PIEB in all outcomes except satisfaction, and the y-axis represents the standard error. Shaded regions correspond to statistical significance contours (p < 0.10, p < 0.05, and p < 0.01). The vertical dashed line indicates the pooled effect estimate.*

**Supplemental Figure S11.4. Funnel plots: binary outcomes**

**
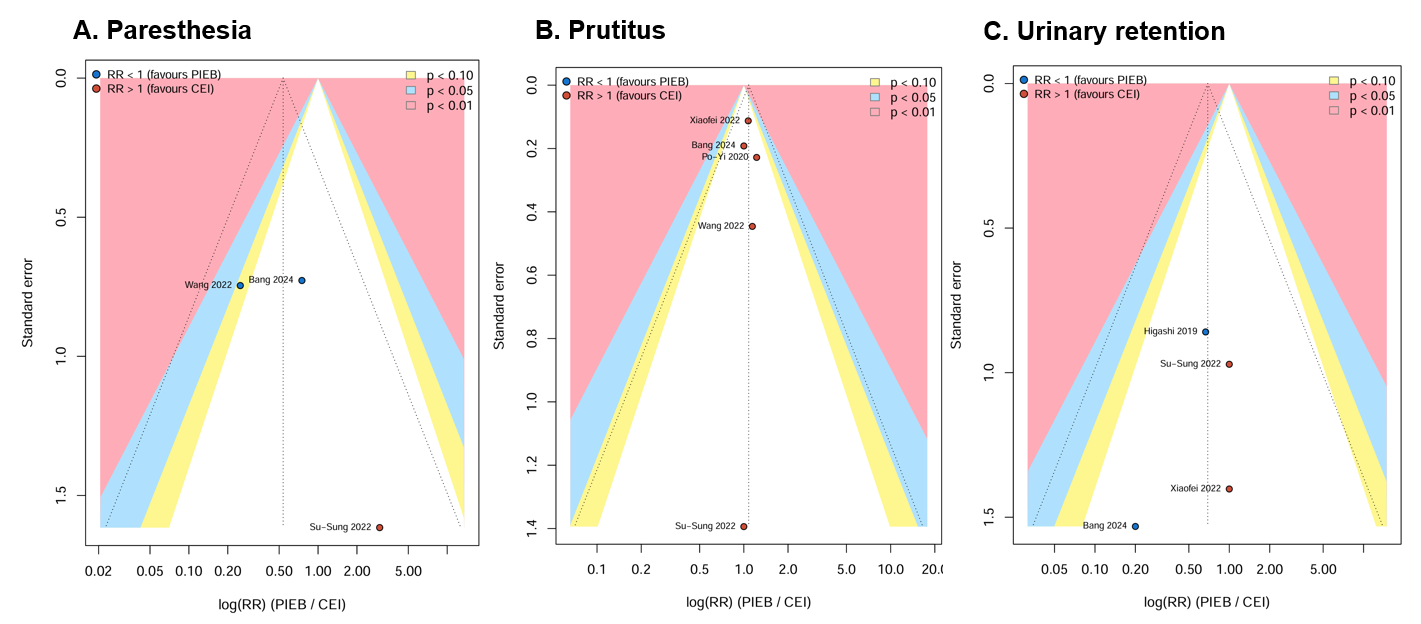
**

*Figure S11.4. Contour-enhanced funnel plots assessing small-study effects for randomized trials comparing programmed intermittent epidural bolus (PIEB) and continuous epidural infusion (CEI), both combined with patient-controlled epidural analgesia (PCEA), postoperative occurrence of paresthesia (A), pruritus (B), and urinary retention (C). The x-axis represents the mean difference (PIEB − CEI), with negative values favoring PIEB, and the y-axis represents the standard error. Shaded regions correspond to statistical significance contours (p < 0.10, p < 0.05, and p < 0.01). The vertical dashed line indicates the pooled effect estimate.*

**Supplemental Figure S11.5. Funnel plots: binary outcomes**

**
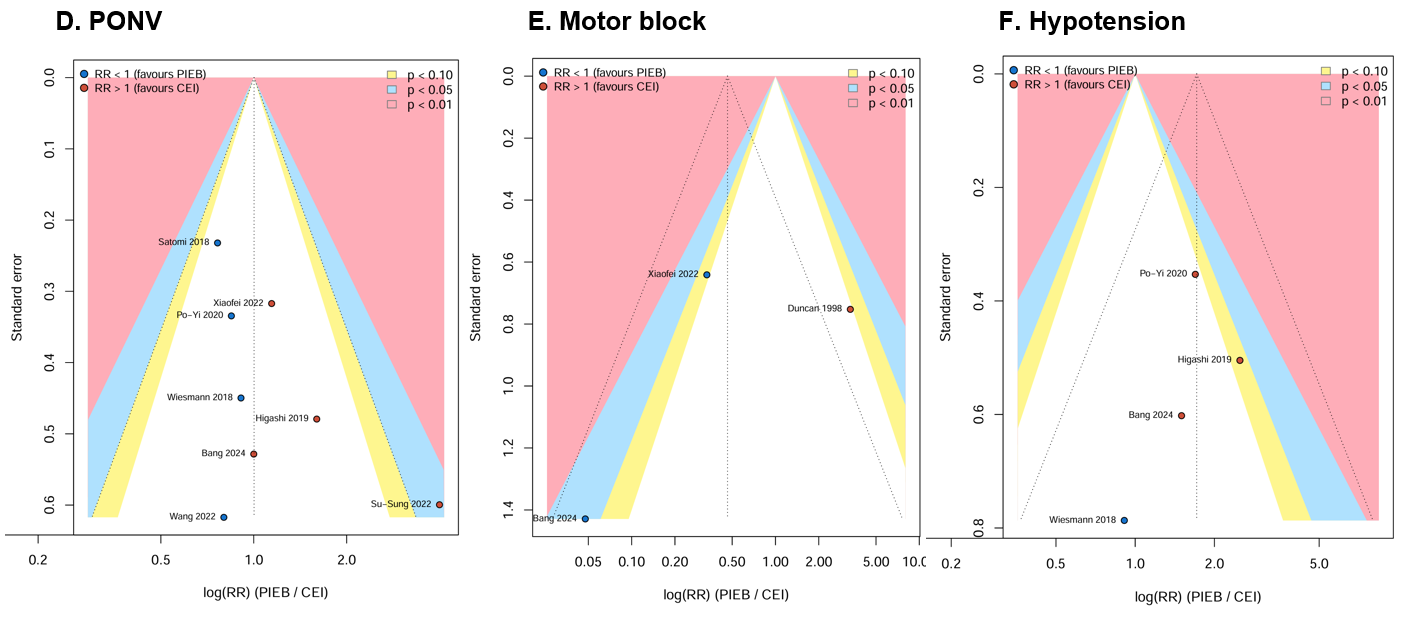
**

*Figure S11.5. Contour-enhanced funnel plots assessing small-study effects for randomized trials comparing programmed intermittent epidural bolus (PIEB) and continuous epidural infusion (CEI), both combined with patient-controlled epidural analgesia (PCEA), postoperative occurrence of nausea and vomiting (PONV; D), motor block (E), and hypotension (F). The x-axis represents the mean difference (PIEB − CEI), with negative values favoring PIEB, and the y-axis represents the standard error. Shaded regions correspond to statistical significance contours (p < 0.10, p < 0.05, and p < 0.01). The vertical dashed line indicates the pooled effect estimate.*
